# Position-dependent codon occurrence is associated with patterns of translational efficiency and ribosomal occupancy in the human transcriptome

**DOI:** 10.1371/journal.pcbi.1014501

**Published:** 2026-07-20

**Authors:** Kaavya Subramanian, Nathan Waugh, Cole Shanks, David A. Hendrix

**Affiliations:** 1 School of Electrical Engineering and Computer Science, Oregon State University, Corvallis, Oregon, United States of America; 2 Department of Biochemistry and Biophysics, Oregon State University, Corvallis, Oregon, United States of America; Istituto Europeo di Oncologia, ITALY

## Abstract

All life depends on the reliable translation of RNA to protein according to complex interactions between translation machinery and RNA sequence features. While ribosomal occupancy and codon frequencies vary across coding regions, well-established metrics for computing coding potential of RNA do not capture such positional dependence. Here, we investigate positional bias in codon usage, which contextually accounts for the position of protein-coding signals embedded within coding regions. We demonstrate the existence of position-dependent patterns of codon frequency in the human transcriptome and describe these patterns using our POsition-Specific Codon Occurrence (POSCO) score that is more consistently associated with translation-initiating codons than other common sequence features. We further show that the patterns described by POSCO are not accounted for by other common scores, including position-dependent GC content, consensus sequences, and the presence of signal peptides in the translation product. More importantly, POSCO defines a spectrum of translational efficiency and local tRNA adaptation index (tAI). High POSCO scores correspond to the highest initial tAI values, which increase over the downstream length of the transcript, forming a translational highway. Meanwhile, low POSCO scores exhibit the lowest initial tAI values followed by a previously undescribed translational valley. An inverse correlation was found between POSCO score and ribosomal occupancy near the start codon. We also find that POSCO defines a spectrum of local folding energies, with high-POSCO transcripts showing the most stable folding immediately after the start codon. Finally, we examine the relationship between POSCO intensity and functional enrichment. We find that transcripts with start codons showing the highest POSCO are enriched for functions relating to development of musculoskeletal, cardiovascular, neurological, gastrointestinal, sensory, and other body systems. Furthermore, transcripts with high POSCO are depleted for functions related to immune response and detection of chemical stimulus. These findings lay important groundwork to improve our understanding of the regulation of translation, the calculation of coding potential, and the classification of RNA transcripts.

## Author summary

This study focuses on a feature of RNA translation called position-specific codon occurrence (POSCO), which refers to how the use of codons varies along the length of a coding sequence. While POSCO has been observed in some organisms like bacteria and fungi, it has been less studied in animals, especially humans. The research shows that a score that quantifies POSCO is associated with translation start sites more than commonly used features. It also demonstrates that POSCO is not explained by other factors like GC content or signal peptides in the protein product. Importantly, POSCO correlates with ribosomal occupancy and position-dependent trends in tRNA adaptation index (tAI) scores, which suggests that it encapsulates sequence features related to translational efficiency. High POSCO scores are linked to higher tAI values, suggesting more efficient translation, while low POSCO scores correspond to inefficient translation early in the process. This finding is corroborated by low relative ribosomal occupancy near the start codon, and higher translational efficiency for the highest POSCO transcripts. Additionally, high POSCO transcripts have stronger local folding near the start codon, suggesting a mechanism of efficient regulation of ribosomal movement. Genes with high POSCO are associated with developmental biology, while those with low POSCO are involved in immune and chemical detection functions. This research offers new insights into how translation is regulated and provides a better framework for understanding RNA coding potential.

## Introduction

Translation from RNA to protein is a fundamental and ubiquitous life process. Some studies estimate that ≈68% of human genes do not encode proteins, and are transcribed as long noncoding RNAs (lncRNAs) or transcripts of unknown coding potential [1]. This raises the question of how the ribosome distinguishes mRNAs from lncRNAs that have open reading frames (ORFs) [2]. At the same time, other studies suggest that some lncRNAs are weakly translated and therefore may be misannotated [3–5]. It has been observed that as many as 40% of human mRNAs contain upstream AUGs (uAUGs) [6] and in more recent transcriptome models it is closer to 50% [7]. These observations underscore the importance of understanding how the ribosome uses sequence features to distinguish mRNAs from lncRNAs, as well as to properly identify the start codon within an mRNA. Such sequence features could help explain the translation of short transcripts that encode small peptides [8,9], and improve the design of mRNA vaccines [10].

The Kozak consensus sequence is one such motif, discovered by Marilyn Kozak in the 1980s [11]. This sequence characterizes and helps identify start codons in eukaryotic mRNAs and is regarded as a key feature that enhances translational potential. Another approach to quantifying mRNA coding potential is codon usage bias (CUB), which refers to the frequency of usage for each codon in the coding portion of a transcriptome relative to the frequency of synonymous codons. Computational models of CUB have been shown to perform well in predicting translational efficiency and also show an association with ribosome profiling data [12]. The codon adaptation index (CAI) is a widely recognized metric of CUB that assigns a score to each transcript based on its length and codon composition relative to overall CUB across the coding transcriptome [13]. The CAI model has been used as a baseline for other quantitative models of CUB, and has also been correlated with gene expression in select genomes [13]. Another common metric used is tRNA adaptation index (tAI), which is describes how much the codons of a gene utilize available tRNAs [14,15]. This score is computed from a geometric mean of weights based on the tRNA availability and with adjusted contributions for wobble interactions [14,16].

A potential limitation of these approaches is that they do not consider the role of codon position explicitly. Studies over 20 years ago have identified clusters of otherwise rare codons at the 5’ ends of open reading frames in prokaryotes and eukaryotes [17–20]. Studies of position-dependent codon usage bias (POSCO) observed that codon usage is non-uniform with regard to transcript position in *E. coli* [21] and yeast [22]. Comparative studies across the tree of life have observed correlations between codon usage patterns and local mRNA folding energy, but generally observed a region of weak folding at the 5’ and 3’ ends of the ORF [23]. Rare codon usage around the beginning of ORFs may in part be related to controlling mRNA folding around the start codon, with folding in the first 30–40 codons can inhibit ribosome sequestration in prokaryotes, eukaryotes and archaea [24]. Furthermore, studies in yeast and mammals have identified that strong folding 14–30 codons after the start codon is under selection, possibly by either increasing the probability that the start codon is recognized by blocking movement of the pre-initiation complex [25–28], preventing the folding of the region upstream that overlaps the start codon, or delaying the ribosome at the beginning to prevent traffic jams [24,29,30]. Moreover, our investigations demonstrated that a recurrent neural network, which was trained to distinguish human mRNAs from lncRNAs based on sequence alone, was able to learn sequence-specific rules and make classification decisions approximately 100–200 nucleotides (nt) downstream of the start codon [31]. A remaining challenge is a holistic characterization of POSCO and to examine it in a broader biological context of translational regulation and gene function.

Here, we explore the role of positional bias in codon usage within the human coding transcriptome and whether position-specific patterns of codon usage are a defining feature of human ORFs. We present the POsition-Specific Codon Occurrence (POSCO) score that incorporates these position-dependent codon patterns, using only information observed in the first 300 nt downstream of a given AUG. We show that our score shows a more consistent correspondence to start codons compared to non-start AUGs than other common sequence descriptors. While the POSCO score was constructed from general positional trends in codon usage, we show that it is correlated with translational efficiency data. Within the human transcriptome, we show that the highest POSCO transcripts are associated with the highest initial tAI values, steepest initial tAI-visualized slopes, and the highest steady-state tAI values, which we describe as a “translational highway”. Meanwhile, the lowest POSCO transcripts are associated with the lowest initial tAI values, the lowest downstream steady-state tAI values, and between the two, a “translational valley” the extent to which has not been described before to our knowledge. High-POSCO transcripts show a lower energy, more stable folding immediately after the start codon. Moreover, we show that high- and low-POSCO transcripts display a clear division in biological function. Notably, unlike typical CUB methods score codon usage relative to other synonymous codons or to the most frequent synonymous codon, POSCO compares to the global frequency of the associated triplet, providing a different relative weighting of codon frequencies.

## Results

### Visualization of position-dependent patterns of codon occurrence

**Fig 1:**
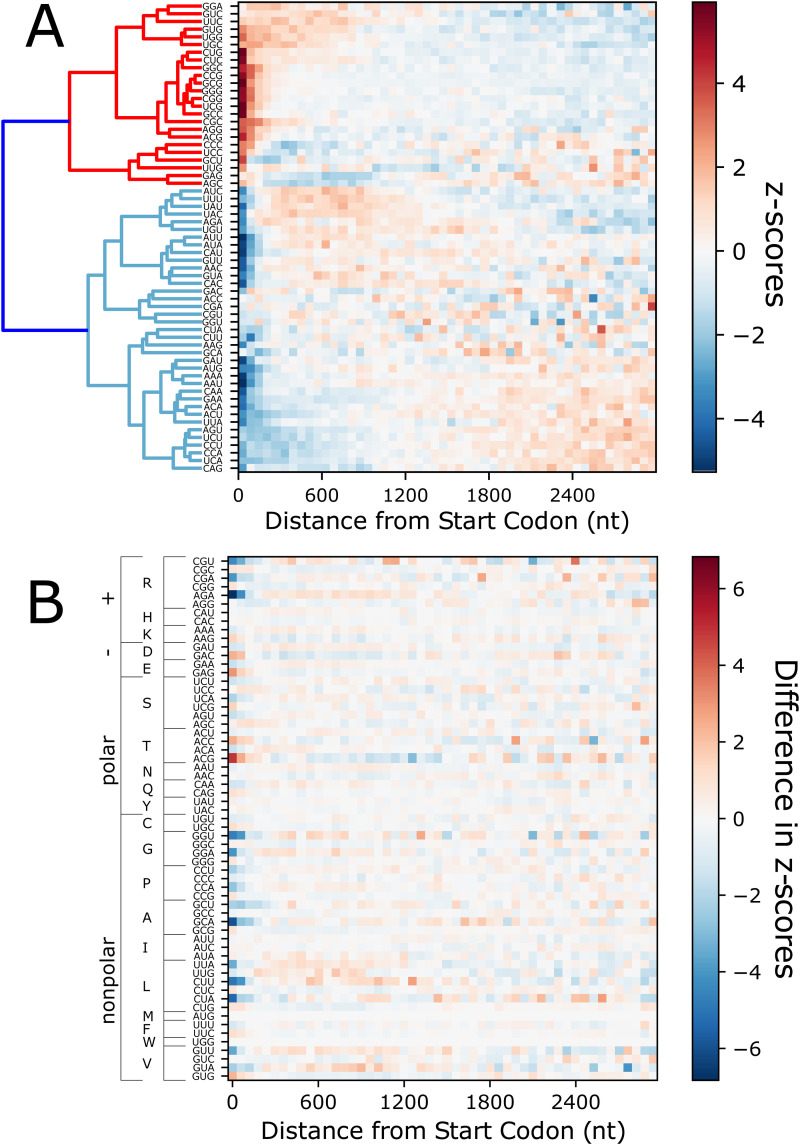
A. A “bird’s eye view” of position-dependent codon usage bias in the human transcriptome (GENCODE protein-coding transcript models). A heatmap and dendrogram visualizing the z-scores computed from codon occurrence frequency in bins of size 60 nt. **B.** Heatmap showing the differences in z-scores between synonymously shuffled transcripts and the original sequences for each codon c and bin b, $z_{shuffled}(c,b)\;-\;z(c,b)$.

We first visualized coarse-grained positional codon biases in the human transcriptome using GENCODE data [7]. The frequencies of all 61 sense codons in protein-coding transcripts were visualized in successive 60-nt bins, up to 3000 nt downstream of the annotated start codon. We transformed the observed bin-specific codon frequencies to z-scores, defined relative to the global average frequency of codon occurrences within a bin, across the 50 bins downstream of the AUG. We observed that most codons displayed a distinct position-dependent z-score profile as we move downstream from the start codon, with some codons increasing in usage and others decreasing (Fig 1A).

We investigated whether the heatmap of z-score profiles reveals other patterns in position-dependent codon usage when the codons are clustered according to distinct criteria. First, we organized codons by their amino acid chemical properties, but did not observe a strong correspondence between z-score profile and encoded amino acid chemical type (S1 Fig) and with a zoomed in view it is clear that alanine (A), glutamic acid (E), and serine (S) are strongly enriched in the first codon, while others show other dynamic trends (S2 Fig). We next performed hierarchical clustering on the codons and found that they were predominantly organized by G and C (GC) nucleotide content. To test this observation, we compared our computed z-score dendrogram distance against a modified Hamming distance that compares the extent of variation of GC nucleotides between two codons. We found a strong correspondence between the number of common GC nucleotides and the dendrogram distance in Fig 1A, suggesting that the GC content of each codon is indeed a significant factor in the pattern of position-dependent z-scores (S3 Fig).

Next, we grouped codons by their GC content and plotted z-score curves for each individual codon (S4 Fig). We found that codons consisting exclusively of GC nucleotides show a strong enrichment near the start codon, while codons consisting exclusively of A or U (AU) nucleotides are depleted immediately after the start codon. Codons with either 1 or 2 GC nucleotides reflect these trends in intermediate fashion, with GC-rich codons (2 GC nucleotides per codon) showing greater similarity to GC-only codons, while GC-poor codons (1 GC nucleotide per codon) show greater similarity to AU-only codons.

We next computed the codon z-score heat map for synonymous codon-shuffled coding sequences (CDS) such that for each transcript, the codon occurrences for each amino acid are shuffled while preserving encoded amino acid sequences and codon frequencies (S5 Fig). We found that after shuffling, the position-dependent enrichment observed for very GC-rich codons (e.g., with three GC nucleotides) had been distributed amongst the other synonymous codons. We next analyzed the position dependence of amino acids via the same type of z-score heat map as in S1 Fig and arranged by amino acid content (S6 Fig). We found that the same amino acids were enriched as in the synonymous codon-shuffled CDS heatmap. In some cases, such as Valine (V) the amino acid showed no enrichment at the first codon, but associated codons showed both enriched and depleted codons that cancel each other at the amino acid level. We then computed the change in z-score (shuffled - original) and found that codons showing a positive change in enrichment had an G or a C in the third position (GC3) (Fig 1B). Some codons, such as GUG encoding valine (V), show an enrichment compared to shuffling even though their original z-score is depleted near the start codon, suggesting they are less depleted than synonymous codons. This is consistent with GC3 being a common feature of codons that show a position bias toward the 5’ end of the ORF. Overall, these observations suggest that GC3 codons have the highest enrichment compared to synonymous codons, which is a type of POSCO that continues 200–300nt from the start codon.

### Optimizing a position-specific codon scoring matrix

**Fig 2:**
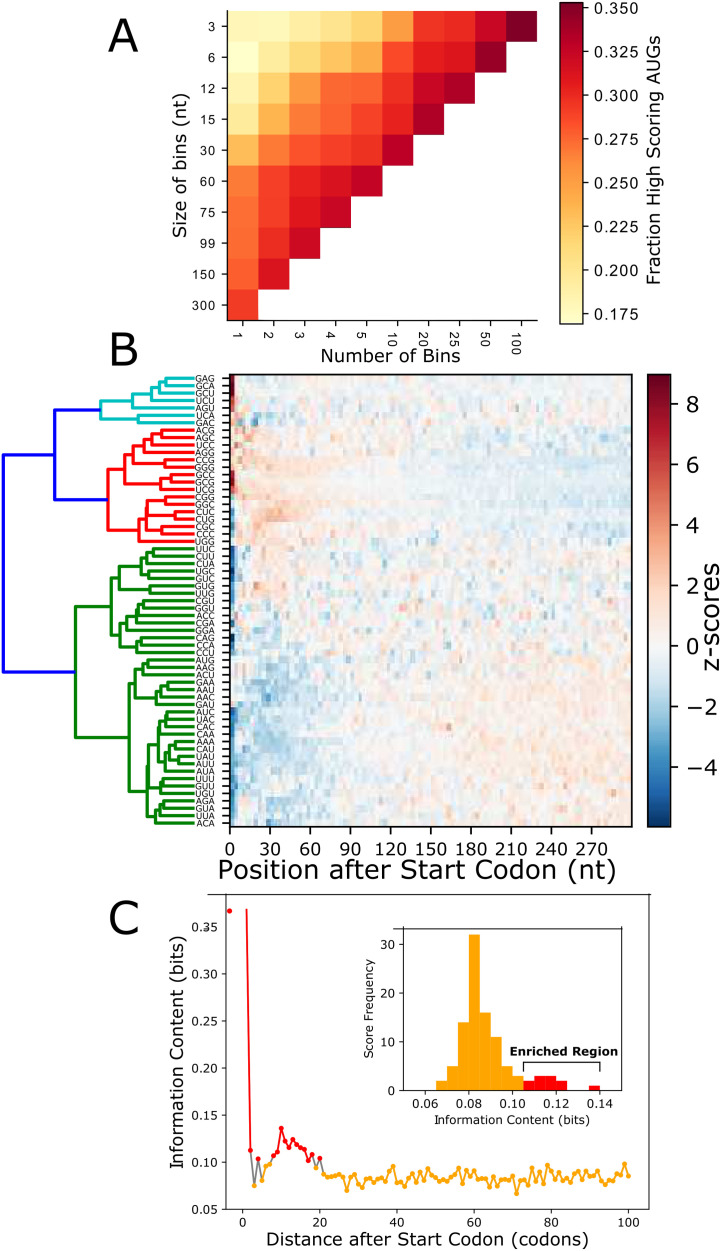
A. A heatmap demonstrating the optimal bin size and number of bins within the first 300 nt of CDS regions of the human transcriptome favors 100 3-nt bins. **B.** A codon z-score heatmap weight hierarchical clustering for the 300 nt after the start codon **C.** The information content of all transcripts, computed from position-dependent codon frequencies shows a region of enriched information content relative to the background trinucleotide frequencies. Red line segments indicate some regions of enriched information content. The inset shows a histogram of the information content for the first 100 codons with a tail of enriched information content, shown in red.

To better describe the pattern that we have observed in human transcripts, examined different ways to divide codon sequences into “bins”, i.e., windows of different lengths, and we created different versions of the POSCO matrix, which is a position-specific scoring matrix (PSSM, or position weight matrix) $W_{cb}$ to score the occurrence of a codon *c* at a positional bin *b* after a putative start codon, relative to the global frequency of the nucleotide triplet corresponding to the codon *c*. Typically, PSSMs are used to describe binding site motifs, and are indexed by nucleotide and position, but the POSCO matrix uses codons and bins. To quantify the extent to which positional trends occur for a particular range of bin lengths, we compared the score computed from the POSCO PSSM (POSCO score) at the start codon compared to other AUGs in the transcript. We used the rate at which start codons have the highest POSCO score among all other AUGs as a way to optimize parameters for our model, such as the different bin lengths and number of bins for the POSCO PSSM. We created training, validation, and test sets by subdividing the protein-coding transcripts from GENCODE using an 80:10:10 split. We assigned the POSCO score to the series of triplets that follow each AUG in the transcripts across all frames, and consider the transcript to be a “POSCO match” if the start codon has the highest POSCO score compared to other AUGs. We assessed different POSCO parameters using the match rate, defined as the percentage of POSCO match transcripts. We observed that the POSCO match rate depends on bin number and bin size, so we systematically computed the match rate for combinations of both parameters (Fig 2A). We computed the POSCO weight matrix using the training set using different combinations of bin number and size, selected an optimal set of parameters using performance on the validation set, and computed final percentages on the test set. We found that POSCO match rate was optimized using one codon per bin – in other words, the combination of 100 single-codon bins provided the best description of position-specific codon trends in human transcripts (Fig 2A). We also investigated different model lengths, corresponding to the number of bins, and found that the gradient of the match rate with respect to length is consistently positive for lengths up to around 110 bins, and reaches a minimum around this point (S7 Fig). We therefore selected a model of 300nt, or 100 3-nt bins. This model length also allows for the model to be applied to shorter ORFs compared to longer lengths. The z-score heatmap for this optimal bin size and number of bins is presented in Fig 2B. Hierarchical clustering of this heat map also corresponds to position-dependent patterns of codon GC content. We also used the POSCO probability matrix to compute the information content as a function of nucleotide position or bin (see Methods). We observed enriched information content in the range of codons 0–20, relative to and downstream of the start codon (Fig 2C). Positions beyond that showed a small but non-zero information content. While there are many signals in this region that could be related to this pattern, including the translational ramp, mRNA folding, and amino acid bias, this information content is intended to describe the strength of the POSCO probability matrix used to compute POSCO scores in way analogous to the height of sequence logos for motifs.

### POSCO match rate as a function of transcript length

**Fig 3:**
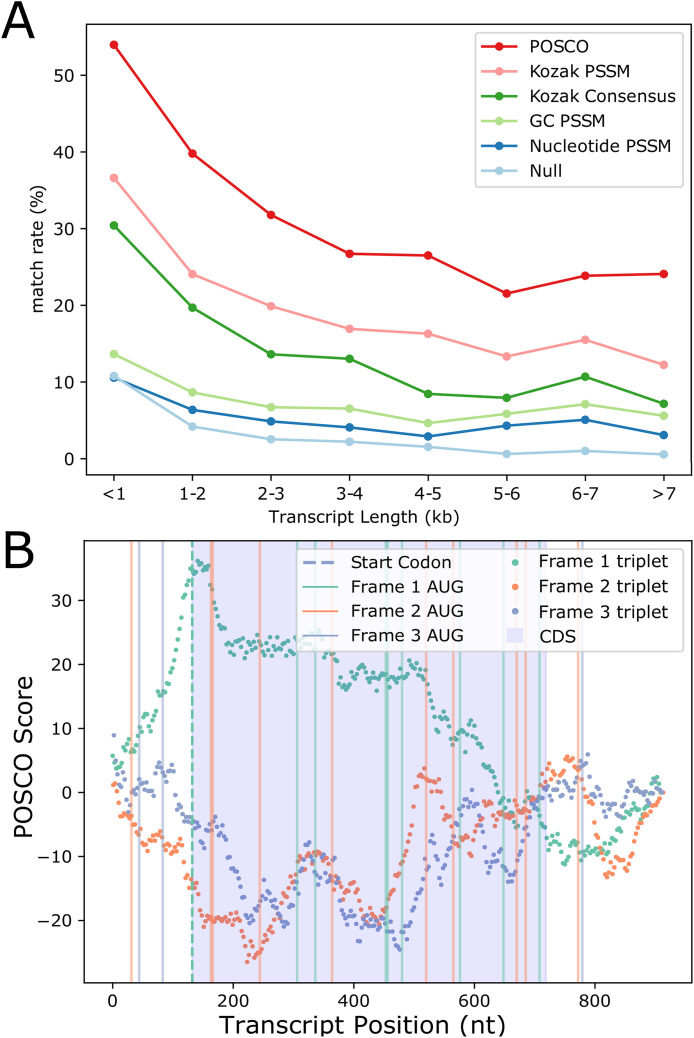
A. The start codon match rate compared for different models. The curves show the match rate for the POSCO score computed from a PSSM compared to the Kozak consensus sequence AUGGCC, a PSSM computed from the positions -10 to +2 relative to the start codon for all transcripts, a PSSM computed from the position-dependent GC content within CDS regions relative to global GC content, a PSSM computed from single-nucleotide frequencies in CDS regions relative to global nucleotide frequencies, a random selection model. **B.** The POSCO score plotted as a function of position, starting at each codon, and plotted separately for each reading frame, for an example short transcript (ENST00000662933.1, ~ 1000 nt). Blue shaded area beginning at the start codon indicates the CDS region.

We next investigated whether transcript length is correlated with the POSCO match rate in each transcript. We compared the length-dependent POSCO match rate with five other relevant sequence features. First, we compared the POSCO model to a Kozak consensus sequence model, which relies on finding within a transcript the nucleotide sequence of fewest possible mismatches to the Kozak consensus sequence [32]. We also compared the POSCO model to a position weight matrix computed from base composition in the region of the Kozak sequence, from –10 to +1 relative to the start codon. A null model that estimates the rate for the random selection of start codons amongst all AUG triplets, a position-dependent GC-content model, and a position-dependent all-nucleotide model were also used as controls. We found that the POSCO model consistently performed best among the six models (Fig 3A). Even though POSCO only considers the first 300 nt of the coding sequence, we observed improved performance with shorter transcripts. Fig 3B shows an example transcript (an isoform of MYL3 with GENCODE/Ensembl ID of ENST00000662933.1), separately plotting the POSCO score over all positions for each reading frame. This example transcript conforms to the trend observed in most transcripts of this length, with the start codon having the highest POSCO score compared to upstream and downstream AUGs.

Considering the observations made in Fig 1 demonstrating the contribution of GC content to POSCO and positional trends in amino acids, we also compared start codon identification rate to PSSM scores derived from GC content, with further comparison to amino acid occurrence (S8 Fig). Specifically, we compared PSSMs defined for position-dependent amino acid usage bias (AAPSSM), GC content in the third position of each triplet (GC3 PSSM), GC content for all positions (GC PSSM), and all nucleotides (nucleotide PSSM). In each case, the PSSMs were defined for bins, corresponding to each nucleotide in the case of GC and nucleotide PSSMs, and every third nucleotide for GC3 PSSM. We found that POSCO had a significantly higher match rate compared to the other models, suggesting that position-dependent codon trends are more descriptive of the sequence biases compared to amino acid usage and trends involving GC content alone. Finally, we compared our POSCO model to a CAI model and found that POSCO significantly outperforms CAI in its match rate, suggesting it is a better description of codon bias near the start codon (S9 Fig).

We also assessed how much of the POSCO match rate was coming from the first and second codons by removing these positions from the PSSM (S10 Fig). We found that the match rate only decreased a few percentage points when the first and second codons were removed, and performance still exceeded all other approaches.

### Comparison of POSCO between human and other species

We assessed to what extent POSCO is conserved across other mammals. We identified single copy orthologs among five primates and mouse and compared POSCO scores in the human transcriptome with the corresponding ortholog in the species of comparison (S11 Fig). We found that POSCO is largely conserved among higher apes (chimps, gorilla, and orangutans) and less so among other primates (mouse lemur and macaque). An even greater divergence was observed in POSCO scores when compared to mouse.

To further investigate the low conservation of POSCO with mouse, we first visualized the POSCO patterns in the mouse transcriptome. We found that position-dependent codon z-scores showed differences compared to humans for the large window (S12 Fig), including a longer region of enriched and depleted codons up to 1000 nt away from the start codon. When examining the first hundred codons (S13 Fig), we observed that there are several spots of sporadic enrichment at various positions. These differences aside, the same amino acids tend to be enriched near the start codon. We also compared models trained on human coding regions and applied to mouse, and models trained on mouse coding regions and applied to human (S14 Fig). The best match rate was seen for models trained on mouse and applied to mouse, with similar match rate when mouse or human POSCO are applied to human, or human POSCO applied to mouse. In general, cross-species application of POSCO PSSMs showed consistent match rate compared to same-species application. These data suggest that position-specific trends in codon occurrences are fairly well-conserved in aggregate, but may diverge for many specific genes, as seen in the single-copy orthologs between human and mouse.

### Comparison of mRNAs and lncRNAs with POSCO scores

We scored mRNAs using their annotated CDS and compared these POSCO scores to those obtained from lncRNAs by scoring the longest ORF in each lncRNA (see Methods). We found that in general, POSCO was substantially higher in mRNAs than in lncRNAs (S15 Fig). We performed a Kolmogorov-Smirnov test to evaluate the difference between the mRNA POSCO distribution and the distribution for lncRNAs, which yielded a significant difference with a *p*-value of $\text{0}.\text{7}\text{3}\text{1}\times {\text{1}\text{0}}^{-\text{7}}$. We further subdivided both the mRNAs and lncRNAs into different ranges of 1000 nt and compared their central tendencies (mean and median) with 95% confidence intervals. This was done using POSCO scores computed for start codons, non-start AUGs within the same transcripts, and the longest ORFs in lncRNAs. We found that there was a significant difference between start codons and non-start AUGs for all transcript lengths (S16 Fig).

### Functional enrichment in high-POSCO transcripts

We identified a subset of transcripts with high significance POSCO scores relative to background, which was defined as the distribution of scores observed over all non-start AUG triplets. We also divided our total transcript set into quintiles based on POSCO score (see Methods). We examined whether transcripts in these sets were enriched for biological functions or protein sequence patterns that might explain their scores.

#### Gene ontology (GO) enrichment.

**Fig 4:**
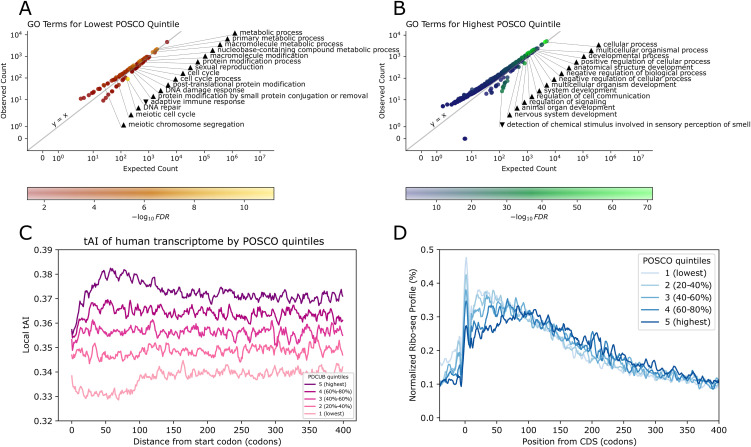
A. Scatterplot representing statistically enriched GO terms associated with transcripts in the lowest POSCO score quintile. Each dot corresponds to an enriched GO term, the x-axis position corresponds to the expected number of occurrences, the y-axis represents the observe frequency within the bottom POSCO quintile. Triangles by the labels indicate enriched (pointed up) and depleted (down) GO terms. **B.** Scatterplot representing statistically enriched GO terms associated with transcripts in the highest POSCO score quintiles, displayed similarly as **A. C.** Local tAI profiles for transcripts in quintiles defined for POSCO score. **D.** The shape of ribosomal occupancy, shown as the normalized Ribo-seq profile centered at the start codon at position 0 for quintiles defined for POSCO. Read positions are A-shifted by 15 nt, and smoothed using a Savitzky-Golay filter.

We investigated whether the transcripts in our high-significance and low-significance POSCO transcript sets, as well as in the various POSCO quintiles (see methods), were enriched or depleted for biological functions. A table of significant Gene Ontology (GO) terms is provided in S1 Table. A similar analysis was performed for CAI quintiles as a control (also in S1 Table, in different sheets). Enrichment of significant GO terms associated with transcripts in the bottom POSCO quintile is visualized in a scatterplot (Fig 4A). Similarly, GO terms that are enriched in the top quintile are shown in Fig 4B.

**Growth and development.** Among the highest-POSCO transcripts, we observed a statistical enrichment of most developmental processes, with a corresponding depletion of these same processes among the lowest-POSCO transcripts. Examples include generation, development, differentiation, formation, and morphogenesis of the sensory, nervous, endocrine, gastrointestinal, circulatory, and musculoskeletal systems, as well as of various organs, glands, tissues, cells, and cellular components. Terms with the greatest enrichment were myotube cell development, cell development, and muscle structure development, and tissue development (>1.3x expected). In these examples, between 57% to 78% of the genes with this annotation had significant POSCO scores.

**Immune response and defense.** GO terms pertaining to immune response are significantly depleted among high-POSCO transcripts and enriched among mid- and low-POSCO transcripts. We found that “adaptive immune response” was the most statistically significant GO term in the analysis of both the significant POSCO set and the top POSCO quintile and was strongly depleted in both cases.

**Stimulus and sensory perception.** Significant GO terms related to chemical stimulus *response* and *regulation* are all *enriched* among high-POSCO transcripts. By contrast, significant GO terms pertaining to chemical stimulus *detection* and *perception* are *depleted* among the high-POSCO transcripts. The enriched terms also include response to abiotic, environmental, and endogenous chemical stimulus. Response and perception of pain, light, sound, and mechanical stimulus was also enriched in high POSCO transcripts.A complete list of all POSCO scores is provided as S2 Table.

#### Comparisons between POSCO and signal peptides.

To investigate whether our results reflect the presence of nucleotide sequences coding for signal peptides, we used the signal-peptide prediction tools PrediSi [33] and SignalP [34] to predict which transcripts are likely to contain signal peptides. We performed a statistical test comparing the number of predicted signal peptides in the high-significance POSCO transcripts to a background predicted rate for a complement set of all protein-coding GENCODE transcripts not contained within the significant POSCO set. We observe that 15–24% of proteins encoded by high-POSCO transcripts are predicted to contain signal peptides, amounting to a 1–4% increase in predicted signal peptides among high-significance POSCO transcripts relative to all other protein-coding transcripts in GENCODE (S17 Fig). We tested this difference via binomial test and found the increase to be statistically significant, with a p-value of $<{\text{1}\text{0}}^{-\text{2}\text{3}}$. A Venn diagram of transcripts predicted by each approach to have a signal peptide, compared to transcripts with a high significance POSCO score, is shown in S18 Fig. Analysis of individual POSCO quintiles showed a rough correlation between signal peptide density and quintile number for PrediSi and SignalP alike. In both cases, we found the bottom quintile to have the lowest predicted density, with the third quintile being near the global average density and the fourth quintile having the highest predicted density (S19 Fig).

We also compared the statistically significant GO terms associated with significant POSCO score transcripts, both enriched and depleted, to GO terms that are enriched or depleted in significant POSCO transcripts that do not encode signal peptides (S20 Fig). We found that 435 (38%) of GO terms that are statistically enriched in significant POSCO transcripts were also enriched in the subset that do not encode signal peptides. We also found that 27 (56%) of GO terms that were significantly depleted in significant POSCO transcripts were also depleted in the subset that do not encode signal peptides. We included the GO terms that are statistically associated with significant POSCO transcripts both with and without associations with signal peptides in S3 Table. Notable enriched GO terms discussed are still enriched with signal peptides filtered out, such as “cell development”, “tissue development”, and “brain development”. Meanwhile, the notable depleted GO terms are still depleted, such as “immune response”, and “sensory perception of smell”. In fact, GO terms related to immune response and perception of smell are enriched in signal peptide containing genes, rather than depleted.

Furthermore, we visualized the codon z-score heatmap for transcripts that do not encode signal peptides (S21 Fig). We found that leucine codons in the region of 15–50nt from the start codon were removed when signal peptides were removed, which is consistent with the fact that over 80% leucine motifs are found in signal peptides [35], otherwise, apart from a few alterations, the z-score heatmap remains unchanged. We also assessed the match rate for POSCO computed from transcripts in the training set that lack signal peptides and applied to the test set and saw only a slight reduction in the match rate, and still much higher than other methods such as AAPSSM (S22 Fig).

### Comparisons between POSCO and significant AAPSSM transcripts

We also did an analysis of transcripts with a significant score for the AAPSSM compared to background. Notably, we found that the range of scores for the AAPSSM was narrower than the POSCO distribution (S23 Fig compared to S16 Fig). This is expected because many enriched and depleted codons (e.g., for valine) are canceled out at the amino acid level, and the amino acid level does not differentiate between GC-rich codons, particularly in the third position compared to POSCO. The greater variability among codons frequencies even for the same amino acid results in a wider range of POSCO scores compared to AAPSSM scores. The overlap between the start codon and background distributions for the AAPSSM is large, and as a result, fewer transcripts are statistically significant over background at an FDR of 0.05. We compared the GO terms for high POSCO transcripts and for high POSCO transcripts with high AAPSSM scoring transcripts removed and found that 84% of the enriched and 69% of depleted GO terms were still significant when AAPSSM transcripts are removed (S24 Fig and S4 Table). Again, notable enriched GO terms persist after removal of high AAPSSM genes, including “cell development”, “tissue development”, and “brain development”, as well as depleted GO terms such as “immune response”, and “sensory perception of smell”.

### Comparisons between POSCO, tAI, and CAI

We next sought to contextualize POSCO against standard measures of translational efficiency, including previous methods used to discover the translation initiation ramp [16]. As shown by Tuller and others, the translational ramp corresponds to and is distinctly marked by a rapid increase in localized tAI values near the beginning of a transcript. Specifically, this ramp comprises “relatively low-efficiency codons for about the first 30–50 positions” at translation start and is “followed by a plateau with ∼5%–10% higher translation efficiency on a genome average” [16]. Using empirical tAI values taken from Tuller *et al* (see Methods), we created a metagene plot of tAI against CDS position for the first 300 codons of the coding transcriptome and confirmed the presence of the translational ramp seen by Tuller *et al*, including the shape behavior of the ramp relative to the mean tAI across 100 randomizations of the coding sequence (S25 Fig).

We repeated this process with the transcriptome split into quintiles according to POSCO scores, and found a clear, nonoverlapping spectrum of ascending average tAI values as we move from the lowest-POSCO transcripts to the highest-POSCO transcripts (Fig 4C). This POSCO-defined spectrum exhibits differently shaped local tAI curves. The fifth quintile (top quintile, highest POSCO score) has the highest initial tAI values, the steepest initial increase in average tAI, and the highest downstream steady-state tAI values. Together, these features comprise a “translational highway”, which has higher translational efficiency than other quintiles at all points in the curve. Lower quintiles have diminishing ramp size until the second quintile, which is flat with no ramp. The first quintile (bottom quintile, lowest POSCO score) exhibits an unexpected shape in the tAI curve, with tAI descending quickly from codons 0–30 before flattening out until codon 100, whereupon we observe a short ramp that is delayed relative to those of the higher POSCO quintiles. Because the low-tAI region observed for the lowest POSCO quintile is much lower and more prolonged (100 codons compared to 30–50 codons for a “slow ramp”) than observed for the transcriptome average, it can be described as a “translational valley” in direct analogy to the well-known translational ramp [16].

We next investigated CAI scores divided into quintiles analogous to the previously described POSCO quintiles, using both a global CAI (computed over the entire coding sequence) and a regional CAI (computed over a limited region of the coding sequence, in this case the first 100 codons). Trends for tAI plotted against regional CAI showed similar but much weaker trends relative to those seen for POSCO-defined quintiles, while global CAI showed the weakest changes in tAI between the three methods (S26 Fig). Quantitative comparison of quintile-specific tAI trajectories between the three methods confirmed that for both POSCO and regional CAI the greatest change in tAI was seen in the first and fifth quintiles, corresponding respectively to a translational valley and a strong ramp, while global CAI quintiles display a steady monotonic increase from quintiles one through five (S27 Fig). Finally, we plotted tAI computed from the first 100 codons against POSCO score for the coding transcriptome and found significant correlation between the two parameters ($R^{\text{2}}\approx \text{0}.\text{3},\;p=\text{0}.\text{0}$, S28 Fig).

We calculated local tAI values for CDS regions corresponding to the first and second windows of length 30 codons for all transcripts. The midpoint between these windows corresponds roughly to the reported location of the translation initiation ramp and should thus demark a boundary between lower and higher translational efficiency signals. No such boundary was obvious in our scatter plot, regardless of the POSCO of the corresponding transcripts (S29 Fig). However, we did see a gradient of POSCO quintiles across the transcript distribution, with low-POSCO transcripts concentrated at low tAI values, and high-POSCO transcripts concentrated at high tAI values.

As an alternative approach to understanding how POSCO compares to translational efficiency, we performed a similar set of analyses with regional CAI scores (S30 Fig). This revealed a trend in which transcripts with low regional CAI in the first 30 codons (the translation initiation ramp) display higher regional CAI in the immediately subsequent 30 codons (the translation steady state). Meanwhile, for transcripts with high regional CAI in the first 30 codons, we found `the opposite relationship to be true, with lower regional CAI in the second 30 codons. Plotting the transcripts by POSCO quintile further revealed that regional CAI values increase with POSCO score for both regions (0–30 codons and 30–60 codons). To cap this line of investigation, we plotted the total CAI of each protein-coding transcript against its POSCO score, and found the relationship between CAI and POSCO to be similar to that between tAI and POSCO ($R^{\text{2}}\approx \text{0}.\text{4},\;p<{\text{1}\text{0}}^{-\text{4}\text{0}}$, S31 Fig).

### Protein-to-RNA ratio and translational efficiency

Given the correlations between tAI and POSCO scores, we next investigated protein-to-RNA ratios for mRNAs and compared to POSCO and other metrics. We computed the log2 ratio of the average protein level quantification from mass spec data and average normalized (FPKM) expression levels for 15 human tissues to define a protein-to-RNA ratio as a measure of translational efficiency. The distribution of these values for 1904 genes with available expression data for each tissue is shown in S32 Fig. We the examined the protein-to-RNA ratios for transcripts divided into quintiles for POSCO, tAI, and CAI (S33 Fig). We found that all scores showed a positive trend, with the highest quintile corresponding to the highest median protein-to-RNA ratio. However, we found that tAI had a higher-than-expected median value for the lowest quintile, while POSCO and CAI did not.

### Ribo-seq profiles and translational efficiency

Given the observation that higher ribosomal density is associated with lower translational speed [36], we examined whether transcripts with higher POSCO scores show distinct trends in ribosomal occupancy compared to transcripts with lower POSCO scores. Fig 4D shows the normalized ribosomal occupancy relative to the start codon for each POSCO quintile using data collected from HEK293 cells [37]. We observed a spectrum of ribosome profiles wherein the first quintile (lowest POSCO) has the highest occupancy at initiation relative to steady-state translation elongation, while the fifth quintile (highest POSCO) has the lowest occupancy at initiation relative to steady-state translation elongation. In contrast, a control experiment of ribosomal density plotted against CAI quintiles showed no such clear trend between the two scores (S34 Fig).

We investigated whether translational inhibition by chemical treatment affected these results by comparing ribo-seq profiles collected from HEK293 cells [38] with and without cycloheximide (CHX) treatment, and observed no significant difference in the ribosomal-occupancy spectrum observed across POSCO quintiles (S35 Fig). This result is consistent with prior work showing that CHX treatment does not affect Ribo-seq in mammalian cells [38]. In addition, we compared ribo-seq profiles across POSCO quintiles using ribo-seq data obtained from a different cell line (HeLa cells) [39], and found no significant difference (S36 Fig).

**Fig 5:**
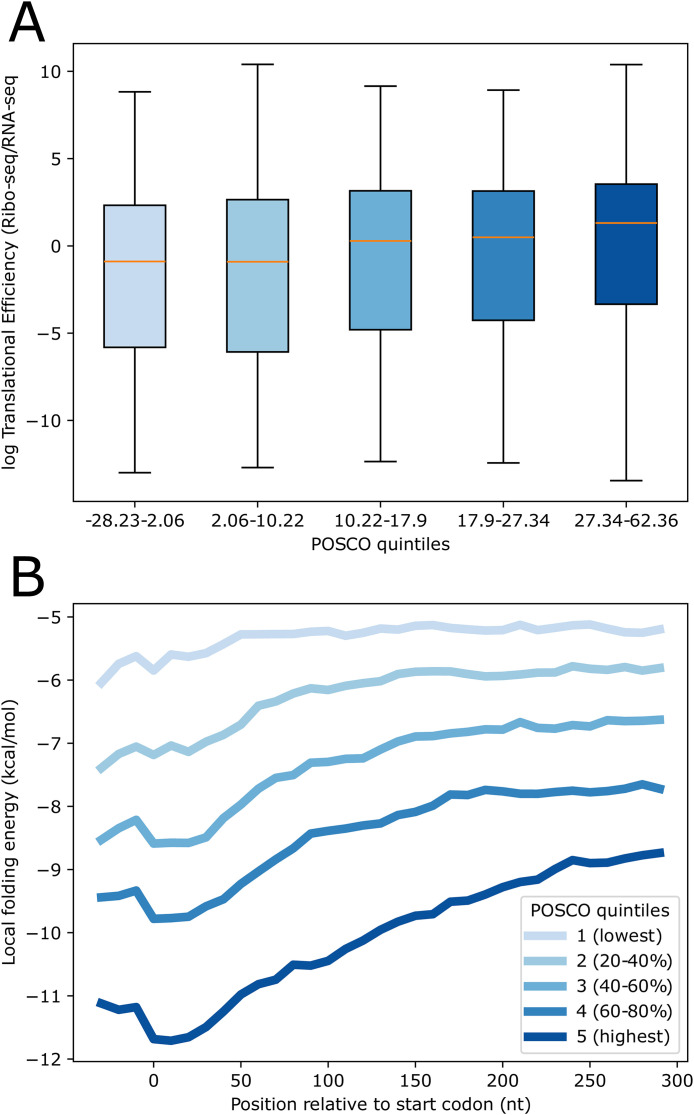
A. Box plot comparing the Translational Efficiency (TE) defined as the log2-transformed ratio of DESeq2-normalized Ribo-seq and RNA-seq data for the each POSCO quintile. **B.** Plot of the average local folding energy of 40-nt windows relative to the start codon for each POSCO quintile. Curves are computed from 40-nt windows staggered by 10nt. Minimum free energies of each window is computed with RNAfold with default parameters.

We next compared POSCO was with translational efficiency (TE), defined as the ratio of Ribo-seq reads to RNA-seq reads after RPKM normalization, for the different quintiles. The advantage of Ribo-seq compared to protein levels is that it covers more distinct transcripts, allowing for a more comprehensive analysis. We found there to be a positive association between POSCO and TE, comparable to CAI and tAI quintiles, demonstrating a similar correlation with translational efficiency for POSCO compared to the other scores (Figs 5A and S37). However, similarly to protein-to-RNA ratio, we found that tAI scores had larger than expected TE values for the lowest quintile.

### mRNA folding and high POSCO transcripts

Given previous observations on mRNA folding near the start codon regulating various aspects of translation, we also computed local folding energy, defined as the minimum free energy (MFE) of 40-nt windows starting at -20 from the start codon up to 290 from the start codon (Fig 5B), as has been done previously [23,40]. We found there is a spectrum of folding energy curves, with the highest POSCO scores corresponding to a local minimum in folding energies (stronger folding) immediately after the start codon for the first 60 nt, or 20 codons. The lowest POSCO scores correspond to almost uniform folding energies, with weak folding near the start codon.

### Logistic regression model of all scores

**Fig 6:**
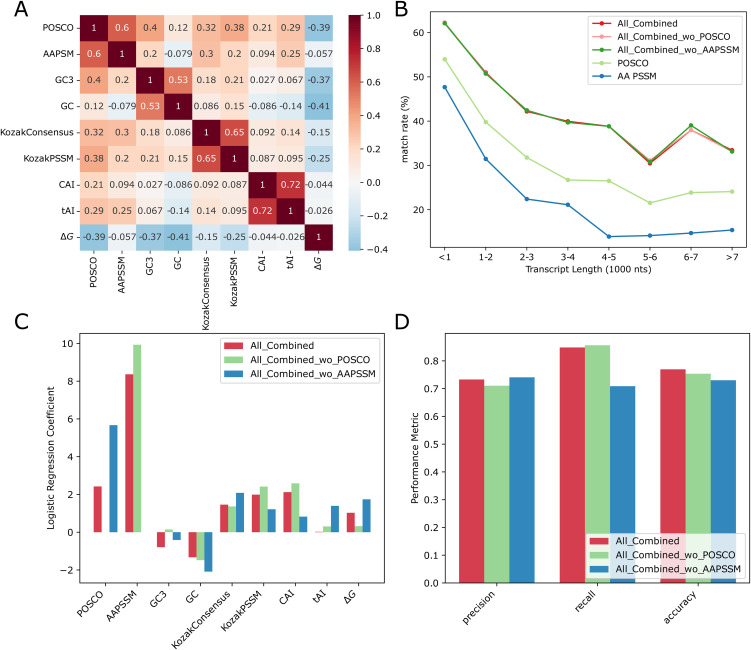
A. A correlation matrix of all scores used as features in the logistic regression analysis. **B.** Start codon match rate for a logistic regression model trained on the validation set and evaluated on the test set comparing POSCO and AAPSM scores with logistic regression models trained on all scores, all scores without POSCO, and all scores without AAPSSM **C.** Learned coefficients (weights) for a logistic regression model trained on all scores, all scores without POSCO, and all scores without AAPSSM from the validation set. **D.** Precision, recall, and accuracy on the test set for the same logistic regression model trained on all scores, all scores without POSCO, and all scores without AAPSSM on the validation set.

Finally, we also trained a logistic regression model on all the evaluated models, including POSCO, AAPSSM, GC3, GC PSSM, Kozak consensus, Kozak PSSM, CAI, tAI, and ΔG of the RNA folding of the 40-nt window 10 nt downstream of the putative start codon, which was the largest difference observed (Fig 6). First, we examined the pair-wise correlation between all scores (Fig 6A). We found that the most correlated scores were CAI and tAI, Kozak sequence and Kozak PSSM, and POSCO and AAPSSM. The POSCO score was most correlated with AAPSSM, followed by GC3, ΔG, and Kozak PSSM. Notably, ΔG showed a negative correlation since the folding energy is more negative (more stable) for stronger POSCO scores. We found that a logistic regression model composed of all features performed the best overall but observed very similar start codon match rates when removing either POSCO or AAPSSM scores, which is consistent with the correlation between these features (Fig 6B). Despite AAPSSM showing a lower match rate than POSCO as a stand-alone predictor, it does have a higher coefficient in the combined logistic regression model, which shows position-specific amino acid patterns are strongly associated with start codons. However, with the same approach leaving the AAPSSM out, also shows almost the same match rate with higher weights learned for POSCO. Since amino acids are encoded by proteins, the POSCO score provides sufficient information in the absence of the AAPSSM score. Surprisingly, GC3 was down weighted in the combined model, suggesting that only specific GC3 codons are relevant features. When observing the coefficients for each score in the max-min scaled scores using a logistic-regesion model, we found that changes in the coefficients of different features when POSCO was removed was also consistent with the correlations observed (Fig 6C). For example, when removing POSCO scores from the model we saw a compensatory increase in the coefficients for GC3 and AAPSSM. This change is consistent with the correlation between POSCO and these features, and the fact that POSCO incorporates information about amino acid bias as well as the bias toward higher GC3 codons early in the CDS. Performance for a balanced prediction task with equal number of start codons and non-start AUGs including uAUGs showed the highest accuracy with the combined model of all features (Fig 6D).

## Discussion

We observe position-dependent trends when visualizing aggregate codon usage across all protein-coding regions in the human transcriptome. We have defined a score that, unlike measures of CUB, normalizes position-dependent codon frequencies relative to the global frequency rather than synonymous codons, and demonstrate that adherence to these trends is a defining feature of many start codons. This effect was dependent on the length of the transcript, with shorter transcripts having greater POSCO match rate. We hypothesize that this is in part due to longer transcripts having more AUGs, which increases opportunities for false positives. Furthermore, shorter transcripts may require a stronger POSCO signal to better distinguish them from noncoding RNAs. We also observe a substantial difference in ribosomal occupancy near the start codon for higher-POSCO transcripts compared to those with lower POSCO, suggesting a greater ribosomal speed around the start codon. At the same time, high POSCO transcripts contain codons for highly available tRNAs (high tAI) at the beginning of the coding sequence, and greater translational efficiency overall. The low energy, stable mRNA folding immediately after the start codon for high POSCO transcripts is consistent with the hypothesis that these structures could halt scanning by the pre-initiation complex, thereby improving identification of the start codon [25–28], and prevent traffic jams near the beginning by efficiently regulating ribosomal movement [30,29]. These observations support the hypothesis that human translational machinery, including ribosomes and translation initiation factors, rely broadly upon positionally dependent sequence patterns to direct and regulate multiple aspects of translation, a fact that has been previously observed, but that POSCO encapsulates into a single score.

As discussed in previous studies in yeast [22], we tested whether our observed POSCO pattern could be explained by signal peptides and found a 1–4% enrichment of signal peptides in proteins encoded by high-significance POSCO transcripts compared to the transcriptome-wide average (S12 and S13 Figs). Sorting predictions by POSCO quintile revealed a rough correlation between POSCO quintile and predicted signal peptide density, but even the most overrepresented quintile showed <5% enrichment in signal peptides relative to the global background (S14 Fig). Interestingly, despite this correlation, both SignalP and PrediSi predict a maximum overrepresentation of signal peptides in the fourth rather than fifth POSCO quintile. Removal of signal-peptide-encoding transcripts from the significant POSCO set did not significantly alter the start codon match rate or change the major functional categories that are enriched and depleted, and only removed some enriched leucine codons from the codon z-score heatmap. In fact, many depleted GO terms in high POSCO transcripts, such as immune response related terms were enriched in signal peptide encoding transcripts. Overall, low predicted enrichment of signal peptides in all POSCO quintiles suggests that while sequence features coding for signal peptides may contribute slightly to POSCO, they are not its driving mechanism. It is also worth noting that while signal peptides are defined by an amino acid sequence, POSCO describes position-dependent patterns at the nucleotide level in addition to the triplet level and thus may capture translationally important patterns that position-dependent amino acid signals alone cannot fully resolve, such as mRNA folding.

Previous research has shown that GC-rich codons have greater translational efficiency [37]. We observe that the pattern of POSCO across transcripts shows enrichment of GC content in codons at the beginning of coding regions relative to downstream segments. Simultaneously, we observe lower ribosomal occupancy over the region in which POSCO is calculated (1–300 nt downstream of the start codon) particularly for high-POSCO transcripts. Therefore, we speculate that observed POSCO patterns may lead to greater efficiency of translation initiation and possibly also translation elongation. Selection for GC-rich, translationally efficient coding regions may encourage the development of POSCO patterns, or vice versa. However, the fact that POSCO outperforms GC content in the identification of start codons suggests that GC content is not the whole story, and higher-order sequence patterns may be at play.

Our analysis of a combined model found that POSCO was correlated with with amino acid bias, which is perhaps expected because codons encode amino acids. We also found that POSCO was much more correlated with GC3 and GC content, which confirmed our expectation that POSCO incorporates information about amino acid bias and also GC content, because it favors specific GC3 codons to occur early in the CDS. The combined model showed the highest accuracy when evaluated on a balanced test set, and the highest match rate of any other single-feature model, suggesting that start codons are decorated by a set of features. Interestingly, we found that when we remove POSCO as a feature of the logisitic regression model, the learned coefficients of the different scores changed to compensate for the missing information. For example, we saw that the weight for GC3 went from negative to positive when POSCO was removed, suggesting that AAPSSM and enhanced GC3, along with the other features, can make up for the missing POSCO score.

Tuller and others have examined the features of translational ramps in terms of complete transcriptomes or groups of transcripts based on ribosomal occupancy and other groupings in bacteria and yeast [29]. Similarly, we have investigated individual subgroups of human transcripts and found that there is a spectrum of tAI trajectories that are stratified by POSCO. Specifically, we observed that POSCO defines a spectrum of translational efficiency as seen in both ribosome occupancy, local tAI trajectories, mRNA folding, and translational efficiency. Various observations have been made about the presence of a translational ramp in mammals, with some reporting no ramp [41], others reporting a distinct ramp [16], and others reporting varying strengths of ramps based on other quantities such as ribosome density [29]. We find that both the height and slope of the translational ramp correlate with the POSCO score, with the highest-POSCO transcripts having both a strong initial increase in and steady-state average tAI, intermediate POSCO transcripts having a weaker ramp or no ramp, and the lowest-POSCO transcripts exhibiting an initial “translational valley” to an extent beyond what has been described before in terms of length and magnitude. We find that high-POSCO transcripts exhibit a rapid increase in tAI values near the beginning of a transcript as discussed previously in this manuscript and elsewhere [16,42], and an overall increased ribosomal occupancy in the early regions of transcripts relative to the post-ramp regions of those same transcripts. Among these transcripts, the highest POSCO quintile corresponds to a strong and increasing translational efficiency that can be described as a “translational highway”.

In contrast, tAI trajectories plotted against global CAI quintiles show a much weaker initial upward trend and no valley (S18A Fig). When we alter the CAI calculation to only include the first 100 codons of each transcript (the same window in which POSCO is calculated) the altered trajectories display a much weaker valley (more of a delayed ramp) and an initial ramp profile that begins to more closely (but still weakly) resemble POSCO, while downstream regions display poor inter-quintile separation compared to their POSCO and global CAI equivalents (S18B Fig). The low start codon match rate of CAI computed from the first 100 codons is further evidence of the limited ability of CAI to capture the dynamic patterns of codon usage at the start of coding regions, potentially indicating that CAI captures other features of translational control that are distinct from those captured by POSCO. All of this indicates that POSCO dramatically improves on CAI in its ability to detect important early regions of variable translation efficiency, while retaining the ability of CAI to clearly identify stable downstream regimes of translational efficiency – a balance which regional CAI fails to achieve.

Upon quantifying the absolute growth of these tAI trajectories for POSCO, CAI, and regional CAI quintiles, we see that distinct trends occur for each measure of coding potential (S19 Fig). Transcripts in the highest POSCO quintile show the highest rate of growth in localized tAI, while the second-highest growth rate occurs for the lowest quintile, corresponding to emergence from the translational valley. By contrast, for standard CAI we see a monotonic increase in the rate of growth of localized tAI across all quintiles, including no translational valley for the lowest CAI quintile. This is further evidence that POSCO captures important information regarding translational efficiency that is not captured by standard applications of CAI and tAI alone.

We also observe that POSCO quintiles stratify transcripts by the local folding energies around the start codon, with high POSCO transcripts having the lowest, most stable folds. This is in contrast to transcriptome-wide observations in bacteria, but more similar to observations in yeast [29]. POSCO scores favorably a subset of 5’ enriched codons with higher GC-content in the third position early on, which would facilitate stronger base pairing interactions in this region. Previous studies have hypothesized that local folding downstream of the start codon may help translation by halting scanning by the pre-initiation complex to help remain near the start codon, preventing other folding directly over the start codon, and preventing ribosomal traffic near the beginning of the ORF [29,30]. However, in contrast to previous studies of diverse species observe that generally observe weaker mRNA folding immediately after the start codon and stronger elsewhere, we observe that high POSCO transcripts have the strongest folding energies immediately after the start codon [23,40]. Given the lower ribosomal occupancy around the start codon observed for high-POSCO transcripts, it would suggest a faster moving ribosome. Reduced traffic jams due to the local mRNA folding in combination with the increased use of high-availability tRNAs, may lead to efficient ribosomal movement, and overall faster speed in this region, which may correlate the greater translational output observed in both protein-to-RNA ratios and translational efficiency. The POSCO-defined quintiles help to delineate a spectrum of effects related to mRNA folding that may reveal patterns that are not seen in transcriptome-wide averages.

Importantly, the POSCO-defined transcript subpopulations correspond to distinct transcript functions (S1 Table, POSCO sheets). In our analysis of GO terms, we observe that functions involved in growth and development at the system, organ, tissue, cellular, and subcellular levels are highly enriched among high-POSCO transcripts. Among the lowest quintile of POSCO transcripts, many such terms are depleted. This could be explained by the fact that developmental pathways are part of precise regulatory functions that require exquisite tuning of translational control [43,44] and precise timing [45,46]. In this case, POSCO may serve as a mechanism to enhance translational efficiency while minimizing ribosomal collisions and interruptions within regulatory cascades. This may be true particularly at the translation initiation step, thereby accounting for the pronounced translational highway found among high-POSCO transcripts and the enhanced mRNA folding stability, which may help the pre-initiation complex pause scanning near the start codon.

We observe that transcripts related to immune response, as well as those governing detection and perception of chemical stimuli, are depleted among high-POSCO transcripts. Furthermore, response to environmental, xenobiotic, and endogenous stimulus is enriched, as well as response and perception of pain, sounds, light and mechanical stimulus, which also require efficient and time-sensitive activation.

Immune response and chemical detection pathways have in common high genetic copy number and sequence variation [47]. Moreover, there is an abundance of pseudogenes observed for human odorant receptors and other genes responsible for *detection* and *perception* of chemical stimuli [48], as well as immune genes [49,50]. These observations may suggest a lower selective pressure on individual protein-coding genes due to the robustness of these pathways conferred by high copy number and a need to constantly adapt to changing chemical [51] and pathogen [52–54] environments. We posit that genes within these pathways might sample a broader range of codon ordering compared to genes involved in the development of complex vital organs.

By contrast, genes tied to chemical stimulus *response* and *regulation* show a reversed trend relative to detection and perception, with enrichment among high-POSCO transcripts and depletion among low-POSCO transcripts. This trend reversal may stem from a need for precise timing among regulatory and response genes, as described for developmental genes above. Meanwhile, detection and perception genes may rely on high copy number and low translational control to account for the complex suite of chemicals that must be adapted to in human sensory environments.

Comparison of these POSCO trends to those for CAI quintiles reveals at best partial qualitative similarity for some of the overarching GO categories identified above, with several interesting and significant deviations (S1 Table, CAI sheets). Most notably, we see enrichment of immune terms among high-CAI transcripts and depletion among mid- and low-CAI transcripts – exactly opposite of the trend seen for POSCO quintiles.

Given the prominence of these trend reversals and paired with the critical role of immune response in human evolution, we postulate that POSCO and CAI play functionally distinct roles in translational regulation. For instance, it is possible that CAI governs steady-state baseline levels of background translation, with high CAI reserved for those genes that are more consistently active, while high POSCO correlates to situationally sensitive genes that require precise timing and a more well-defined translational highway. While these two categories might overlap (as in the development terms), it is also possible for them to diverge sharply (as in the immune terms). Further evidence of the separate functionality of CAI and POSCO lies in the stark divergence of stimulus and sensory trends in CAI quintiles when compared against POSCO quintiles. Additionally, we note that the emergence of so many clearly delineable GO trends from an analysis of pure sequence data is interesting in itself and worth further investigation.

Overall, these results serve as a basis for understanding the complex relationship between POSCO and other codon bias scores, including the extent to which they may be complementary or competing factors in translational efficiency. Importantly, we find that POSCO, a single score calculated with total naivety to the known rules of protein translation, reliably accomplishes the following tasks: 1. matches true start codons with a significantly higher rate than other individual scores; 2. Reveals unexpected groupings of biological functionality; 3. sorts local tAI profiles into qualitatively distinct regimes of translational efficiency; 4. sorts Ribo-seq data into linearly ascending occupancy categories; and 5. Sorts local folding energies around the start codon into a spectrum of folding stability. Further research is needed to understand whether there are additional sorting or predictive functions of these trends in human protein translation, and the extent to which these trends occur in other species.

## Materials and methods

The transcript dataset used for this study was the GENCODE Release 34 protein-coding transcript sequences FASTA [7]. We filtered out all transcripts in which the length of the labelled coding region was not a multiple of three, lacked an in-frame start codon, or lacked an in-frame stop codon. This dataset was used for all of our analyses.

### Codon frequencies relative to transcript sequence position

Codons following the start codon were counted in bins of size 60 nucleotides (20 codons) for a total of 50 bins. To keep the bin size constant, the probability of finding a given codon in a bin was calculated, and subsequently used to derive a mean and standard deviation for each codon. The z-score of a given sense codon in a given bin in Fig 1A was calculated by


$$\begin{array}{c} z_{c,b}=\;\frac{p_{c,b}-{\;\mu }_{c}}{\sigma_{c}} \end{array}$$

where $p_{c,b}$ is the probability of a codon in a given bin, $\mu_{c}$ is the mean of the codon across all bins, and $\sigma_{c}$ is the standard deviation of the codon across all bins.

### Hierarchical clustering of codons based on z-score profiles

To obtain the dendrogram of z-score profiles, a distance matrix was first calculated wherein each row and column corresponds to a single codon. A cell at row *m* and column *n* would thus correspond to the distance between the z-score profiles of codons *m* and *n.* The distance between two codons was calculated based on the sum of squares of the differences between z-scores:


$$\begin{array}{c} D_{m,n}\;=\;\sum_{b}^{\text{5}\text{0}}(z_{m,b}-z_{n,b}{)}^{\text{2}} \end{array}$$

where $z_{m,b}$and $z_{n,b}$ are respectively the z-scores of codons *m* and *n* at bin b.

The dendrogram was computed using python’s scipy.cluster.hierarchy.linkage method [55], which performs clustering given a distance matrix such as the one calculated above. The method used for calculating distance between two clusters *u* and *v* was the ‘average’ method


$$\begin{array}{c} d(u,v)=\sum_{i,j}\frac{d(u[i],v[j])}{\left|u|*|v\right|}, \end{array}$$

which describes the distance between two clusters of codon profiles over positions i and j relative to the cardinalities of clusters u and v.

### Motivation for POSCO and other models

Several scoring models were developed to assess transcript start codons, in order to determine the effect of position dependence of translational efficiency in the human transcriptome. These models were based on a weight matrix, or position-specific scoring matrix (PSSM), where the rows correspond to codons or nucleotides and the columns correspond to positions after the start codon. In general, the PSSM values were calculated as log likelihoods based on a background model.

#### POSCO PSSM.

The weights in the POSCO PSSM were calculating according to the equation


$$\begin{array}{c} W_{cb}={log}_{\text{2}}\left(\frac{f_{cb}}{p_{c}}\right) \end{array}$$

where $f_{cb}$ is the frequency of triplet c at bin b, and $p_{c}$ refers to the calculated transcriptome-wide background probability of triplet *c* over all transcripts. We defined “bins”, which are windows of a given size that could contain one or more codons. Bins b of size w (in units of triplets) with start positions $s_{b}$ for each bin, which comprise a series of bin ranges $\left\{\left[s_{\text{1}},s_{\text{1}}+w\right],\left[s_{\text{2}},s_{\text{2}}+w\right],\ldots \left[s_{N},s_{N}+w\right]\right\}$ up to N bins for each putative CDS, where each $s_{b+\text{1}}=s_{b}+w+\text{1}$. We define a putative CDS region as series of triplets after the AUG, $C=(c_{\text{1}},c_{\text{2}},\ldots ,c_{n})$. The POSCO score S(C) is computed for a given CDS according to


$$\begin{array}{c} \text{S}(\text{C})=\sum_{b=\text{1}}^{N}\sum_{i=s_{b}}^{s_{b}+w}W_{c_{i},b} \end{array}$$

with POSCO models computed at bin sizes of w= 1, 2, 3, 4, 5, 10, 20, 25, 50, and 100 triplets. After examining the percentage of transcripts in which the true start codon was the highest scoring AUG, it was determined that bins of size of 3 nucleotides (single-codon bins) was optimal.

#### Kozak consensus sequence.

The Kozak consensus sequence is defined as (gcc)gccRccAUGG, where R corresponds to A or G, and position +1 corresponds to the A in the AUG. Positions -3 (the R) and +3 (the G after the AUG) are particularly important, and upper-case indicates highly conserved bases [32]. We gave positions +3 and -3 a weight of 6, and the remaining positions a weight of 1. The score of +6 for each highly conserved position ensures that correctly matching one highly conserved position outweighs any number of less-conserved matches. Transcripts were scored based on the number of mismatches the surrounding nucleotides had with the consensus sequence, with a maximum score being 17 (no mismatches). The leading (gcc) was subtracted from this score, so that codons containing the matching sequences were rewarded, but codons without them were not penalized.

#### Calculation of Kozak PSSM.

The Kozak PSSM model was implemented in a manner similar to that for POSCO, with a few key differences. A PSSM was built by computing the frequencies $f_{b,i}$ for each nucleotide b at position i relative to the start codon across all protein-coding transcripts. The PSSM is then computed using this frequency relative to the global single-nucleotide frequencies $p_{b}$, as shown below.


$$\begin{array}{c} W_{b,i}={log}_{\text{2}}\left(\frac{f_{b,i}}{p_{b}}\right) \end{array}$$

Therefore, the Kozak PSSM differs from the POSCO PSSM because the former was based on nucleotide counts instead of codon counts. Positions in the Kozak PSSM correspond to the ten nucleotides before the start codon, which is the location of the Kozak consensus sequence. All positions in the transcript were thus scored by taking the sum of the weights of ten preceding nucleotides.

#### Calculation of GC content PSSM.

The GC content PSSM has two rows, one corresponding to ‘S’ nucleotides [G,C] and the other to ‘W’ nucleotides [A,U]. Nucleotides were counted into either of those categories in a manner similar to the nucleotide PSSM used for the Kozak weight matrix.

*Calculation of background models: Null model and theoretical model* The null model was calculated to determine the apparent extent of random chance involved in an AUG being the true start codon in a given transcript. All AUG triplets in a transcript were scored, and an AUG was randomly selected as the start codon. If the selected AUG had the highest score, then the model was considered successful for that transcript.

### Combined regression model

Results from a logistic regression model from scikit-learn presented in S11 Fig was performed by integrating all other models trained on the training set. Weights for a logistic regression model were fit using the validation set on start codons labeled 1, and non-start AUGs labeled 0. The models were ultimately applied on the test set for evaluation. All scores were max-min normalized using scikit-learn MaxMinScaler.

### Scoring of lncRNAs using the longest ORFs

For each lncRNA we identified the longest ORF, meaning the longest subsequence within the lncRNA transcript that begins with a start triplet, ends with a stop triplet, and has a length divisible by 3. We then scored the longest ORF subsequence with the same POSCO PSSM that was applied to mRNAs.

### Cross-validation methods in comparing POSCO and other models

The dataset of all valid sequences was split into three separate sets. In all cases, 80% of the data (about 55,000 transcripts) were used to obtain PSSMs of the model in question. In most cases, the remaining 20% (about 14,000 transcripts) were scored with the calculated PSSM. In the case of POSCO, where multiple bin sizes were tested, 10% of the data was used to determine the bin size that produced the highest-scoring AUG triplets, and 10% was left for scoring.

### Calculating information content of bins within a transcript

The information content IC of position i in a transcript was calculated as follows:


$$\begin{array}{c} IC(i)\;=\;H_{f}-H_{p}(i) \end{array}$$

where $H_{f}$ is the Shannon entropy of the global triplet frequencies (e.g., the background model) of the transcript, and $H_{p}(i)$ is the Shannon entropy of the position-dependent codon frequencies at position i. The background entropy is calculated as


$$\begin{array}{c} H_{f}\;=-\sum_{c}f_{c}{log}_{\text{2}}(f_{c}) \end{array}$$

where $f_{c}$ is the global frequency of triplet *c.* The product of the global frequency and the log of the global frequency are computed for all triplets and then added together. The Shannon entropy of the position-dependent codon frequencies is calculated as


$$\begin{array}{c} H_{p}(i)\;=\;-\sum_{c}p_{c,i}{\;log}_{\text{2}}(p_{c,i}) \end{array}$$

where $p_{c,i}$ is the probability of codon c at position i*.*

### Determination of high-significance AUG triplets and POSCO quintiles

Upon generating a list of transcripts and their highest-scoring AUG triplet based on the POSCO model, the p-values for each AUG score were calculated using a normal distribution with the mean and standard deviation of all non-start AUGs, with the latter representing a null model relative to which a particular AUG can be scored (see S16 Fig). All scores were sorted by *p*-value and ranked such that the lowest *p*-value would have a rank of 1, the next lowest a rank of 2, and so on. These values were filtered with a Benjamini-Hochberg multiple test correction to produce a list of statistically significant genes (α < 0.05), with corresponding *q*-values calculated according to


$$\begin{array}{c} q_{r}=\;\frac{P_{r}*N}{r} \end{array}$$

where *r* is the rank, $P_{r}$ is the r^th^-ranked p-value, and *N* is the total number of scores. All transcripts with a rank lower than the highest rank r with $q_{r}$ below the significance level were deemed statistically significant. This set was considered the high-significance POSCO set (significant POSCO set), while an equivalently sized transcript set with the highest $q_{r}$ was considered the low-significance POSCO set.

A second ranking system was also used, wherein all transcripts were divided into a set of quintiles according to POSCO score. The 20% of transcripts with the highest POSCO score comprise the top POSCO quintile and so on (see Table 1).

**Table 1:** Definition of POSCO quintiles.

POSCO quintile	percentile range (%)	quintile name
5	81-100	fifth quintile, top quintile, highest POSCO
4	61-80	fourth quintile
3	41-60	third quintile, middle quintile, moderate POSCO
2	21-40	second quintile
1	01-20	first quintile, bottom quintile, lowest POSCO

### Analyzing the contribution of signal peptides

#### Prediction of signal peptides in human proteome.

The mRNA sequences of the human transcriptome were retrieved from GENCODE, converted to protein sequence format, and fed to the online tools PrediSi [33] and SignalP-5.0 [34] for prediction of signal peptides. Both tools were accessed through their public-facing online interfaces using default program parameters. In both cases, transcripts from the significant POSCO set were separated from the remainder of the GENCODE set.

#### Statistical enrichment of signal peptides in significant POSCO set and quintiles.

Predicted signal peptide densities for the significant POSCO set and each POSCO quintile were compared to background signal peptide enrichment in two batches: one for the PrediSi scores, and another for the SignalP scores. In both cases, the significant POSCO set and quintile sets were tested for enrichment of signal peptides relative to the global set. Enrichment significance was determined via binomial test.

#### Overlap between significant POSCO and signal peptides.

Transcripts from the significant POSCO set were checked for overlap with all GENCODE protein-coding transcripts predicted to have a signal peptide. This was done for two sets of transcripts: GENCODE transcripts that scored positively for signal peptides using PrediSi, and those that scored positively for signal peptides using SignalP-5.0. All three resulting sets (significant POSCO, PrediSi GENCODE, and SignalP-5.0 GENCODE) were visualized together as a Venn diagram using matplotlib.

### Gene ontology enrichment of genes within different POSCO quintiles

ENSEMBL IDs for transcripts were analyzed for each POSCO category (high-significance, top quintile, and so on) using the GO Enrichment Analysis tool, which is part of the web-accessible Gene Ontology Resource (http://geneontology.org/) [56–58]. Tables of statistically significant GO terms were retrieved for both sets of transcripts. The tables are provided as Excel files in S1 Table. Significant GO terms were those with unexpected enrichment or depletion among a given POSCO category, upon application of a Benjamini-Hochberg multiple test correction with a false discovery rate of 0.05.

### Calculating regional CAI and local tAI

Sets of local relative adaptiveness (RA) and CAI values were calculated for all protein-coding GENCODE human transcripts. To construct a reference set for human CAI calculations, we obtained protein expression data from the Human Proteome Map [59] and identified proteins that were universally translated in 15 human tissues (adrenal gland, brain, colon, esophagus, gallbladder, heart, kidney, liver, lung, ovary, pancreas, placenta, prostate, testis, and urinary bladder). These protein expression data were compared against corresponding transcript expression data obtained from Fagerberg et al [60]. The tissue-specific values of universally expressed genes were passed through a linear fitting function to obtain optimal fit values for each gene. A y-intercept of zero was used in this function to avoid the unrealistic result of negative protein expression. Genes with a ratio of protein expression to transcript expression greater than one standard deviation above the mean of these optimal fit values were taken to constitute a high-efficiency reference set, which ultimately numbered 125 high-efficiency genes.

Our CAI calculations used standard RA weights, defined by one unique set of values for a given transcriptome and derived from total codon composition across the full lengths of every CDS:


$$\begin{array}{c} w_{c}=\frac{f_{c}}{max\left(f_{c^{\prime }}\right)} \end{array}$$

where $f_{c}$ is the frequency of codon c, $f_{c^{\prime }}$ is the frequency of codon $c^{\prime }$, and c and $c^{\prime }$ are synonymous codons for the same amino acid. From this, the regional CAI was calculated as


$$\begin{array}{c} {CAI}_{r}={\left(\prod_{k=\text{1}}^{l_{r}}w_{c_{kr}}\right)}^{\frac{\text{1}}{l_{r}}} \end{array}$$

where $l_{r}$ is the length of the transcript region, and $c_{kr}$ is the codon defined by the k^th^ triplet in that region.

To calculate local tAI, we retrieved human codon tAI weights from S1 Table of Tuller *et al.* [16] and computed a similar geometric mean for the same regions as above. Quintile tAI figures were generated by splitting GENCODE transcripts into five parts sorted by POSCO score. Unlike regional CAI, which consists of a single average value for the entire region under consideration, average local tAI was plotted for each quintile as a function of codon position using a sliding window five codons in length.

### Calculation of ribosome profiles

Ribosome profiling of HEK293 cells was downloaded from GSE accession number GSE126298 [37]. Ribosome profiling of HEK293 cells with and without CHX treatment was downloaded from GSE accession number GSE136940 [38], and for Hela cells from accession GSE102720 [39]. Ribo-seq reads were aligned to protein-coding transcripts using Bowtie [61] with the parameters “-v 2 -a -S --strata --best --norc -m 200” after trimming the adaptors with Cutadapt [62]. Read 5′ ends were shifted relative to the start of the CDS and an additional 15 nucleotides to align to the A site [63]. Normalization of each individual transcript was performed by dividing the sum of the reads at each nucleotide position by the total number of reads mapping up to 500 codons from the start codon for that transcript and converted to a percentage. The sum at each position of these normalized transcripts was divided by the total number of transcripts to create an average profile. The average curves for each quintile were smoothed using a Savitzky-Golay filter [64] with a window size of 19 and a polynomial degree of 4 using the scipy implementation [55].

Translational Efficiency (TE) is computed as the log2-transformed ratio of DESeq2-normalized Ribo-seq and RNA-seq data for the each POSCO quintile using the Ribo-seq, RNA-seq and approach described in Hia 2019 [37].

### Calculation of local folding energies

Average local folding energy of 40-nt windows at positions relative to the start codon and staggered 10nt between each point were computed for each POSCO quintile for Fig 5B. Minimum free energies of each window is computed with RNAfold, part of the ViennaFold suite with default parameters [65].

### Logistic regression model

We computed a logistic regression model using all scores defined in this project, including including POSCO, AAPSSM, GC3, GC PSSM, Kozak consensus, Kozak PSSM, CAI, tAI, and ΔG of the RNA folding of the 40-nt window 10 nt downstream of the putative start codon or AUG triplet. The model was trained on the validation set and tested on the test set. The training set was used to build PSSMs for scoring the models. We used scikit-learn implementations of logistic regression and performance metrics. We built a balanced data set to train the model and used max-min-scaling for each feature. We computed a correlation matrix of all features on the validation set using the pandas df.corr() method. We examined the coefficients of the all-feature model, as well as with POSCO and AAPSSM scores removed.

### Project code

Code for this project is available at https://github.com/hendrixlab/POSCO.

## Supporting information

S1 Fig Z-score heatmap computed from codon counts in bins of size 60 nt, for the first 3000 nt of all coding regions. Codons are grouped by amino acid and by the chemical category of the amino acid.
(PNG)

S2 Fig Z-score heatmap computed from codon counts in bins of size 3 nt, for the first 300 nt of all coding regions. Codons are grouped by amino acid and by the chemical category of the amino acid.
(PNG)

S3 Fig Scatter-boxplot comparing the distance between codons based on hierarchical clustering of the position-dependent z-score profiles versus GC nucleotide content. (PNG)

S4 Fig Curves of z-scores computed from codon occurrence frequencies, shown in clusters corresponding to GC nucleotide content. Each line corresponds to a codon’s enrichment over background for all sense codons.
(PNG)

S5 Fig Heatmap of z-scores for sequences in which codons have been shuffled among their respective synonymous codon pools. Amino acid sequences and individual codon frequencies are all preserved.
(PNG)

S6 Fig Heatmap of z-scores for sequences visualized by amino acid content. Heat map values show the z-scores of amino acid counts at a particular position relative to the global average counts and standard deviation.
(PNG)

S7 Fig Determining an optimal model length. Blue dots represent the match rate as a function of the model length in codons for POSCO applied to the test set. Light blue curve is a Savitsky-Golay smoothed curve using a window length of 11 and a polynomial order of 3. Red curve is the difference between adjacent values of the smoothed curve (discrete derivative) and dark red curve is a sliding-window average with a window of 10.
(PNG)

S8 Fig Rate of start codon identification compared for various coding sequence content models. Curves show the fraction of start codons correctly identified per transcript for the POSCO PSSM (red), AA PSSM (pink), GC3 PSSM (dark green).
(PNG)

S9 Fig Start codon match rate for POSCO (red) compared to local CAI (pink), with null model (green) for reference. (PNG)

S10 Fig Start codon match rate for POSCO (red) compared to CAI (green), with null model (pink) for reference. (PNG)

S11 Fig Comparison of POSCO for single copy orthologs in primate and mouse transcriptomes. Correlations are computed comparing the POSCO scores for human and the ortholog in the comparison transcriptome. The correlation for human is 1.0 because it is comparing with the same transcriptome.
(PNG)

S12 Fig Codon z-score heatmap for the mouse transcriptome. A larger region of significant z-scores is observed, up to 800 or 900 nt from the start codon compared to the human transcriptome S1 Fig.
(PNG)

S13 Fig A zoomed-in codon z-score heatmap for the mouse transcriptome, for three-nt (single codon) bins up to 300-nt from the start codon. Sporadic bins of enrichment are observed throughout compared to the human heatmap, shown in S2 Fig.
(PNG)

S14 Fig Start codon match rate for POSCO scores computed from human transcripts and from mouse transcripts, applied to both human and mouse transcripts. Both models are trained on length-stratified 80% of the transcriptome and tested on 10% of sequences in the test set.
(PNG)

S15 Fig Histograms comparing the POSCO score for start codons compared to non-start AUG triplets and the AUG in the longest ORF for lncRNAs. Mean and standard deviation from the non-start distribution is used to compute statistical significance of POSCO scores.
(PNG)

S16 Fig Comparison of the mean and median POSCO score for start codons in transcripts of different lengths in the test set, relative to non-start AUG triplets, in increments of 1000 nt. In addition, this also compares to the scores for the AUG in the longest ORF for lncRNAs using the same size bins.
(PNG)

S17 Fig Overlayed histograms comparing the predicted probabilities of signal peptides for significant POSCO transcripts in light blue against those of the full GENCODE set in pink. The darker blue/purple color is the overlap between two semi-transparent histograms. A. SignalP distribution. B. PrediSi distribution.
(PNG)

S18 Fig Venn diagram of coding transcripts predicted to have signal peptides according to SignalP (top left) and PrediSi (top right), compared to coding transcripts that meet the significance threshold for high POSCO score (bottom). (PNG)

S19 Fig Predicted signal peptide densities for each POSCO quintile. Magenta line indicates global predicted signal peptide density. A. SignalP predictions. B. PrediSi predictions.
(PNG)

S20 Fig Venn diagram comparing GO terms associated with significant POSCO scores with and without signal peptides according to SignalP and PrediSi. Top Venn shows statistically enriched GO terms, and bottom Venn shows statistically depleted terms.
(PNG)

S21 Fig Codon z-score heatmap for transcripts that do not encode signal peptides according to SignalP and PrediSi. (PNG)

S22 Fig Start codon match rate for POSCO scores computed with from transcripts with and without signal peptides compared to AAPSSM scores. Transcripts filtered by SignalP and PrediSi scores greater than 0.5.
(PNG)

S23 Fig A comparison of the distributions of AA PSSM scores for start codons and compared to background (non-start AUG codons). (PNG)

S24 Fig A comparison of enriched GO terms associated with significant POSCO transcripts compared to GO terms associated with significant POSCO and do not have a significant AA PSSM score. (PNG)

S25 Fig Average local tAI for first 300 codons of coding transcriptome in humans, using a sliding window of size 5 codons. A distinct ramp is seen from codons 0–50. The dashed line shows the average local tAI across 100 randomizations of the coding sequence. Dotted lines indicates three standard deviations above and below the randomized mean.
(PNG)

S26 Fig Local tAI plotted against coding sequence position according to CAI quintile. Different colors represent tAI curves for individual quintiles. Grey lines indicate the statistical upper and lower bounds of local tAI averaged over 100 randomizations of all coding sequences. A. Global CAI. B. Regional CAI.
(PNG)

S27 Fig Comparison of increase in tAI by quintile. A. Side-by-side percent increase in tAI from lowest point to highest point for each POSCO, CAI, and regional CAI quintile. B. Ratio of the tAI for fifth and first POSCO quintiles as a function of position for 5-codon windows.
(PNG)

S28 Fig Scatter plot of local tAI for the first 100 codons of all coding transcripts, plotted against the POSCO score of the same transcript. Each dot corresponds to a transcript. The dashed line is the best linear fit to the scatter data.
(PNG)

S29 Fig Regional tAI of the second vs first 90-nt (30-codon) bins for all protein-coding human transcripts, color-coded by POSCO quintile. A. All quintiles with best fit line. B. First quintile (lowest POSCO); dark blue. C. Second quintile; cyan. D. Third quintile; magenta. E. Fourth quintile; red. F. Fifth quintile (highest POSCO); yellow.
(PNG)

S30 Fig Regional CAI of the second vs first 90-nt (30-codon) bins for all protein-coding human transcripts, color-coded by POSCO quintile. A. All quintiles with best fit line. B. First quintile (lowest POSCO); dark blue. C. Second quintile; cyan. D. Third quintile; magenta. E. Fourth quintile; red. F. Fifth quintile (highest POSCO); yellow.
(PNG)

S31 Fig Scatter plot of CAI for each coding transcript plotted against the POSCO score of the same transcript. Each dot corresponds to a transcript. The dashed magenta line is the best linear fit to the scatter data.
(PNG)

S32 Fig Histogram showing the distribution of log-transformed protein-to-RNA ratios across 1904 genes. (PNG)

S33 Fig Scatter boxplots comparing log2 protein-to-RNA ratios for genes divided into quintiles for POSCO, tAI, and CAI. (PNG)

S34 Fig The shape of ribosomal occupancy collected from HEK293 cells, shown as the normalized Ribo-seq profile centered at the start codon at position 0 for each quintile defined by CAI. Read positions are A-shifted by 15-nt, and smoothed using a Savitzky-Golay filter.
(PNG)

S35 Fig Riboseq profiles collected from HEK293 cells in the absence and presence of cycloheximide (CHX). A. Without CHX. B. With CHX.
(PNG)

S36 Fig The shape of ribosomal occupancy collected from HeLa cells, shown as the normalized Ribo-seq profile centered at the start codon at position 0 for each quintile defined by CAI. Read positions are A-shifted by 15-nt, and smoothed using a Savitzky-Golay filter.
(PNG)

S37 Fig Scatterplots comparing log2 TE ratios of DESeq2-normalized ribo-seq and RNA-seq data for transcripts divided into quintiles for tAI and CAI. (PNG)

S1 Table POSCO and CAI quintiles, and associated enriched GO terms. (XLSX)

S2 Table POSCO scores for all human GENCODE transcripts. (XLSX)

S3 Table Enriched GO terms associated with significant POSCO transcripts, with and without signal peptides, the intersection, and GO terms enriched in transcripts encoding signal peptides. (XLSX)

S4 Table Enriched GO terms associated with significant POSCO transcripts compared to those with significant AAPSSM transcripts excluded, and the intersection. (XLSX)
