# Supplementary figures and images for: Position-dependent codon occurrence is associated with patterns of translational efficiency and ribosomal occupancy in the human transcriptome
Source: PLoS Comput Biol. 2026 Jul 20;22(7):e1014501. doi: 10.1371/journal.pcbi.1014501 (PMC13399515; doi:10.1371/journal.pcbi.1014501)

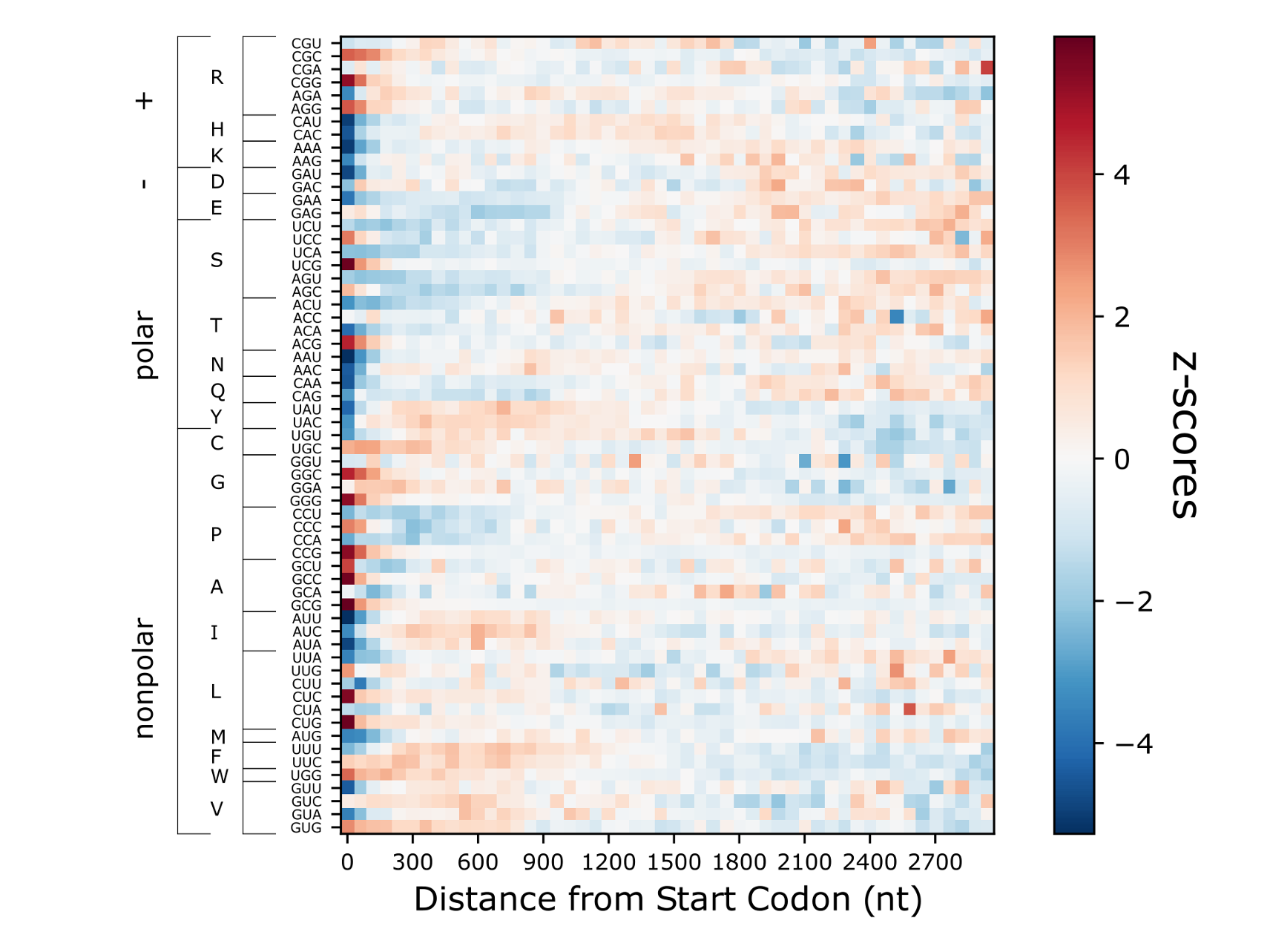

Supplement: S1 Fig — Codons are grouped by amino acid and by the chemical category of the amino acid. (PNG) [file pcbi.1014501.s001.png]

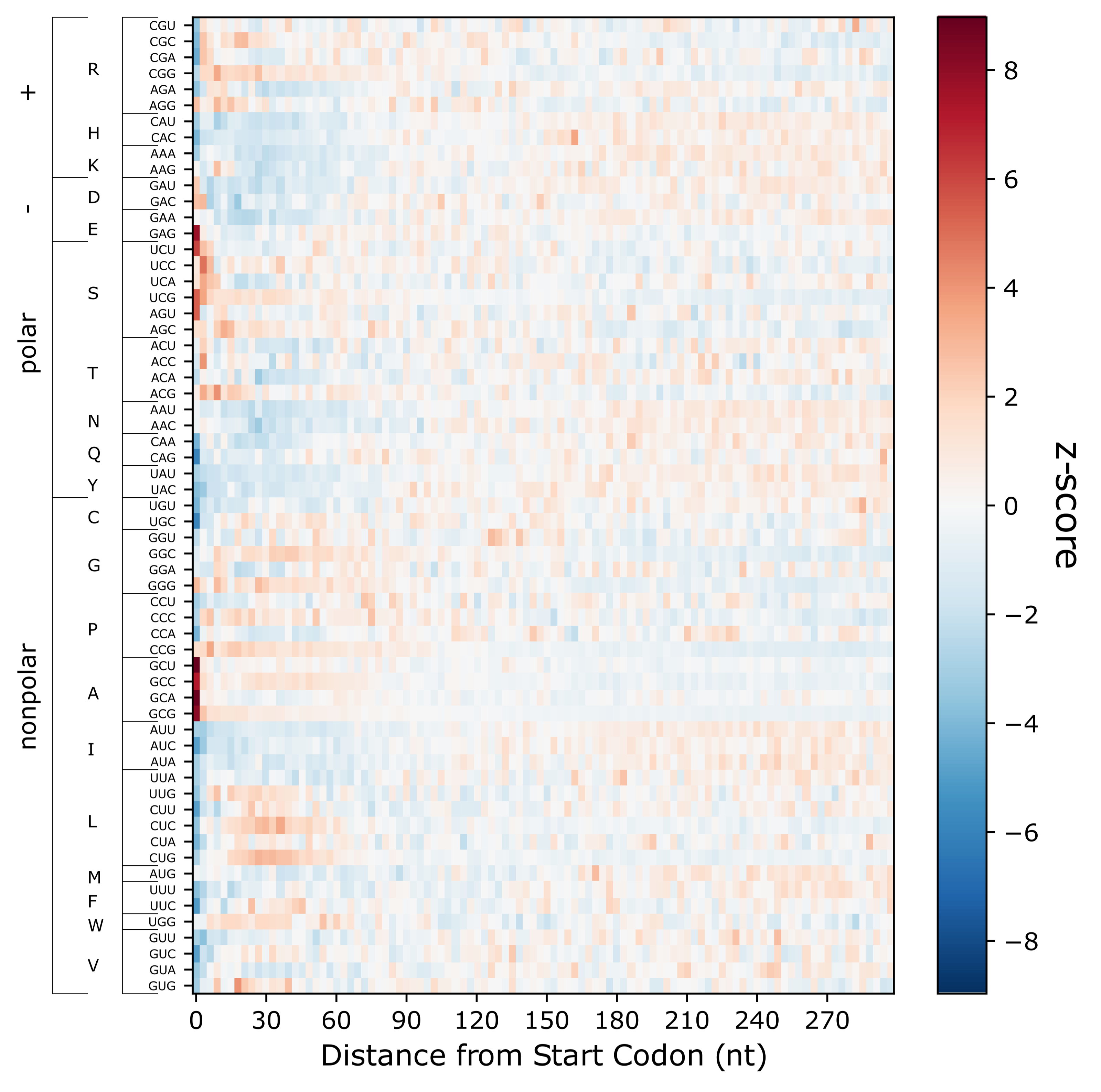

Supplement: S2 Fig — Codons are grouped by amino acid and by the chemical category of the amino acid. (PNG) [file pcbi.1014501.s002.png]

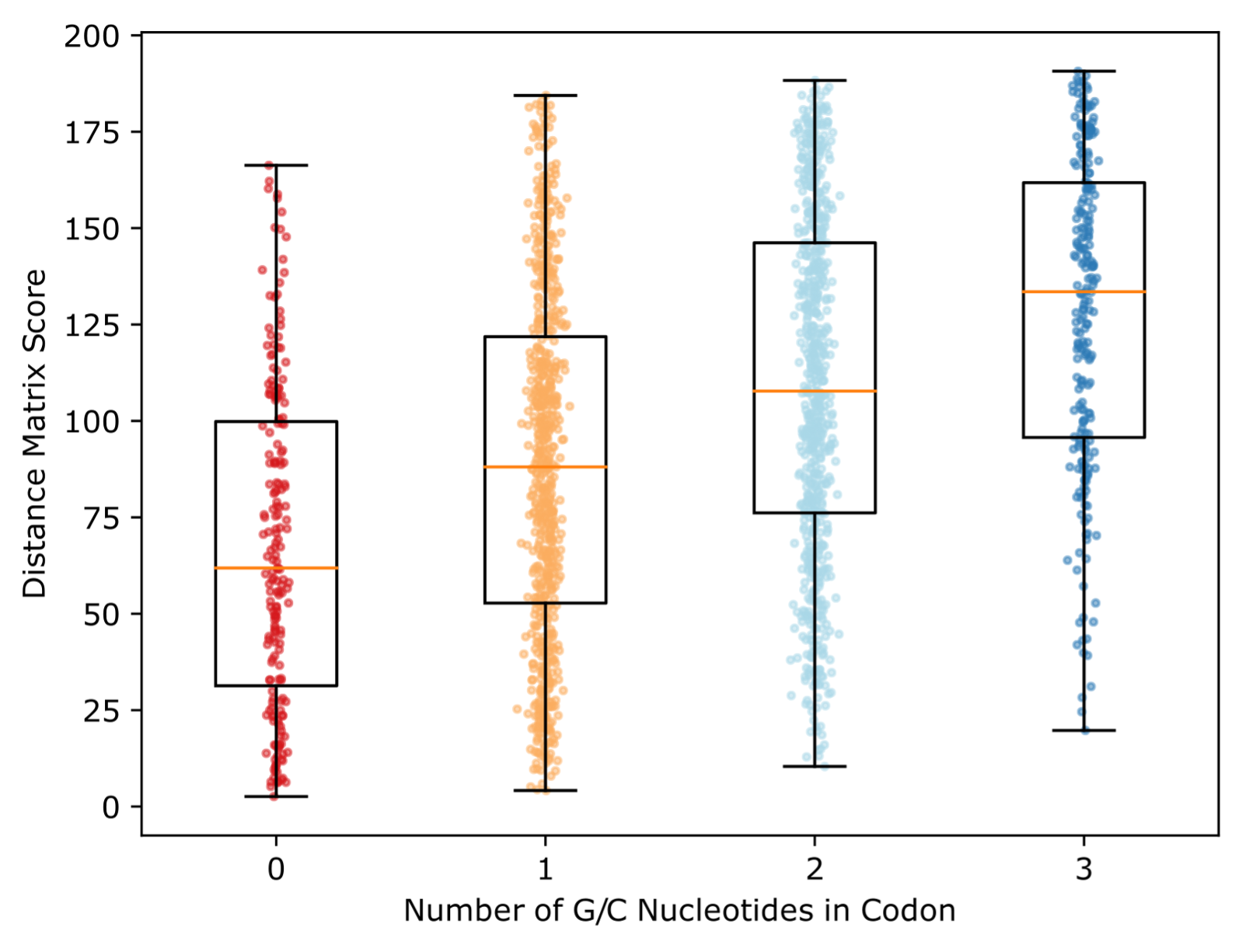

Supplement: S3 Fig — (PNG) [file pcbi.1014501.s003.png]

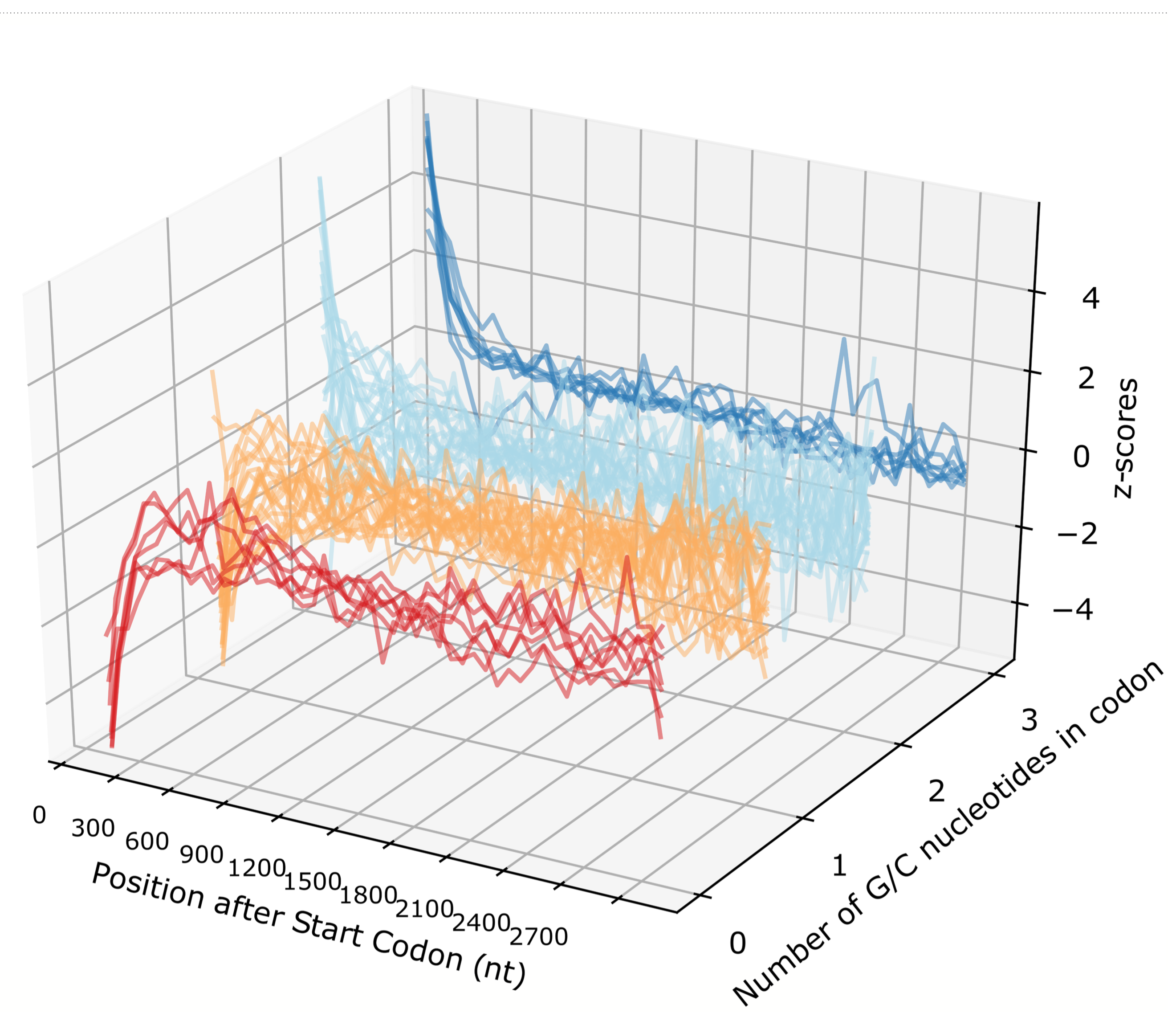

Supplement: S4 Fig — Each line corresponds to a codon’s enrichment over background for all sense codons. (PNG) [file pcbi.1014501.s004.png]

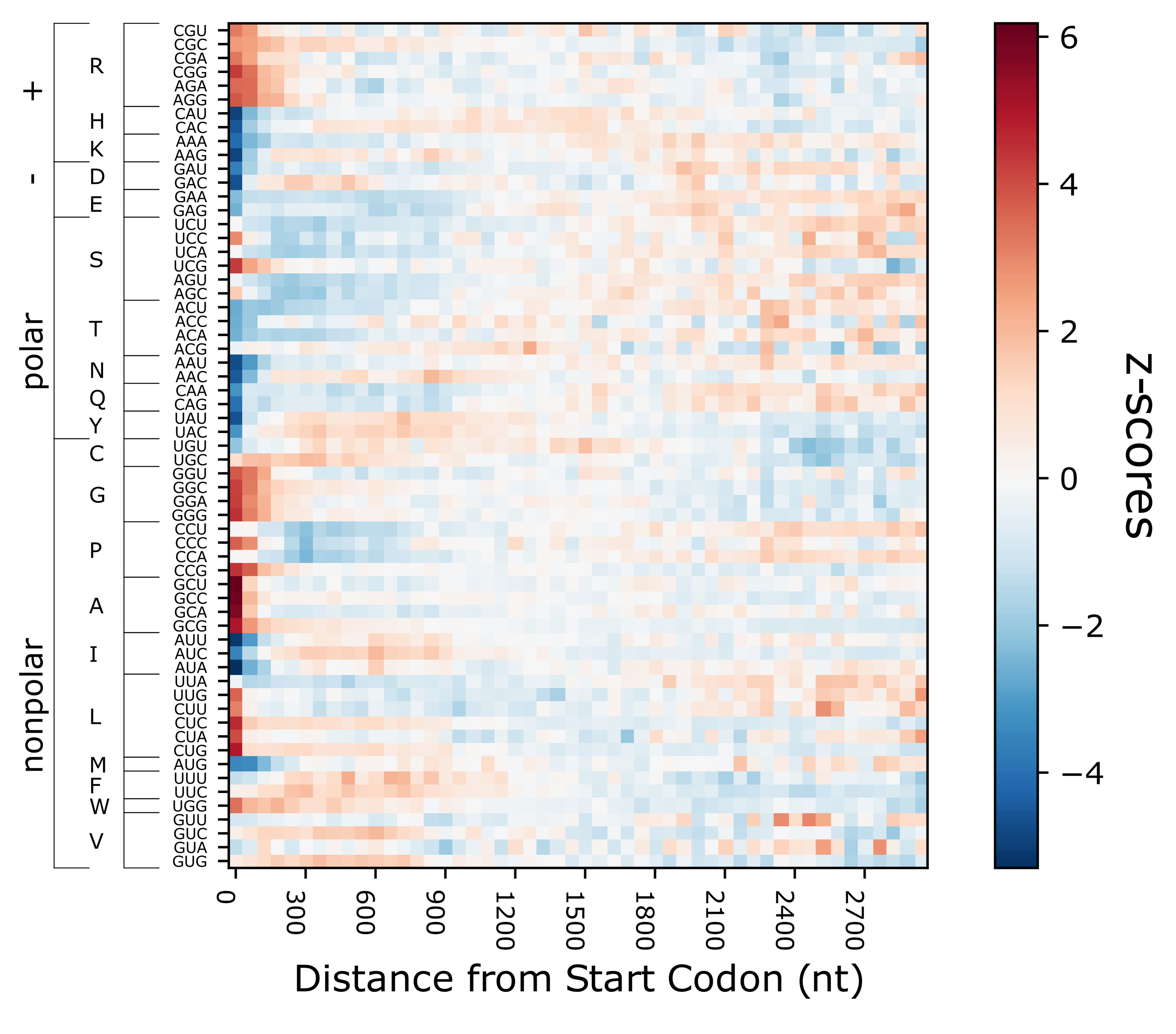

Supplement: S5 Fig — Amino acid sequences and individual codon frequencies are all preserved. (PNG) [file pcbi.1014501.s005.png]

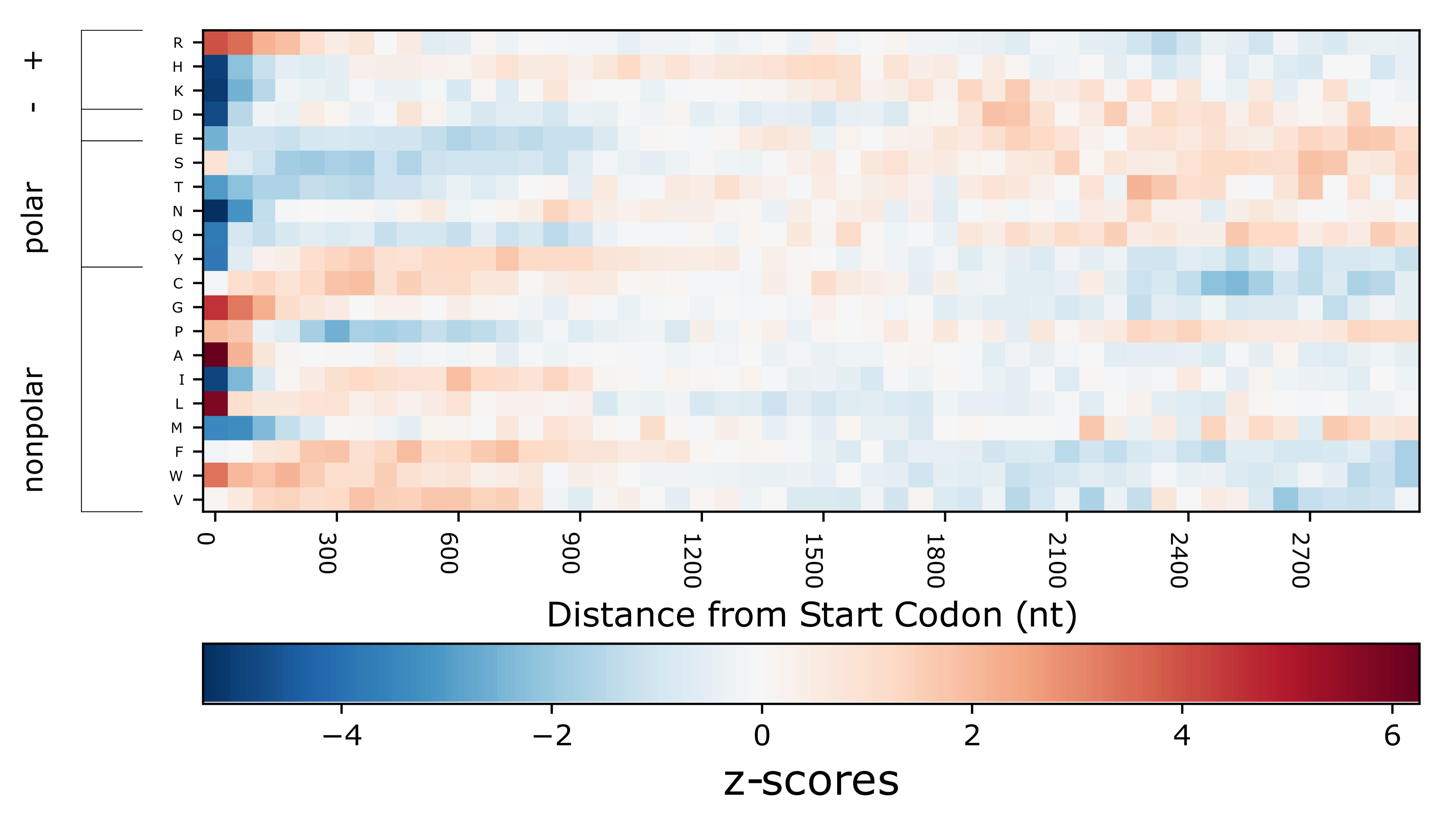

Supplement: S6 Fig — Heat map values show the z-scores of amino acid counts at a particular position relative to the global average counts and standard deviation. (PNG) [file pcbi.1014501.s006.png]

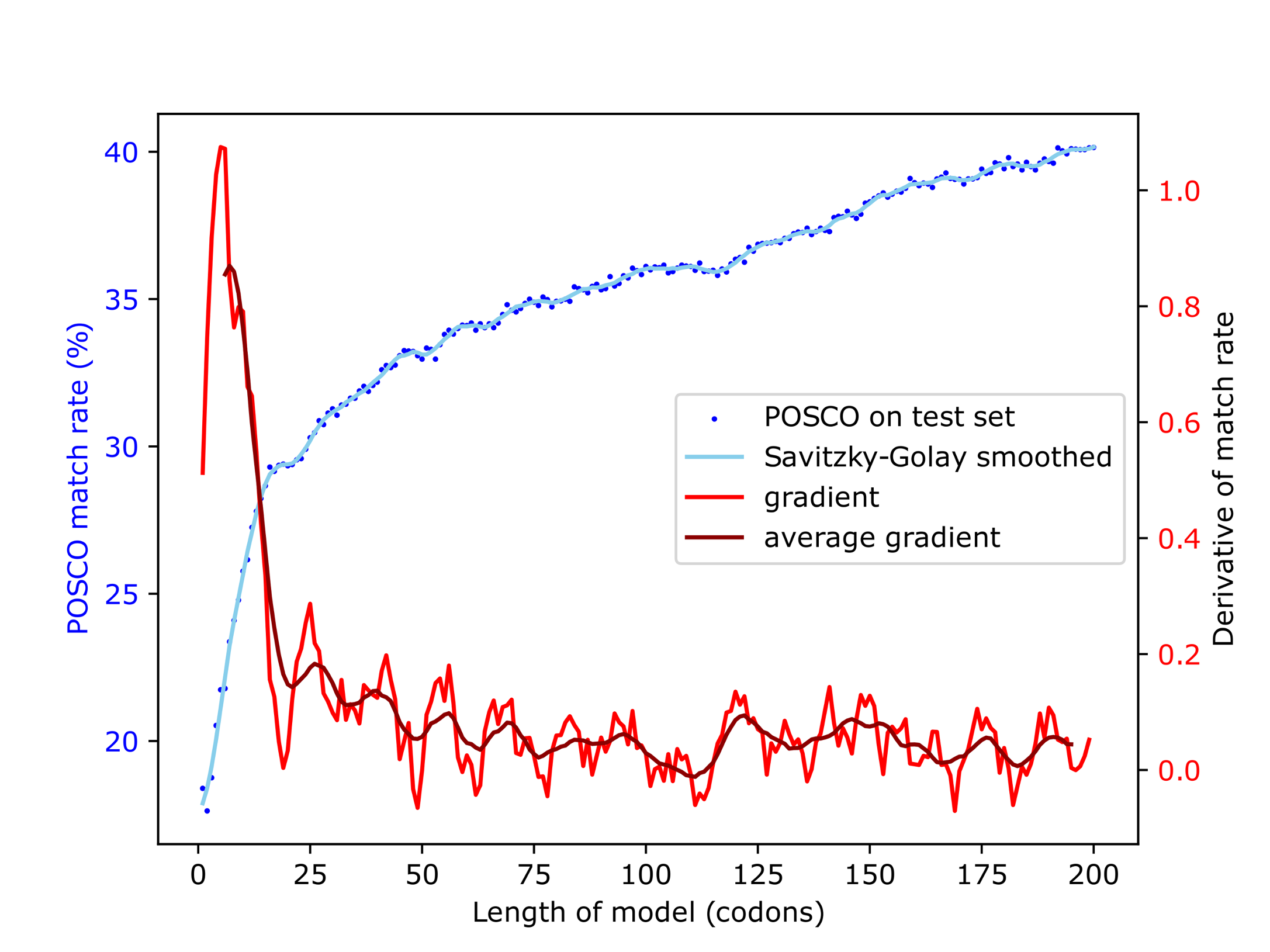

Supplement: S7 Fig — Blue dots represent the match rate as a function of the model length in codons for POSCO applied to the test set. Light blue curve is a Savitsky-Golay smoothed curve using a window length of 11 and a polynomial order of 3. Red curve is the difference between adjacent values of the smoothed curve (discrete derivative) and dark red curve is a sliding-window average with a window of 10. (PNG) [file pcbi.1014501.s007.png]

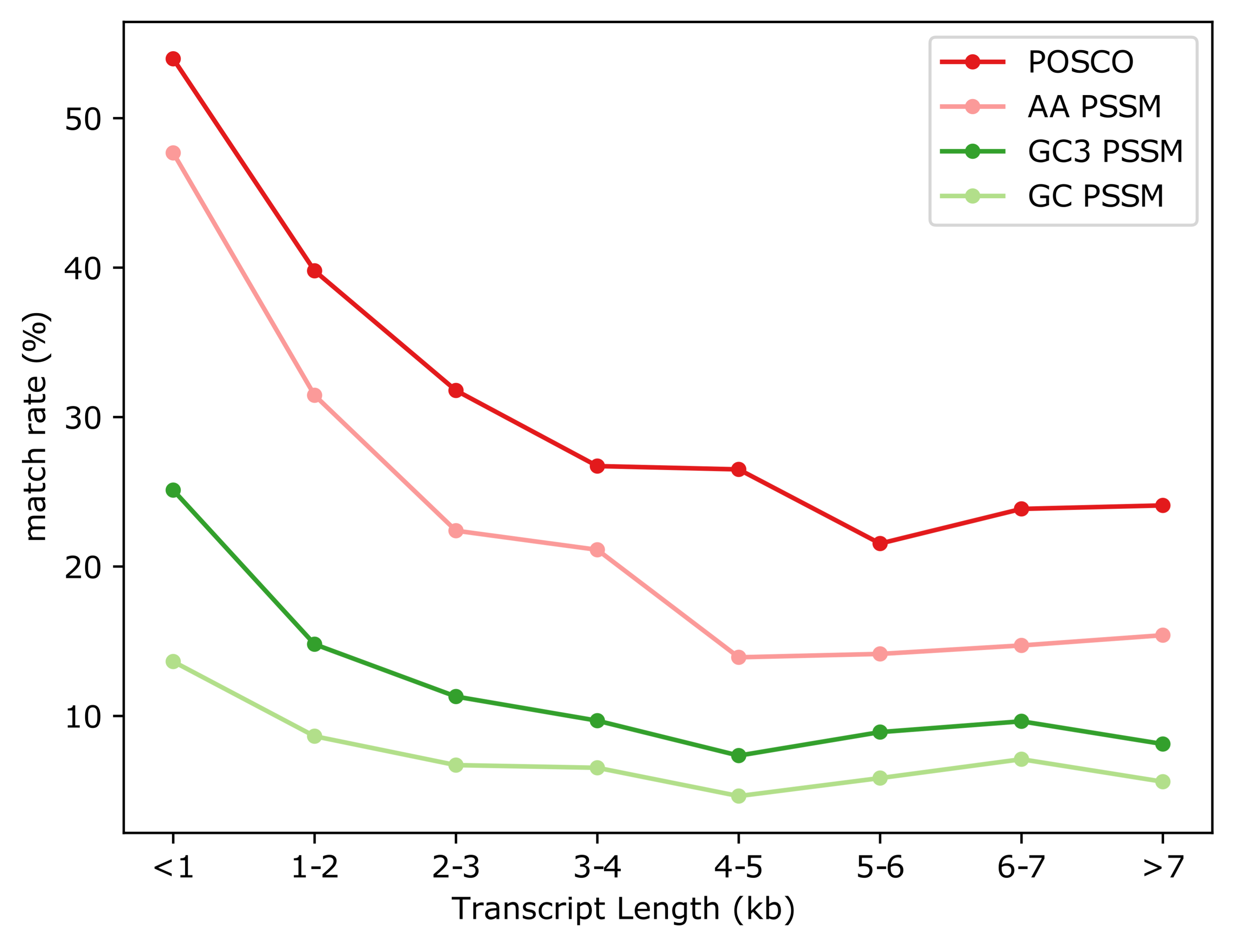

Supplement: S8 Fig — Curves show the fraction of start codons correctly identified per transcript for the POSCO PSSM (red), AA PSSM (pink), GC3 PSSM (dark green). (PNG) [file pcbi.1014501.s008.png]

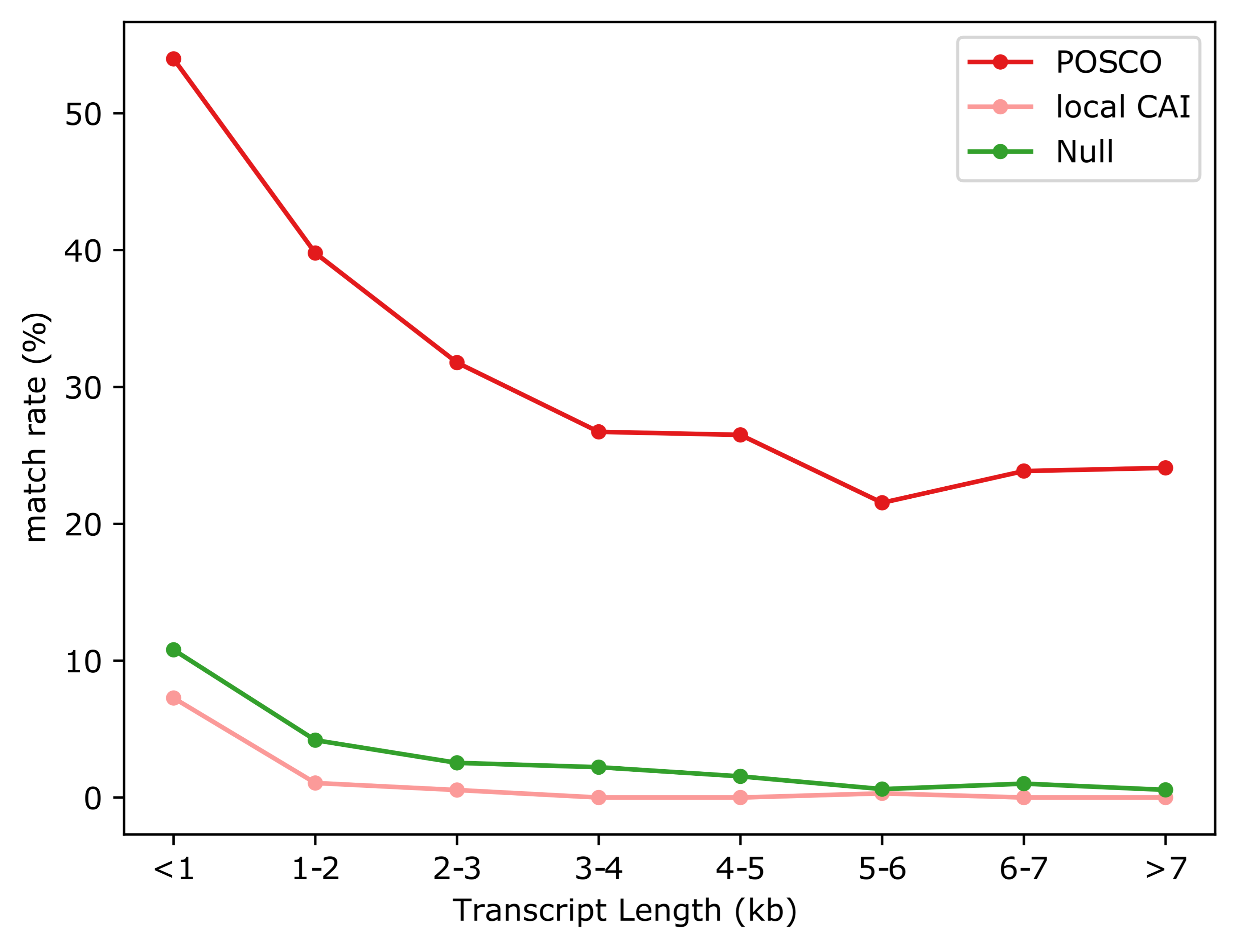

Supplement: S9 Fig — (PNG) [file pcbi.1014501.s009.png]

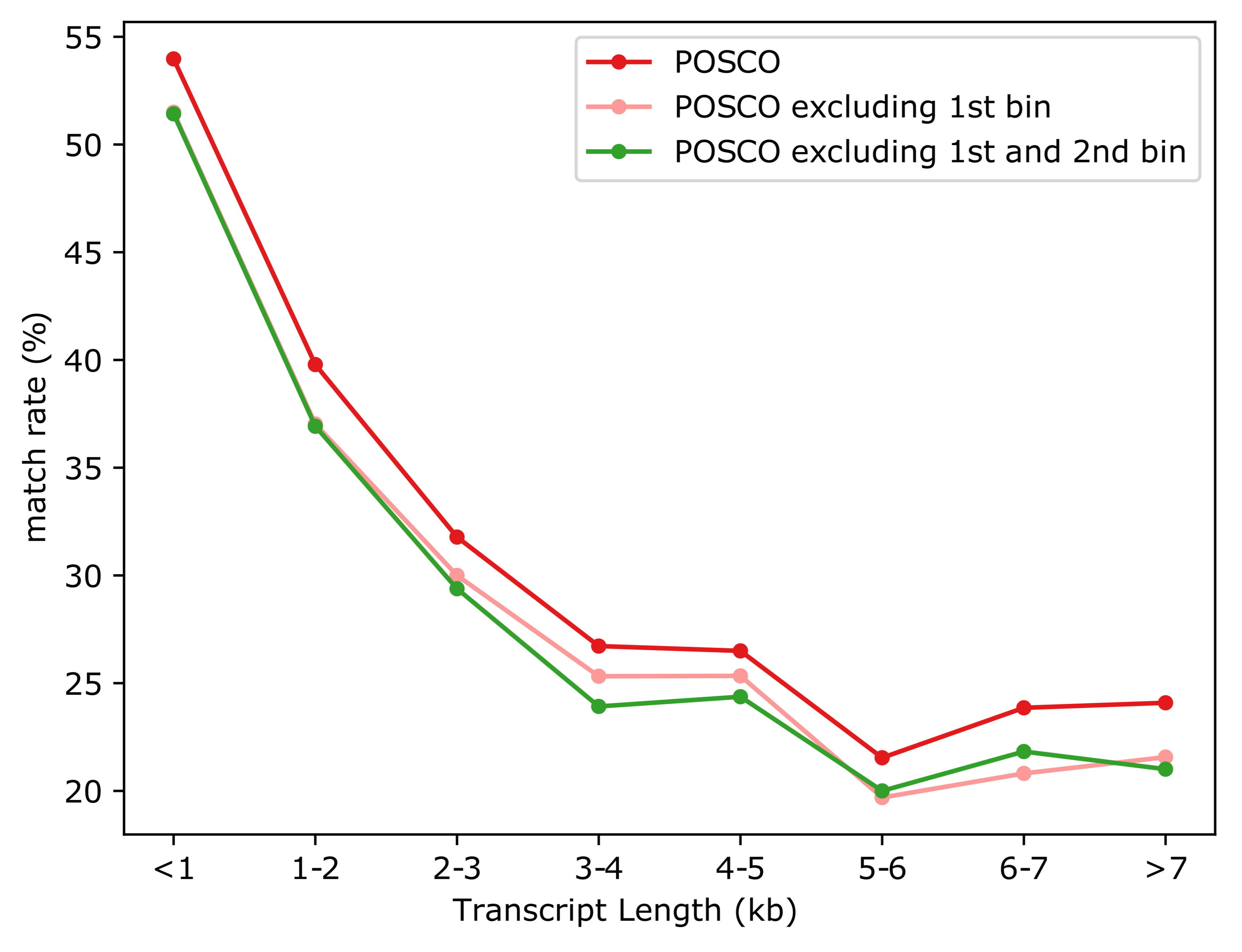

Supplement: S10 Fig — (PNG) [file pcbi.1014501.s010.png]

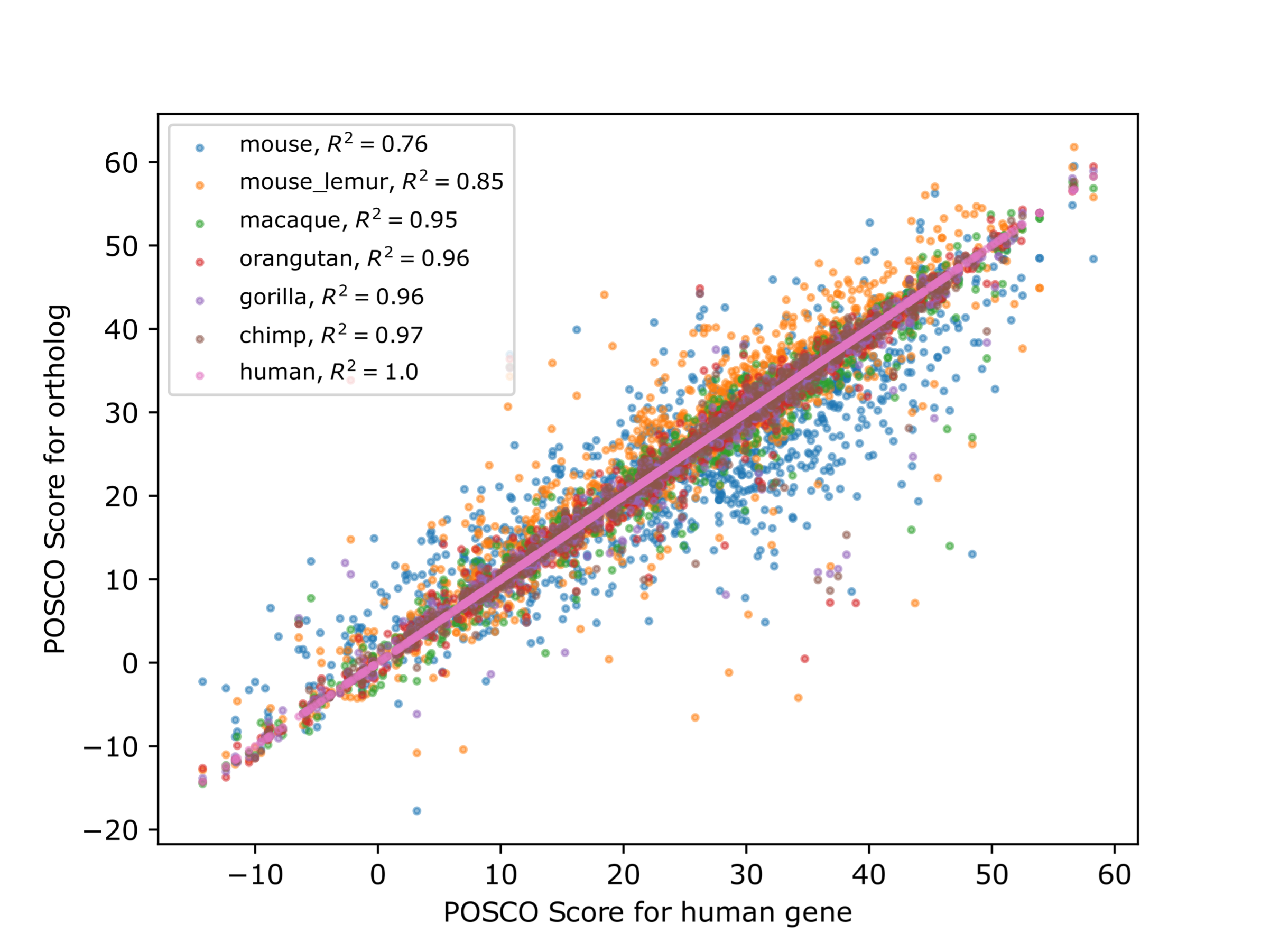

Supplement: S11 Fig — Correlations are computed comparing the POSCO scores for human and the ortholog in the comparison transcriptome. The correlation for human is 1.0 because it is comparing with the same transcriptome. (PNG) [file pcbi.1014501.s011.png]

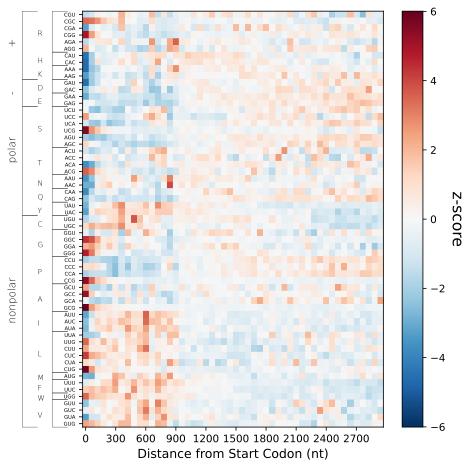

Supplement: S12 Fig — A larger region of significant z-scores is observed, up to 800 or 900 nt from the start codon compared to the human transcriptome S1 Fig. (PNG) [file pcbi.1014501.s012.png]

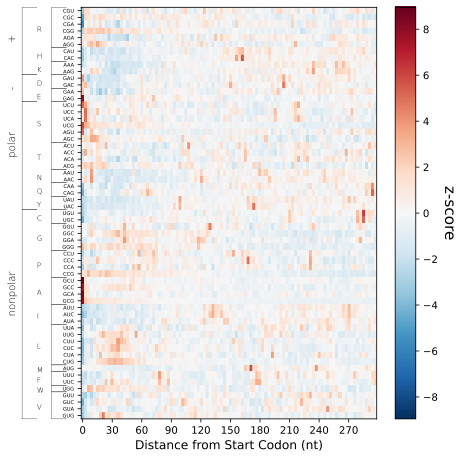

Supplement: S13 Fig — Sporadic bins of enrichment are observed throughout compared to the human heatmap, shown in S2 Fig. (PNG) [file pcbi.1014501.s013.png]

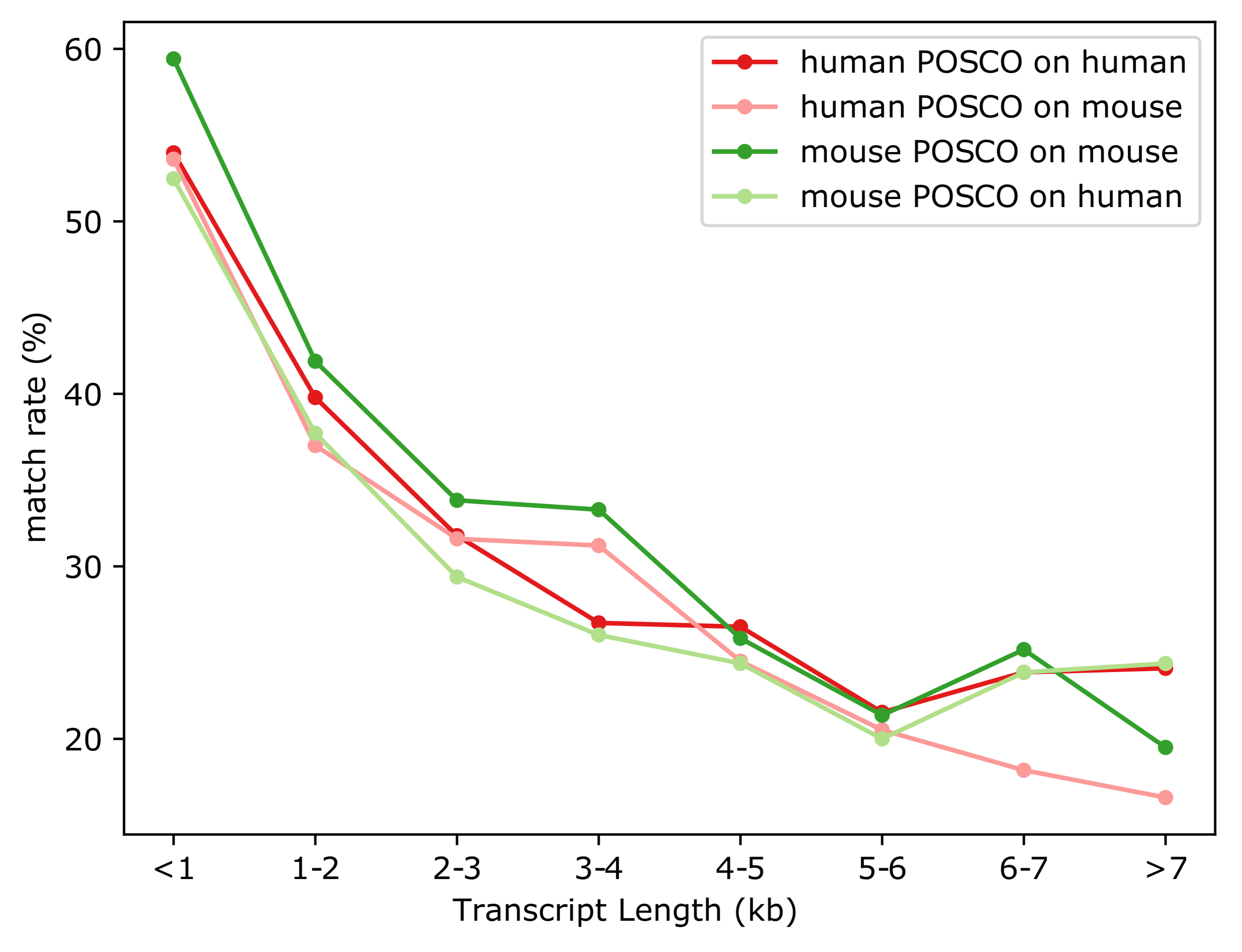

Supplement: S14 Fig — Both models are trained on length-stratified 80% of the transcriptome and tested on 10% of sequences in the test set. (PNG) [file pcbi.1014501.s014.png]

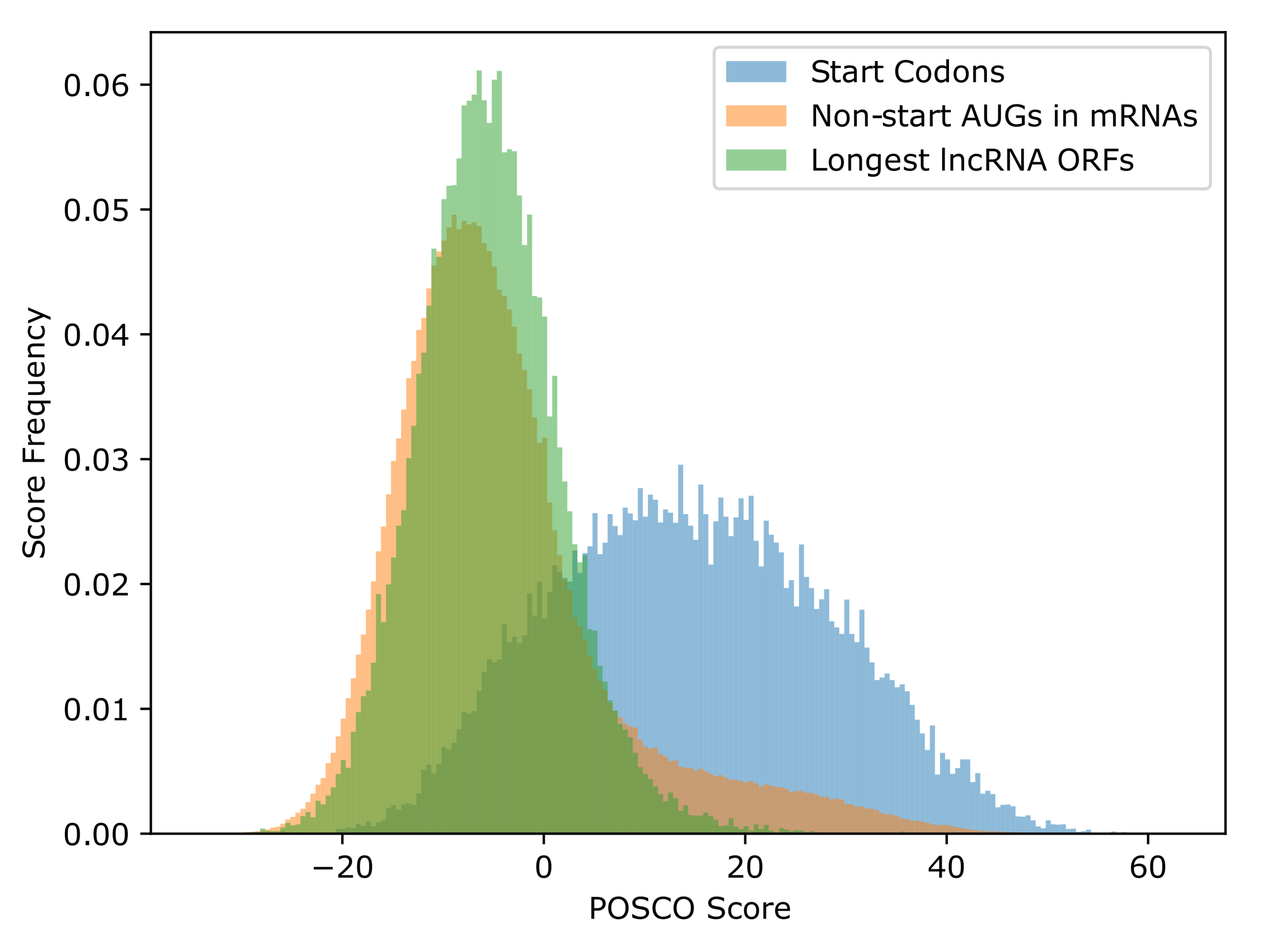

Supplement: S15 Fig — Mean and standard deviation from the non-start distribution is used to compute statistical significance of POSCO scores. (PNG) [file pcbi.1014501.s015.png]

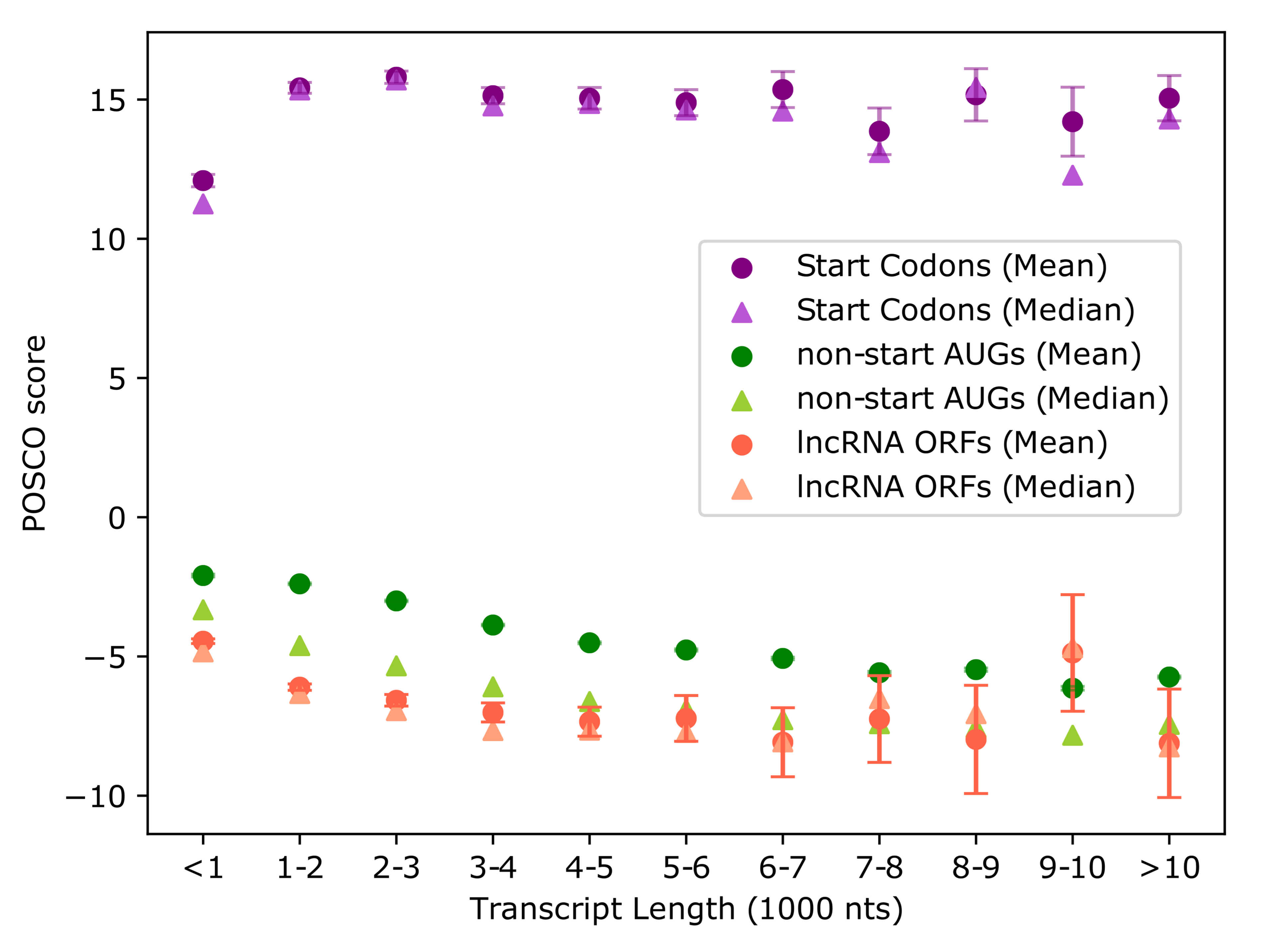

Supplement: S16 Fig — In addition, this also compares to the scores for the AUG in the longest ORF for lncRNAs using the same size bins. (PNG) [file pcbi.1014501.s016.png]

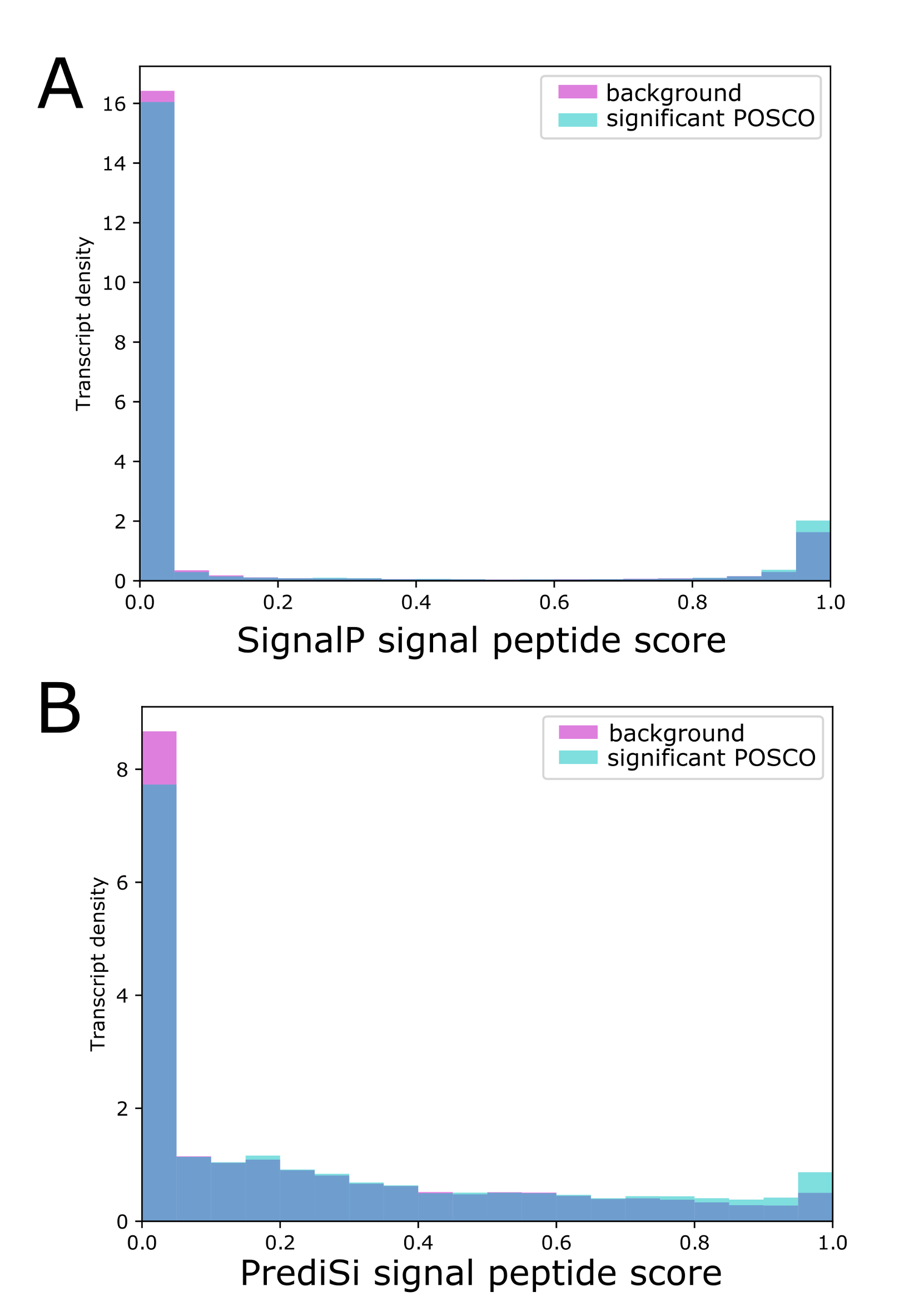

Supplement: S17 Fig — The darker blue/purple color is the overlap between two semi-transparent histograms. A. SignalP distribution. B. PrediSi distribution. (PNG) [file pcbi.1014501.s017.png]

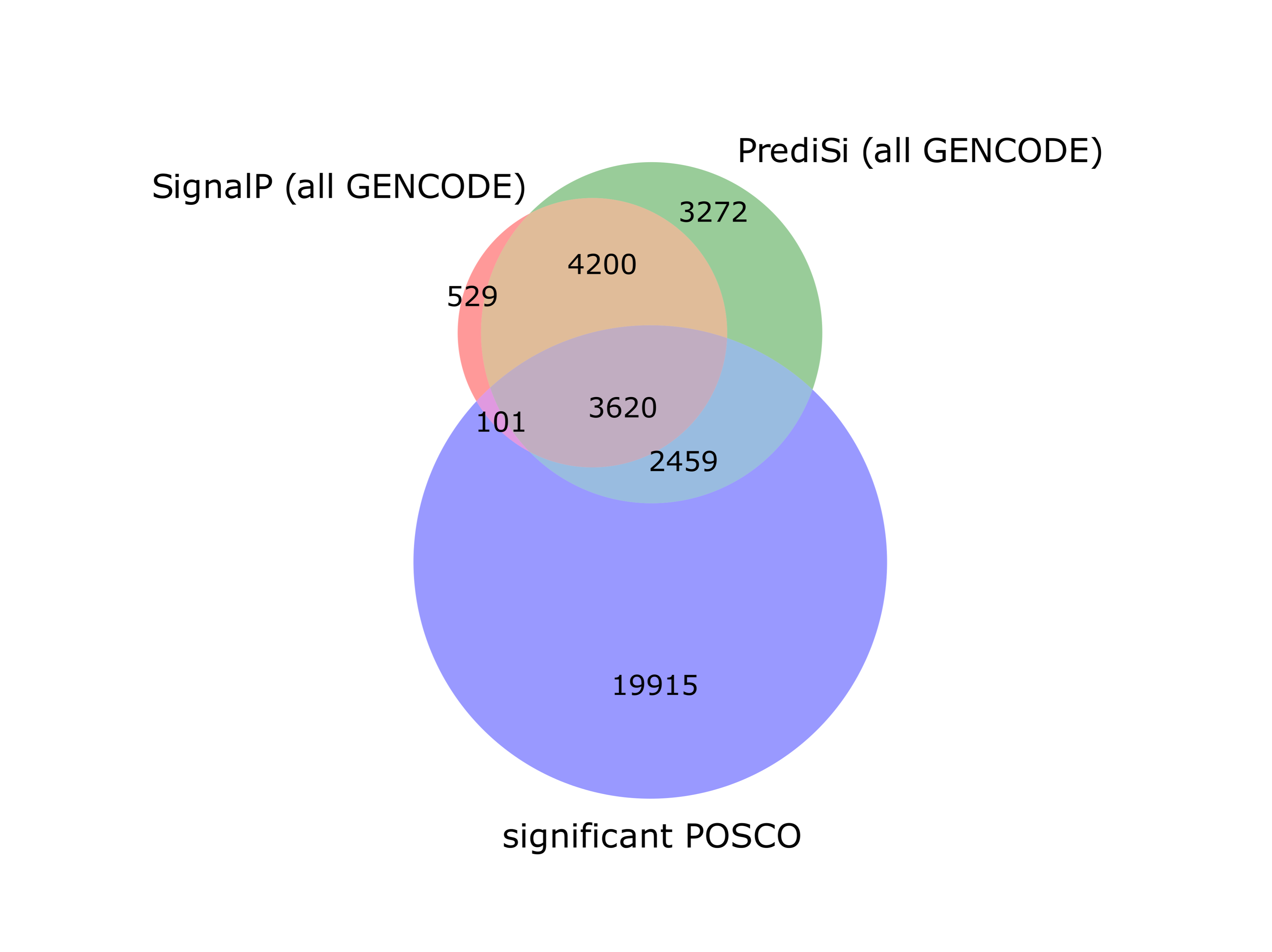

Supplement: S18 Fig — (PNG) [file pcbi.1014501.s018.png]

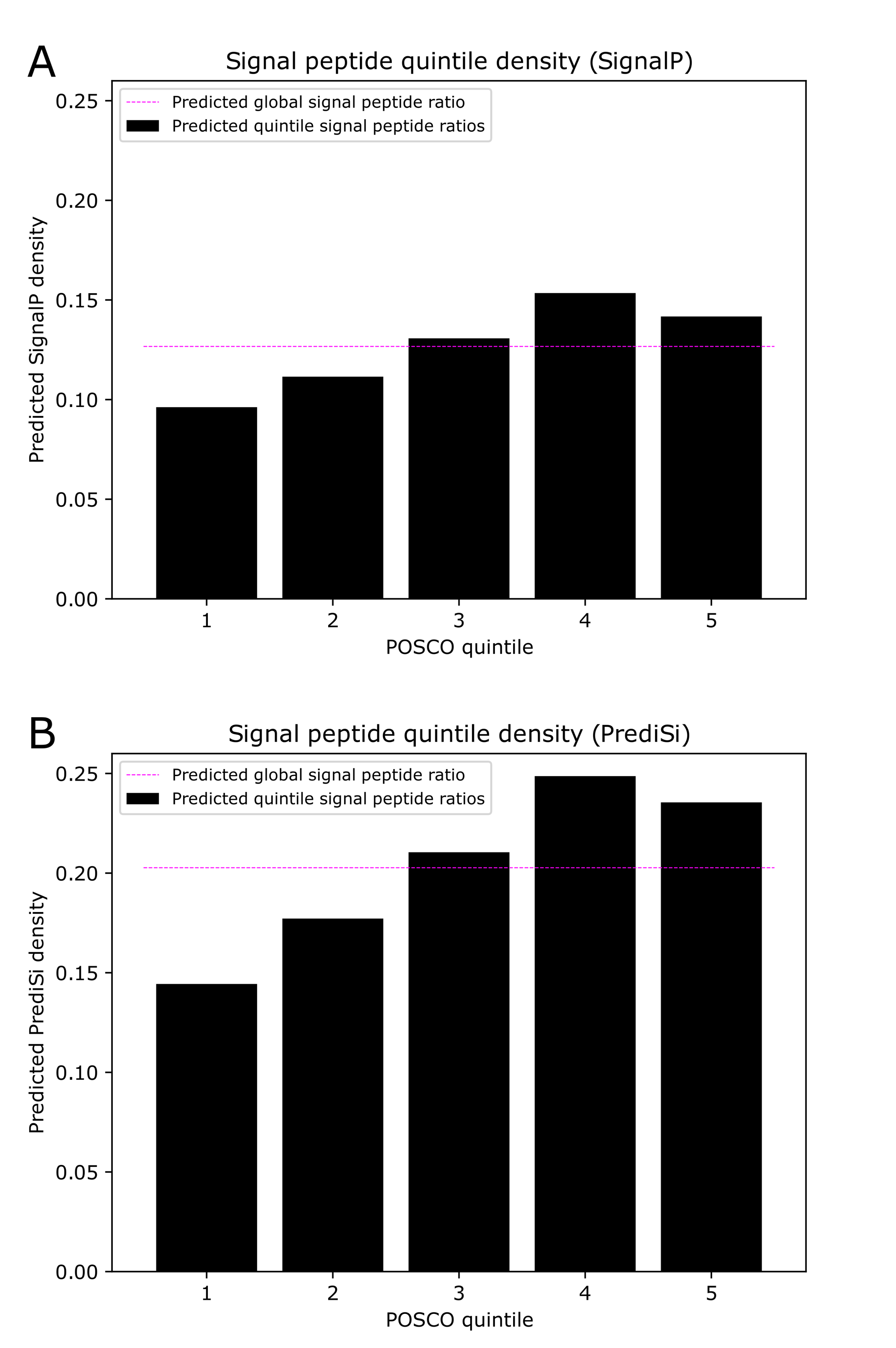

Supplement: S19 Fig — Magenta line indicates global predicted signal peptide density. A. SignalP predictions. B. PrediSi predictions. (PNG) [file pcbi.1014501.s019.png]

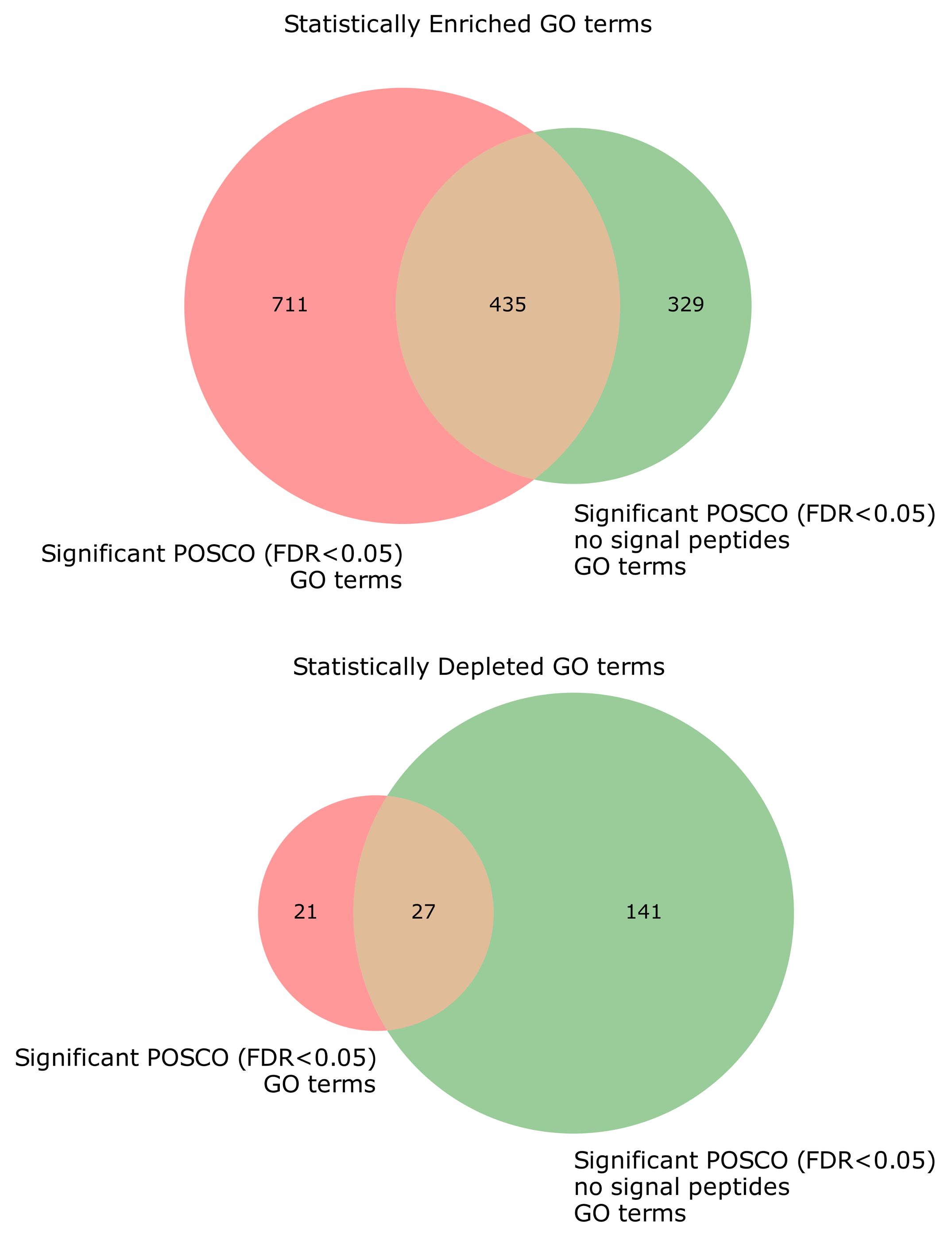

Supplement: S20 Fig — Top Venn shows statistically enriched GO terms, and bottom Venn shows statistically depleted terms. (PNG) [file pcbi.1014501.s020.png]

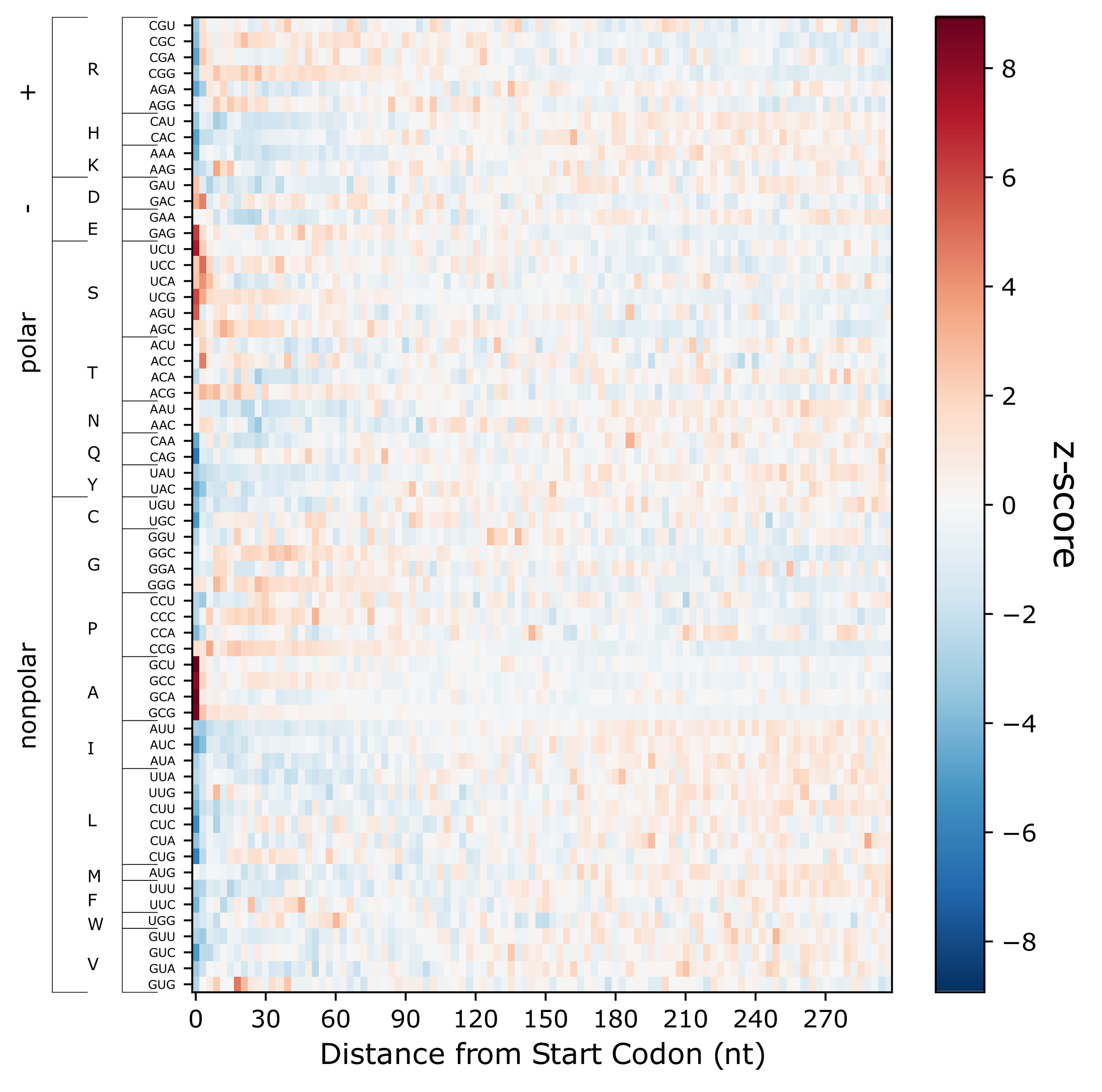

Supplement: S21 Fig — (PNG) [file pcbi.1014501.s021.png]

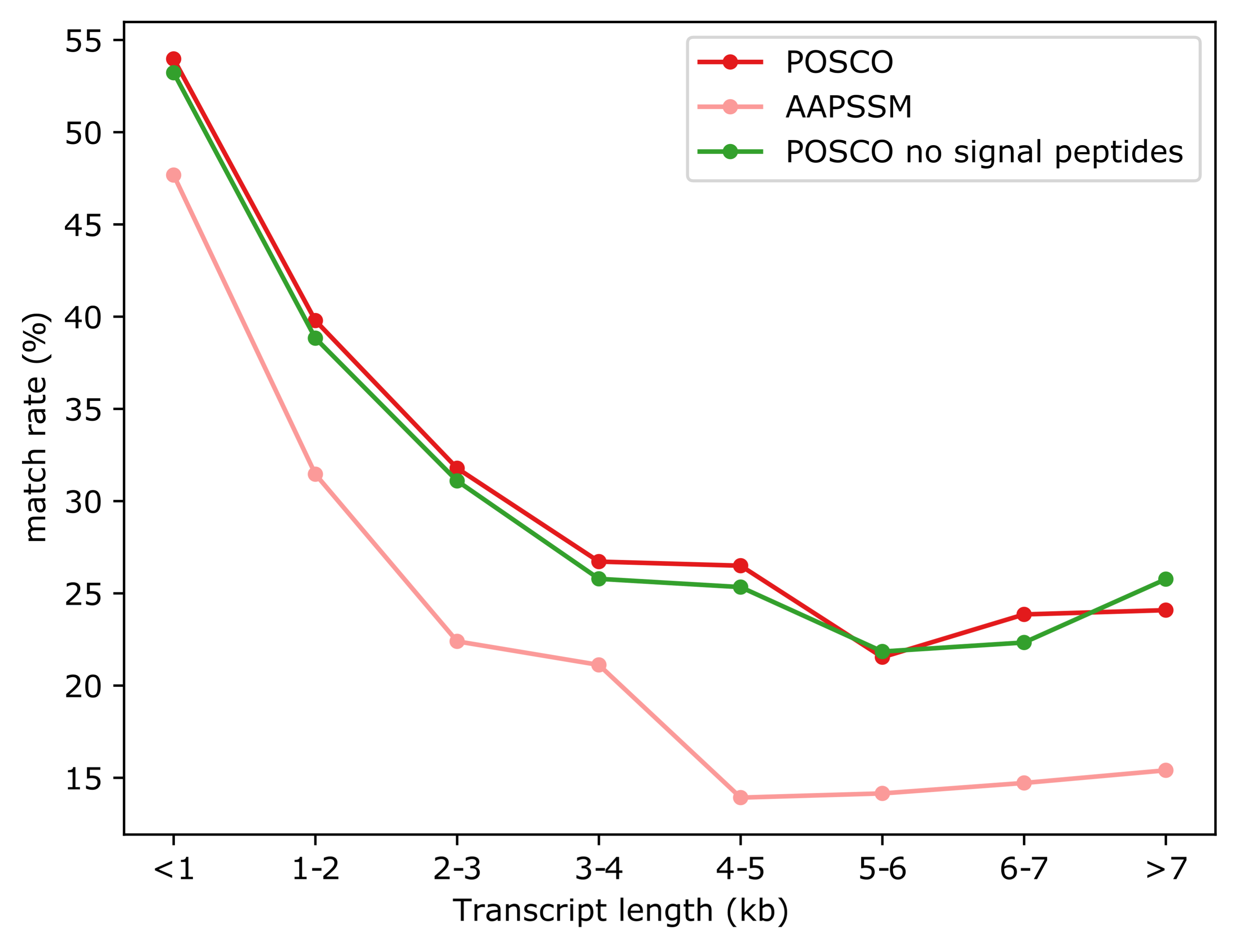

Supplement: S22 Fig — Transcripts filtered by SignalP and PrediSi scores greater than 0.5. (PNG) [file pcbi.1014501.s022.png]

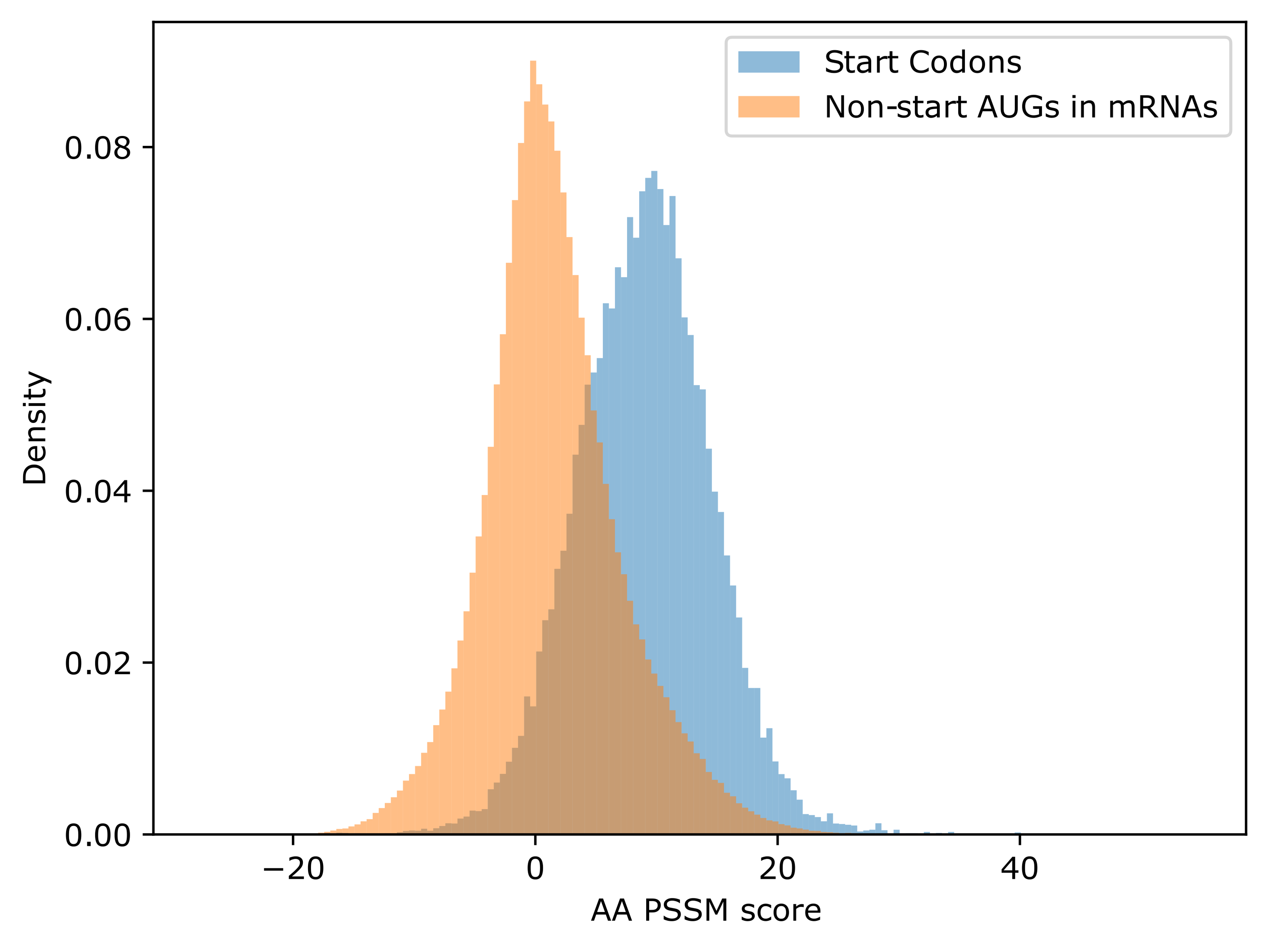

Supplement: S23 Fig — (PNG) [file pcbi.1014501.s023.png]

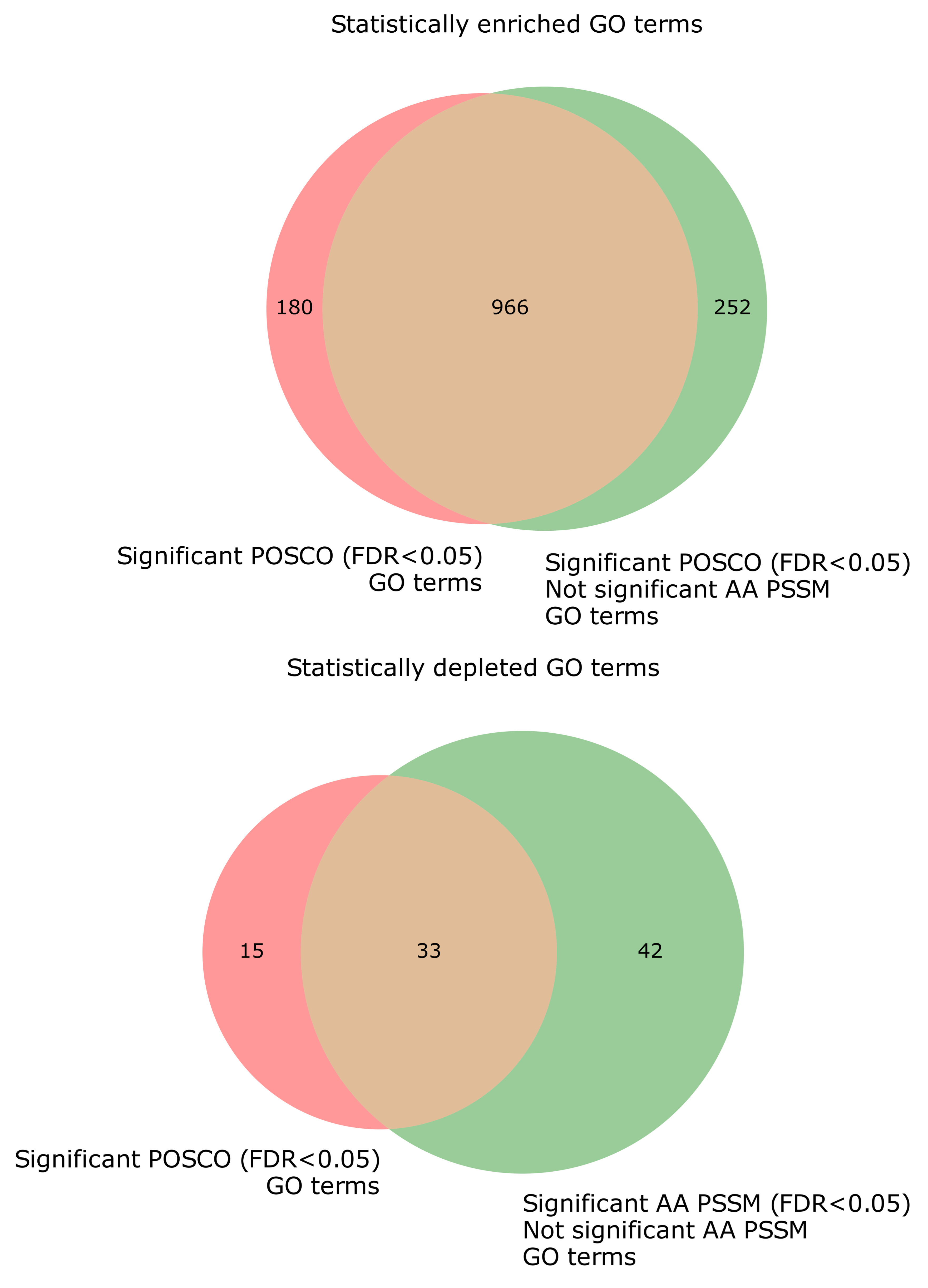

Supplement: S24 Fig — (PNG) [file pcbi.1014501.s024.png]

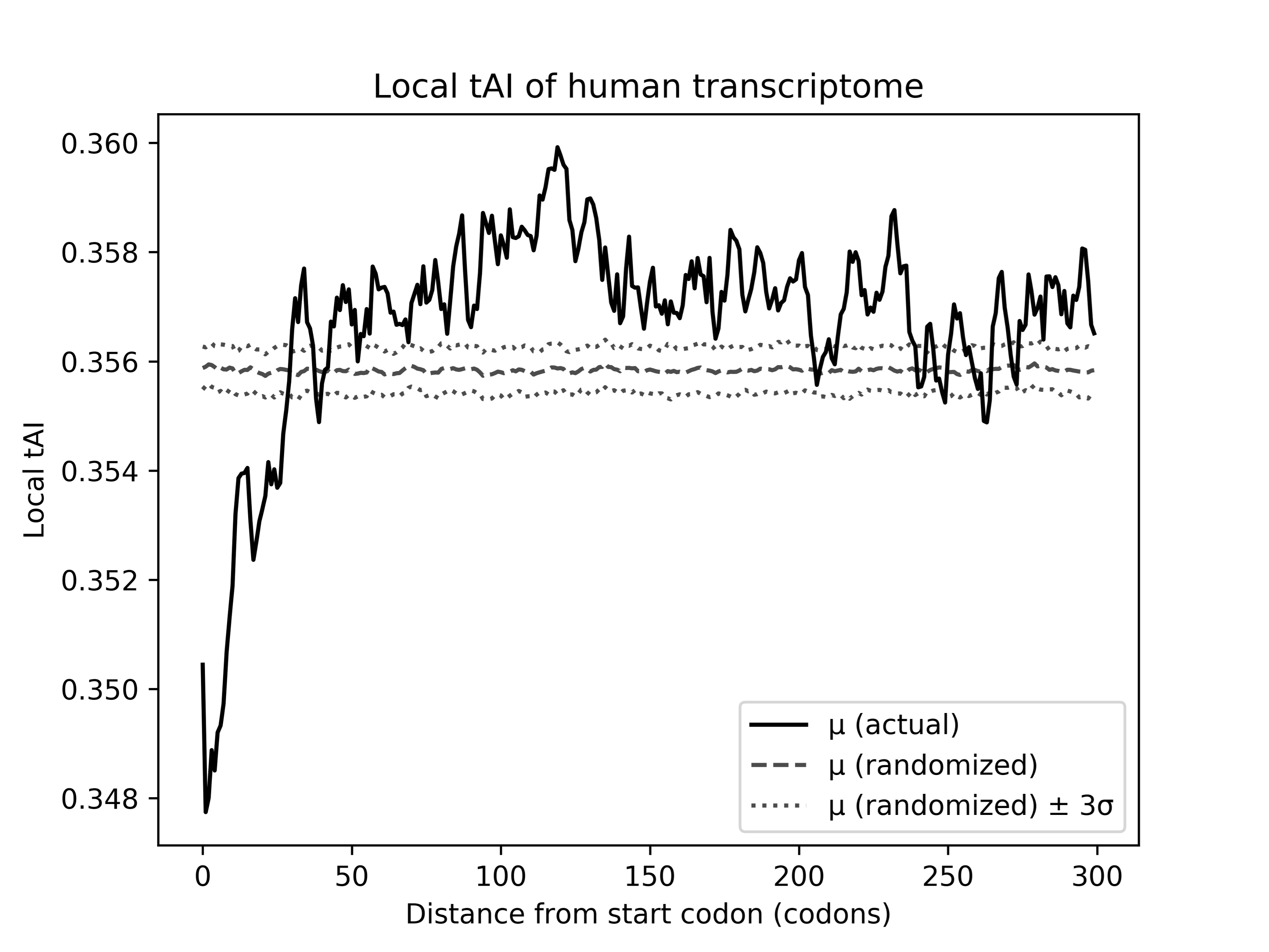

Supplement: S25 Fig — A distinct ramp is seen from codons 0–50. The dashed line shows the average local tAI across 100 randomizations of the coding sequence. Dotted lines indicates three standard deviations above and below the randomized mean. (PNG) [file pcbi.1014501.s025.png]

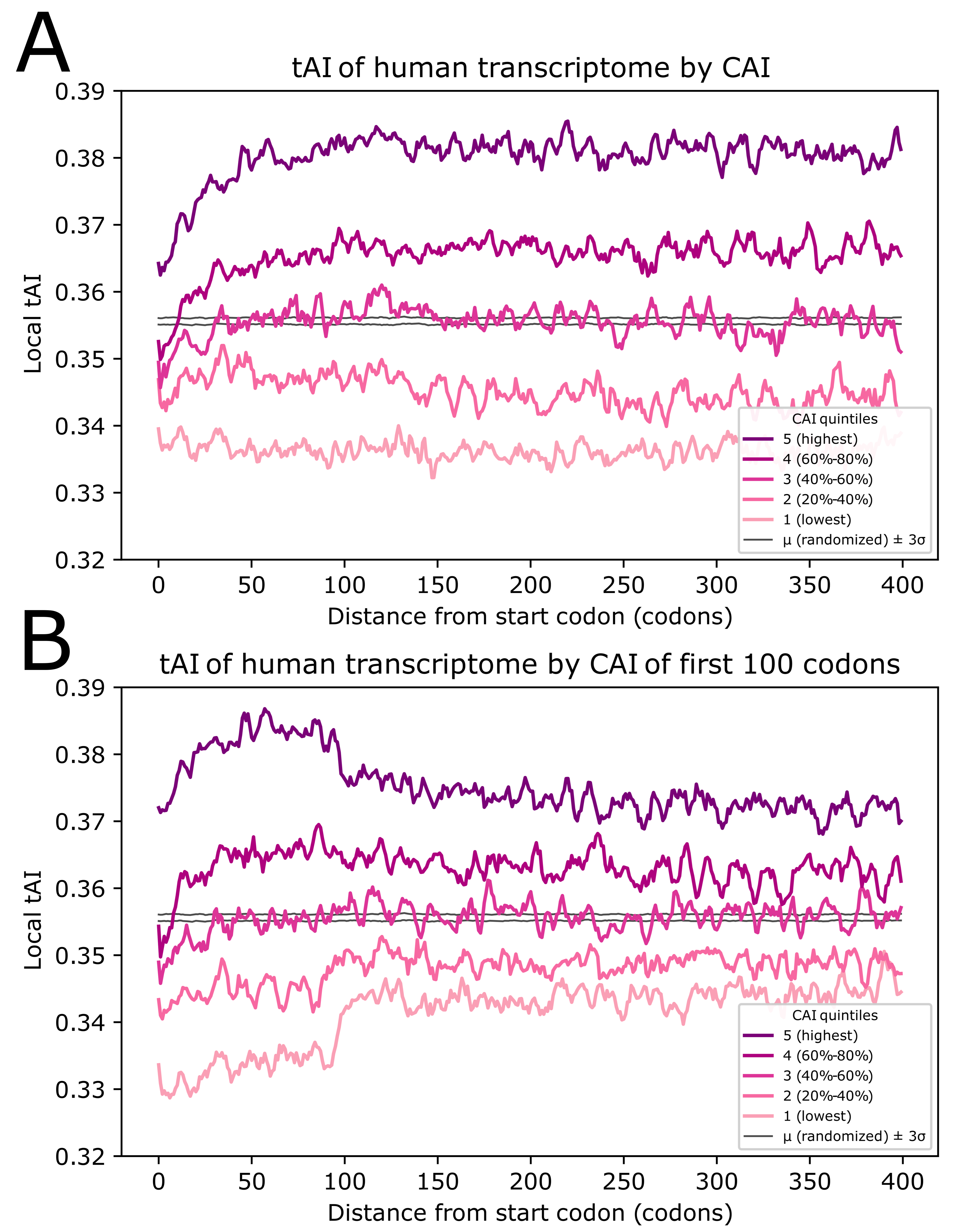

Supplement: S26 Fig — Different colors represent tAI curves for individual quintiles. Grey lines indicate the statistical upper and lower bounds of local tAI averaged over 100 randomizations of all coding sequences. A. Global CAI. B. Regional CAI. (PNG) [file pcbi.1014501.s026.png]

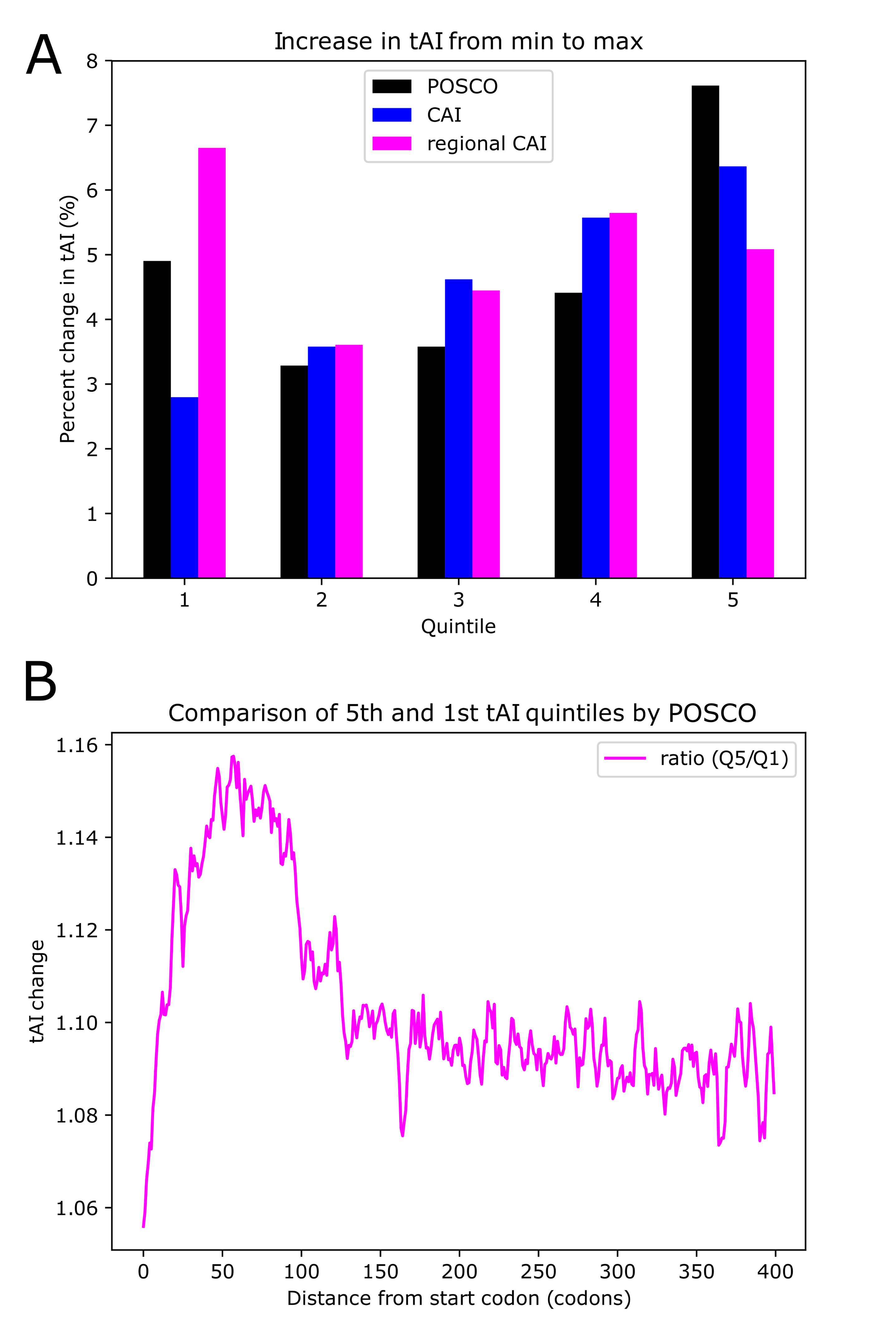

Supplement: S27 Fig — A. Side-by-side percent increase in tAI from lowest point to highest point for each POSCO, CAI, and regional CAI quintile. B. Ratio of the tAI for fifth and first POSCO quintiles as a function of position for 5-codon windows. (PNG) [file pcbi.1014501.s027.png]

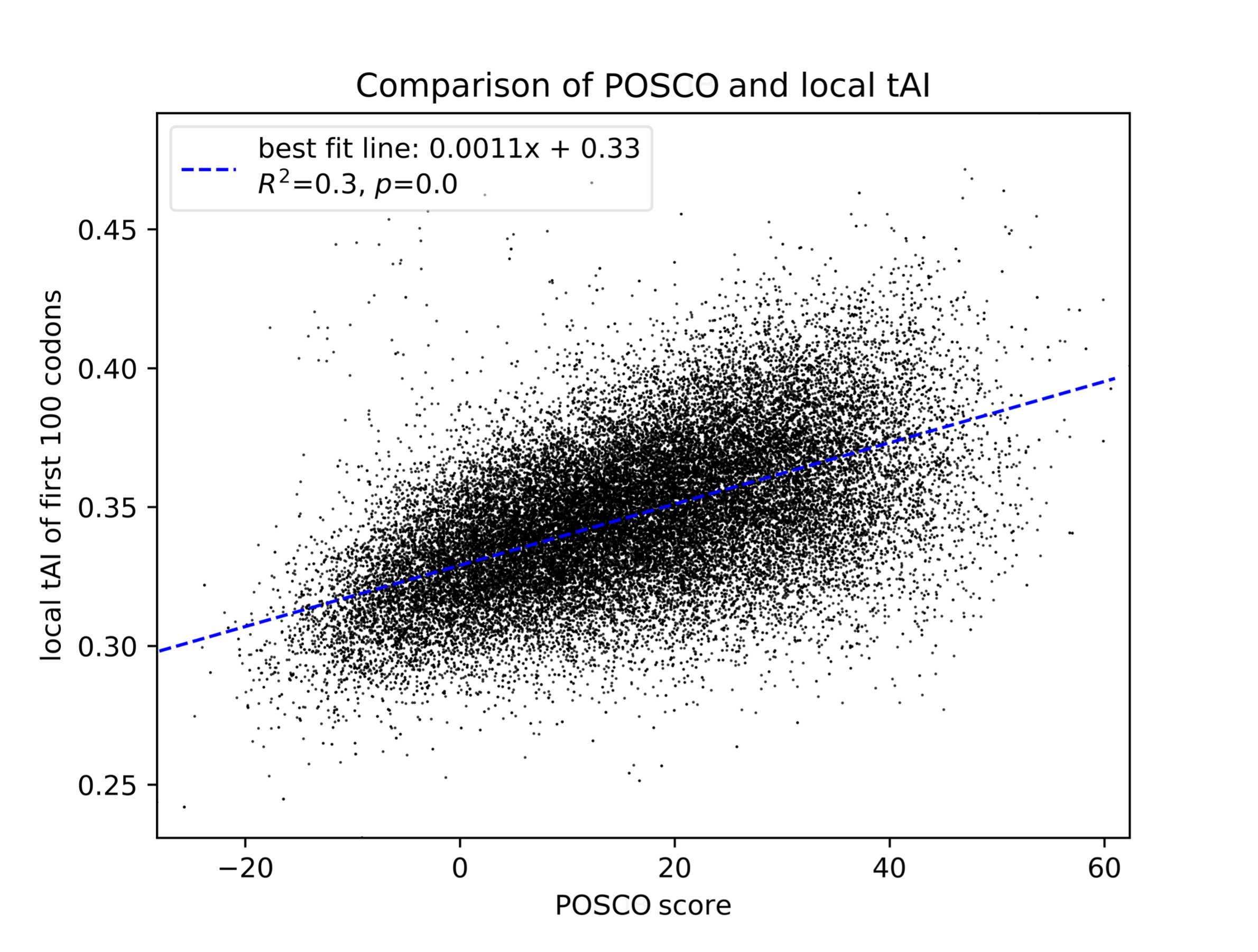

Supplement: S28 Fig — Each dot corresponds to a transcript. The dashed line is the best linear fit to the scatter data. (PNG) [file pcbi.1014501.s028.png]

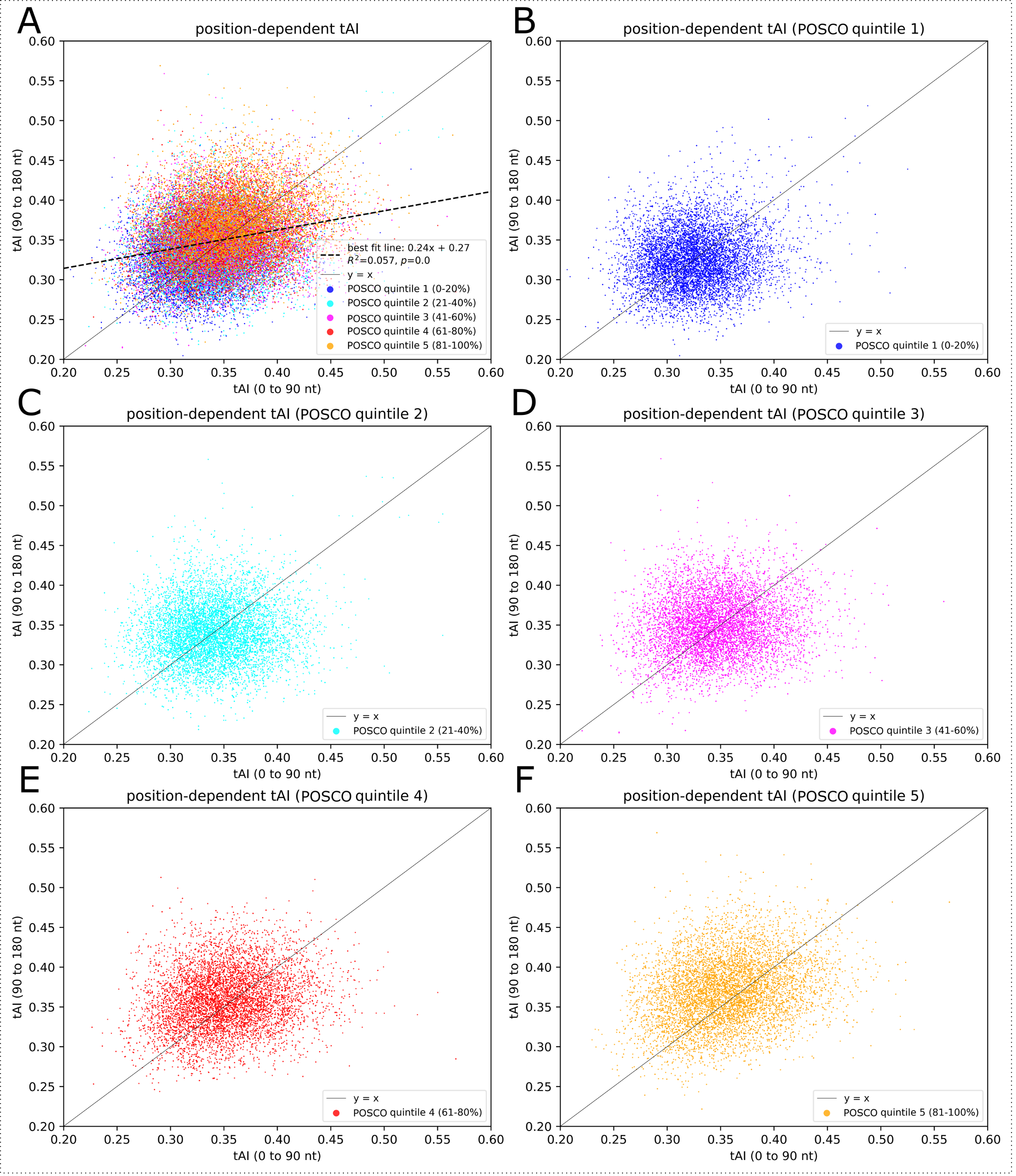

Supplement: S29 Fig — A. All quintiles with best fit line. B. First quintile (lowest POSCO); dark blue. C. Second quintile; cyan. D. Third quintile; magenta. E. Fourth quintile; red. F. Fifth quintile (highest POSCO); yellow. (PNG) [file pcbi.1014501.s029.png]

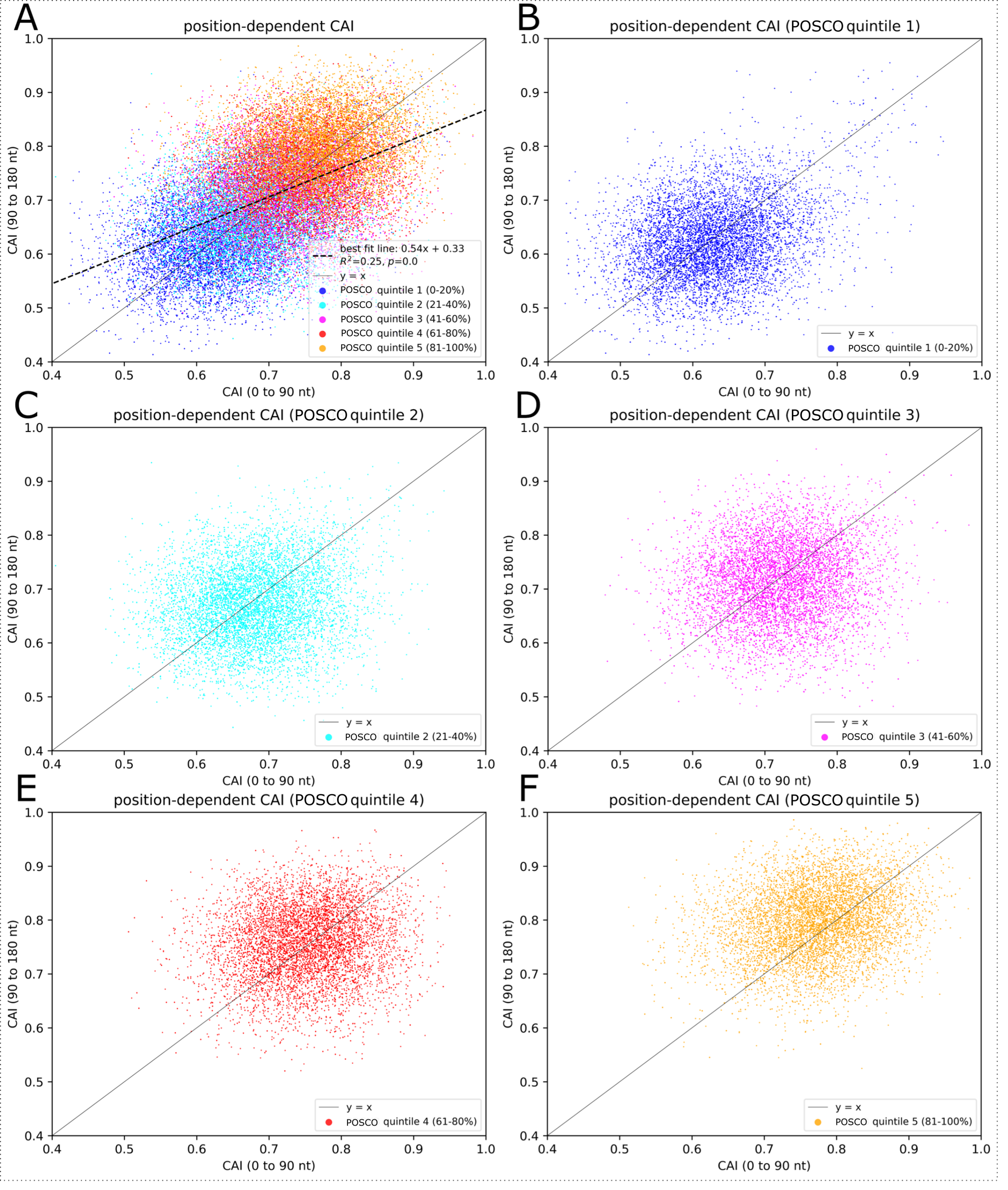

Supplement: S30 Fig — A. All quintiles with best fit line. B. First quintile (lowest POSCO); dark blue. C. Second quintile; cyan. D. Third quintile; magenta. E. Fourth quintile; red. F. Fifth quintile (highest POSCO); yellow. (PNG) [file pcbi.1014501.s030.png]

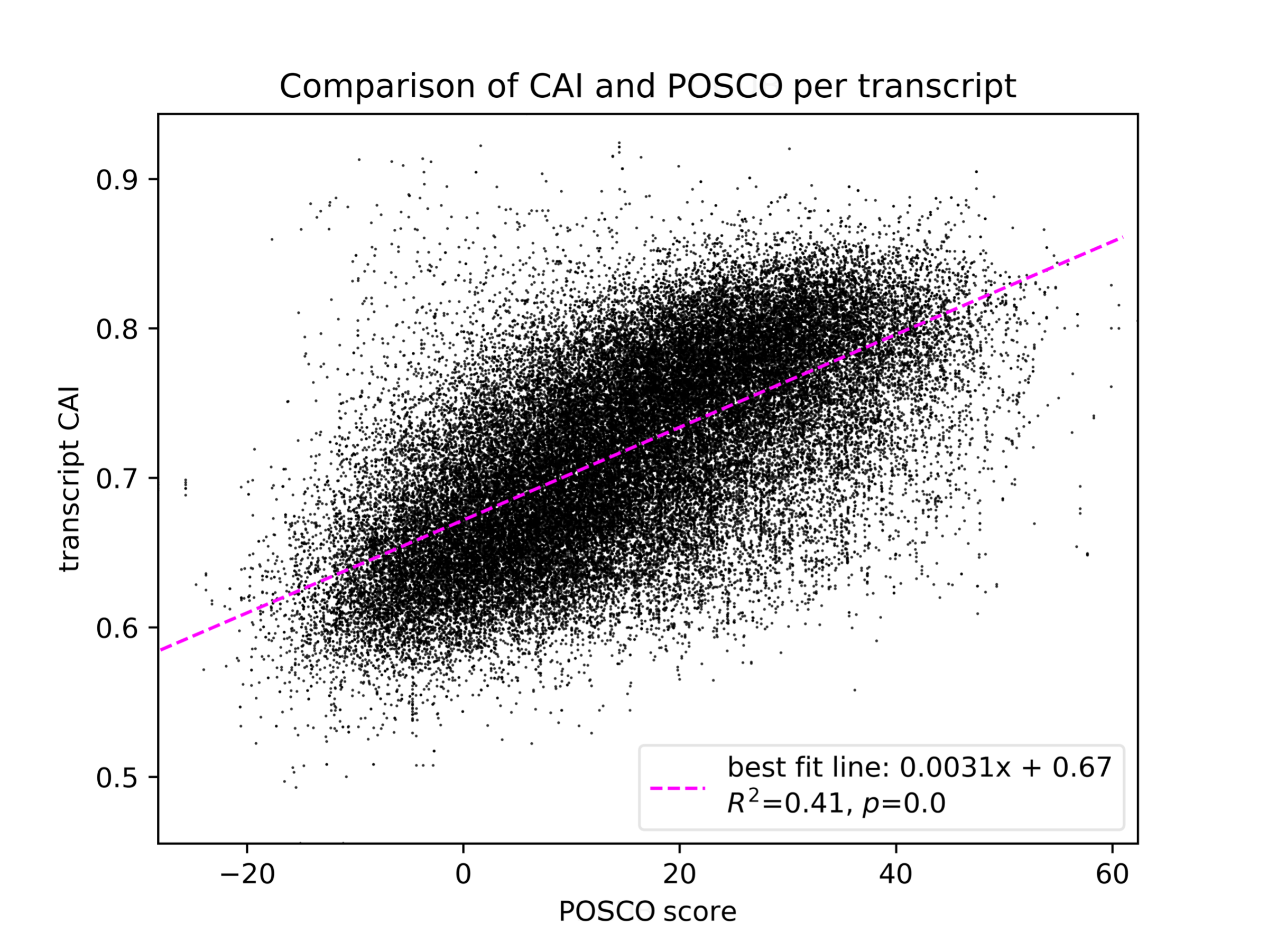

Supplement: S31 Fig — Each dot corresponds to a transcript. The dashed magenta line is the best linear fit to the scatter data. (PNG) [file pcbi.1014501.s031.png]

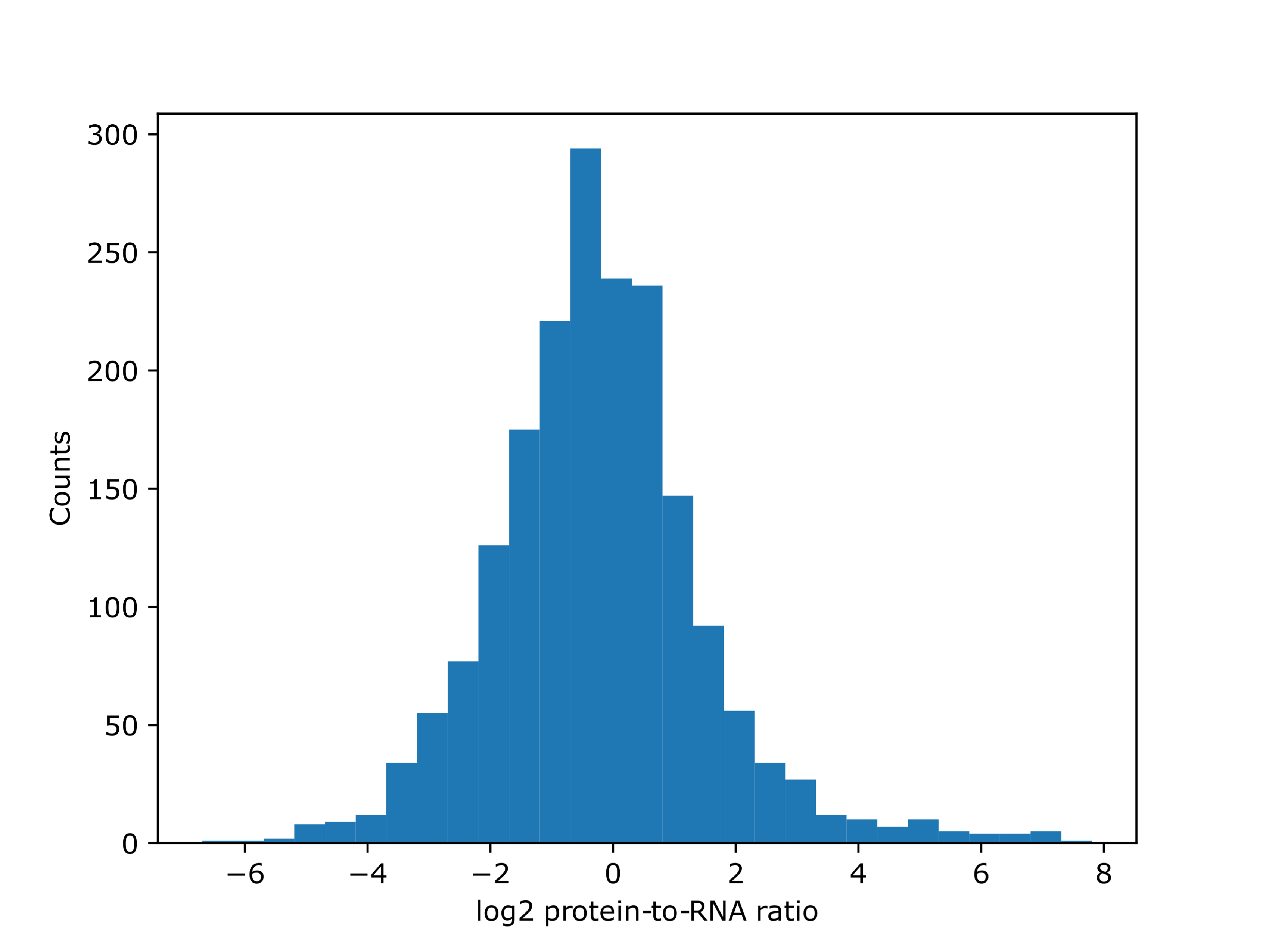

Supplement: S32 Fig — (PNG) [file pcbi.1014501.s032.png]

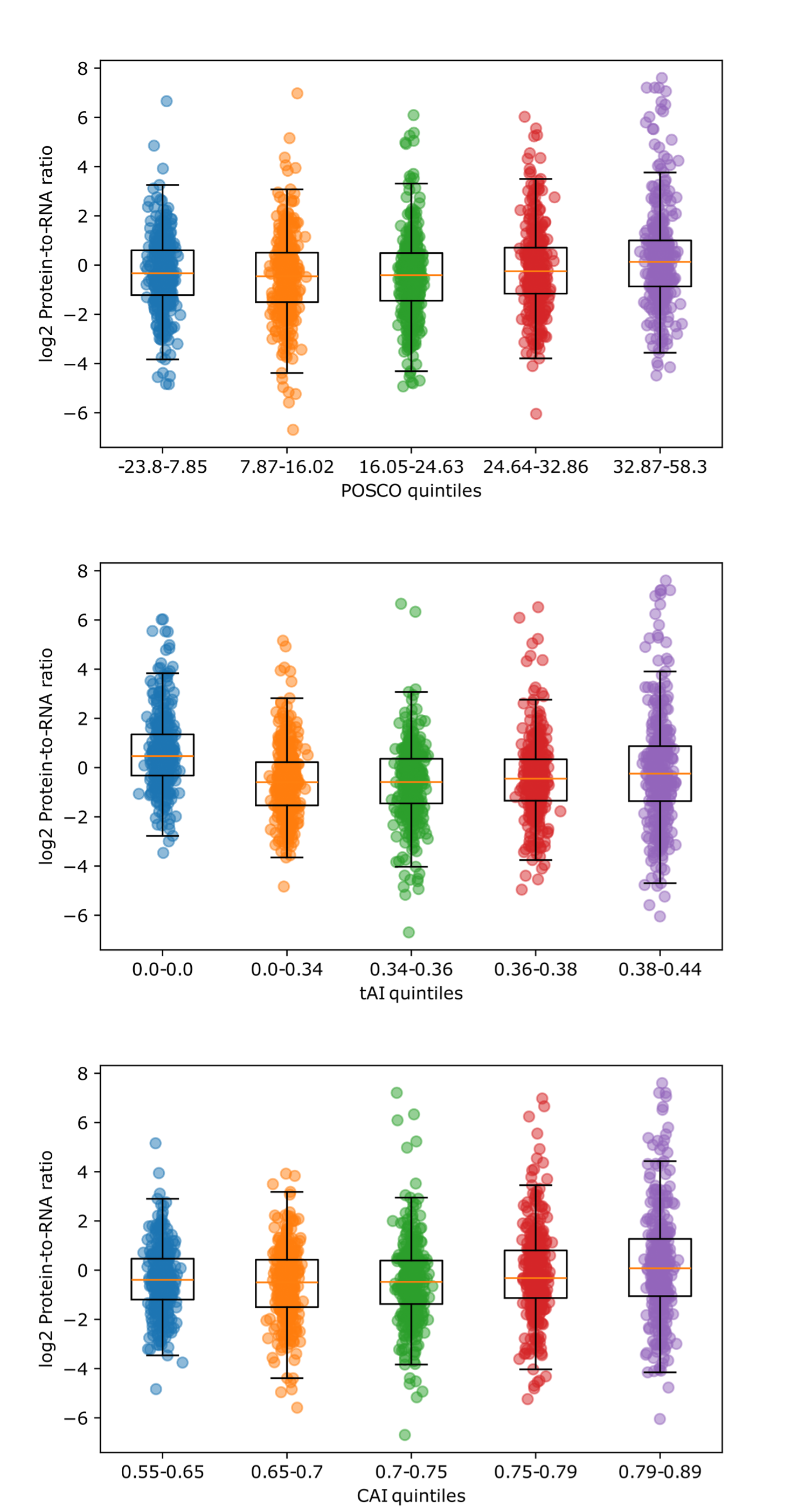

Supplement: S33 Fig — (PNG) [file pcbi.1014501.s033.png]

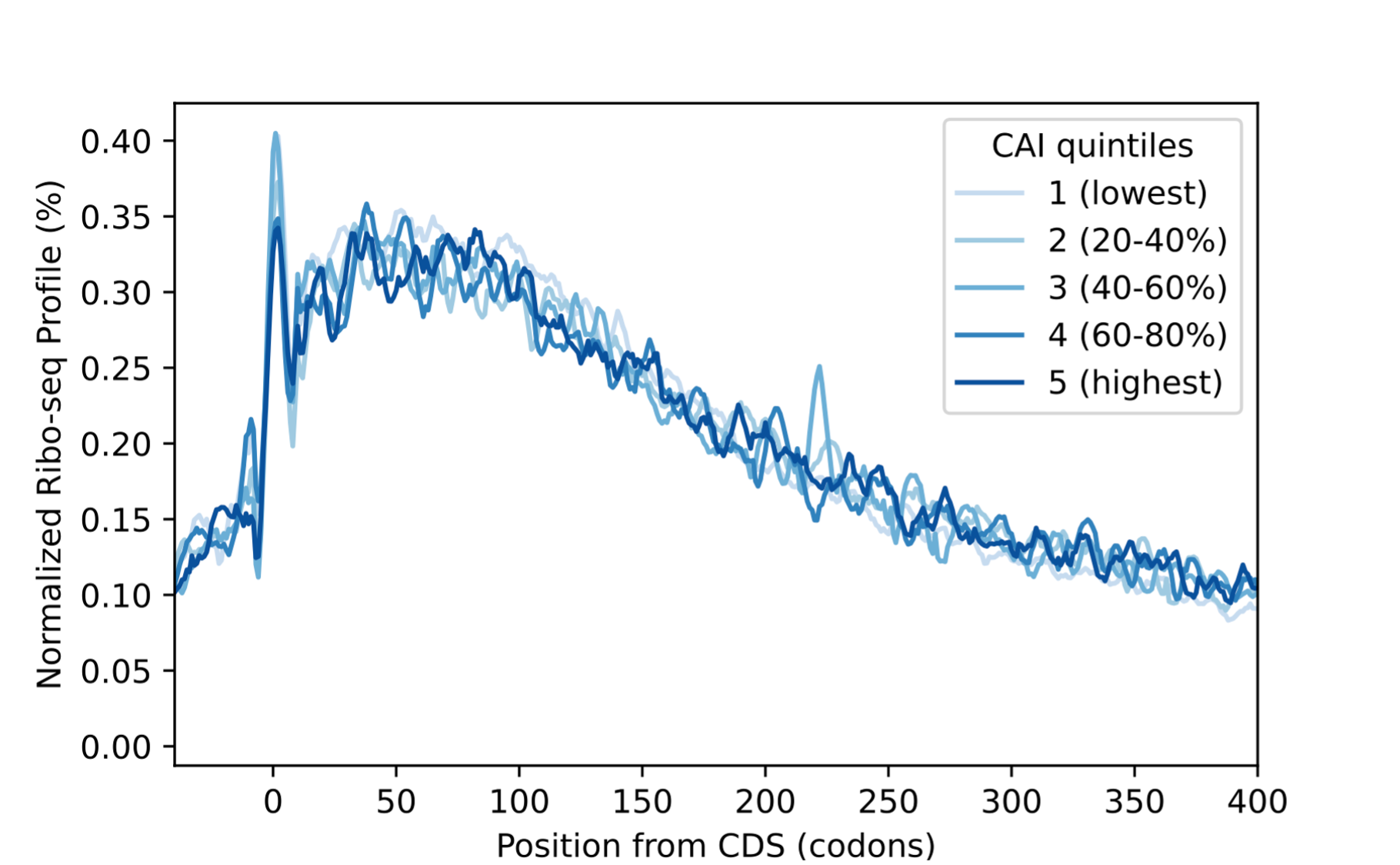

Supplement: S34 Fig — Read positions are A-shifted by 15-nt, and smoothed using a Savitzky-Golay filter. (PNG) [file pcbi.1014501.s034.png]

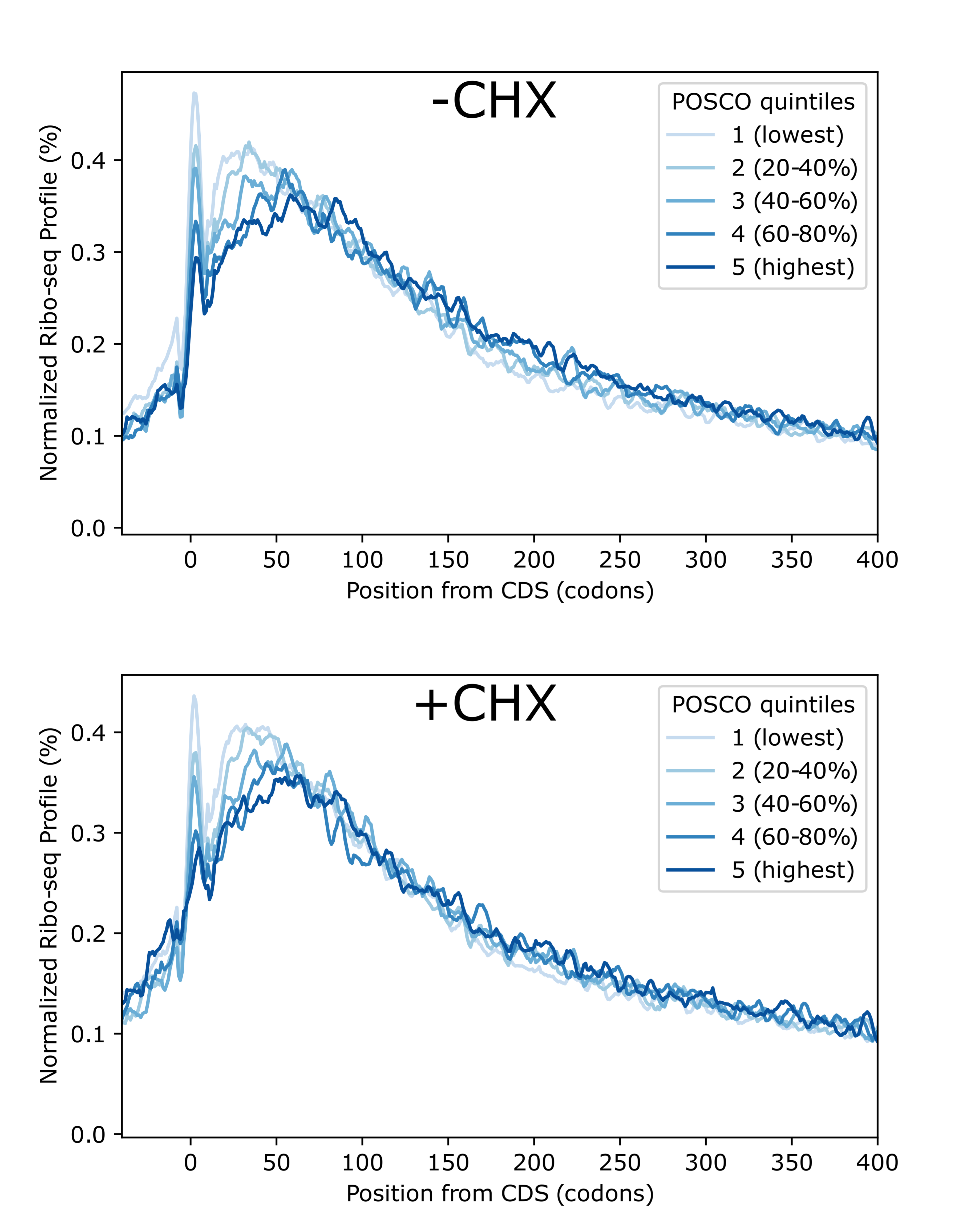

Supplement: S35 Fig — A. Without CHX. B. With CHX. (PNG) [file pcbi.1014501.s035.png]

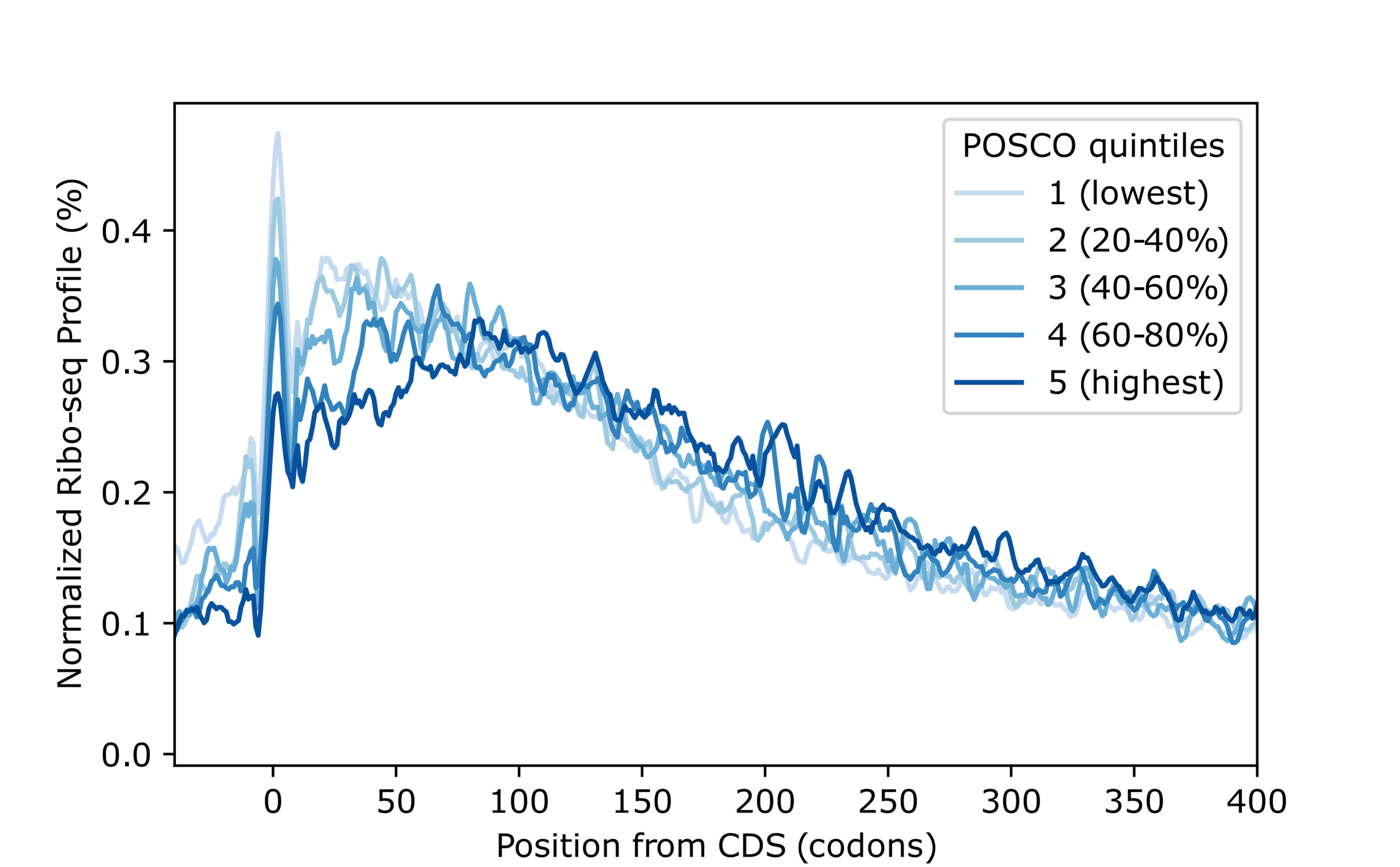

Supplement: S36 Fig — Read positions are A-shifted by 15-nt, and smoothed using a Savitzky-Golay filter. (PNG) [file pcbi.1014501.s036.png]

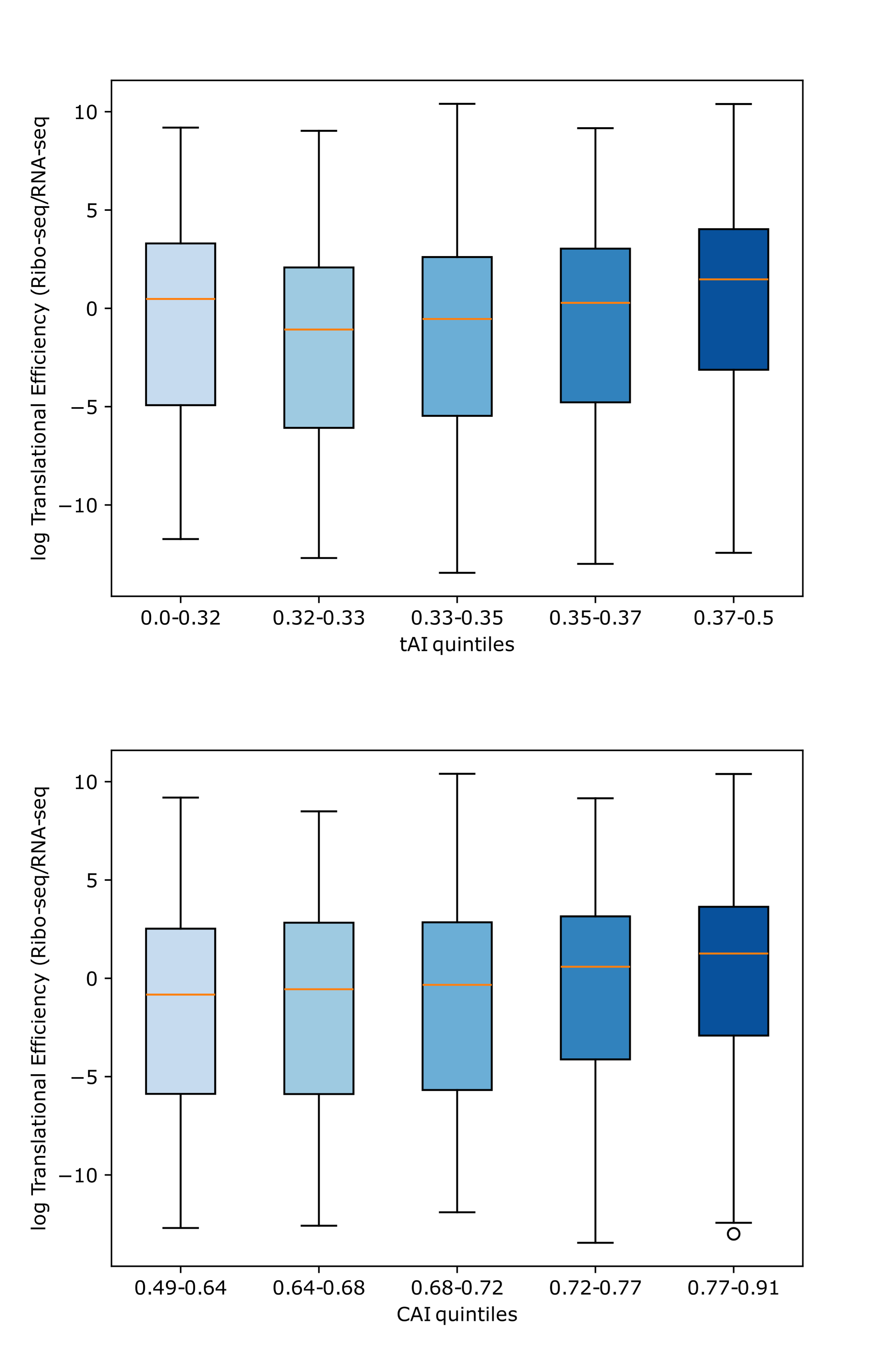

Supplement: S37 Fig — (PNG) [file pcbi.1014501.s037.png]
