# Supplementary material for: Position-dependent codon occurrence is associated with patterns of translational efficiency and ribosomal occupancy in the human transcriptome
Source: PLoS Comput Biol. 2026 Jul 20;22(7):e1014501. doi: 10.1371/journal.pcbi.1014501 (PMC13399515; doi:10.1371/journal.pcbi.1014501)
Supplement: Attachment — Submitted filename: review_PCOMPBIOL-D-23-01868.docx [file pcbi.1014501.s042.docx]

In this article, Subramanian et al. systematically compute and analyze the PDCUB across the whole human transcriptome. They use this metric to predict starting AUG codons, identify enriched and depleted functional groups, and characterize the translational dynamics at the beginning of coding sequences. They compare this metric with CAI and tAI, as well as with empirical datasets (protein to RNA ratios and ribosome profiling).

I found the article solid and exhaustive, with very clear and thorough methodologies and analyses. I believe it is relevant and publishable in this journal. However, the article could benefit from some additional clarifications. Major comments:

- There are many metrics of codon usage out there (which have already been reviewed extensively, e.g. 10.1016/j.csbj.2021.04.042). When proposing the PDCUB for analyzing human protein synthesis, it would be useful to show the correlation of this and other metrics (tAI, CAI, basic CUB without position-dependence) with experimental genome-wide measurements of mRNA translation (protein to mRNA ratios, or Ribo-seq/RNA-seq ratios, aka Translation Efficiency).

- The authors characterize the translation dynamics in the first 300 nt, and they use the tAI for that purpose. Here there are three comments: (a) Why was the tAI preferred over the PDCUB to show profiles? (b) The tAI depends on the modeling of wobble base pairings, whose affinities are captured by the S_ij_ scores. However, these scores were developed for *S. cerevisiae*. Improved scores have been developed for other species (10.1093/dnares/dsu017, 10.3389/fmolb.2023.1218518). (c) The analysis is done by comparing differences between quintiles of PDCUB, but a more systematic analysis by clustering or decomposing recurrent patterns of translation dynamics across human transcripts would be very interesting and add beyond what has already been described.

- The PDCUB contains information both on the amino acid usage and the synonymous codon usage. It would be clarifying to disentangle both effects, since only the second will be applicable to e.g. biotechnological applications to optimize sequences.

- When analyzing the ribosome profiling data, an offset of 15nt from the 5’ end of reads is used to determine the A sites. This offset can nevertheless be variable depending on read length, which should be considered. Specific softwares have also been developed for that goal (10.1016/j.cels.2017.12.007, 10.1371/journal.pcbi.1006169).

- Supplementary table 1 was not included, which could not be reviewed.

Minor comments:

- In the GO analysis, several tissue-specific functions are detected. It would be interesting to directly analyze enrichments of known tissue-specific protein sets (10.1186/s13059-023-02868-2) or proliferation/differentiation sets, which have profound differences in codon usage (10.1016/j.cell.2014.08.011, 10.1016/j.cels.2023.02.002).

- Titles of results sections and figures are often short and scarcely describing the main outcomes of the sections.

- Fig 1A: colors used in clustering are not colorblind-friendly. Also, it is not clear how many sequences are used to compute the z-score of each of the 50 bins of the heatmap. If this number changes per bin, the *n* should be provided.

- Sup Fig 2: the x axis should be the Hamming distance between each pair of codons.

- Sup Fig 3: 3D visualization is confusing, might benefit from faceting 2D plots.

- Sup Fig 5: only the shuffled data is shown, but the same figure with amino acid z-scores should also be included.

- Fig 2C: The highest IC point is around 0.35 bits, but histogram ends at 0.14.

- The performance of the start codon model is often called recall, accuracy, “fraction of high scoring AUGs”, “start codon identification rate”. It would be clearer if the same term is used throughout the text.

- Fig 3A: It is not clear what the values of the x axis mean (kb?).

- The number and size of bins are the only trainable parameters, and defined at the beginning at 3nt, 100 bins. However, during the cross-species analysis, the authors mention that the model was trained for each species. What did this training involve?

- Sup Fig 12: Please indicate what the blue color represents in the legend.

- Sup Fig 14: is it showing the SignalP and PrediSi scores? Maybe it would benefit from a boxplot representation.

- Sup Fig 19: Ratios and differences use very different scales, it would be better to add a secondary y axis or log2 transform ratios.

- Sup Fig 21-22: It is not clear how the trend of translation initiation ramp is defined.
