# Supplementary material for: Position-dependent codon occurrence is associated with patterns of translational efficiency and ribosomal occupancy in the human transcriptome
Source: PLoS Comput Biol. 2026 Jul 20;22(7):e1014501. doi: 10.1371/journal.pcbi.1014501 (PMC13399515; doi:10.1371/journal.pcbi.1014501)
Supplement: Attachment — Submitted filename: review_PCOMPBIOL-D-23-01868_2.docx [file pcbi.1014501.s044.docx]

We thank for the reviewers for their feedback on our manuscript, and we have made an effort to address these and make changes in the manuscript to address these comments. This manuscript is the work of two undergraduates and a master’s student who have all graduated, and over the past year, I have become a father and am raising a 10-month old daughter, all making the process difficult. Nevertheless, I have done my best to respond to these comments during this busy time. We kindly an opportunity for a second revision if our updates are not satisfactory to the reviewers.

This being said, we have made an effort to address these comments and set a better tone of what PDCUB is, and that it does not replace the other ideas, such as translational ramp, or mRNA folding, but may serve as a tool to shed light on these other effects.

Reviewer's Responses to Questions

**Comments to the Authors:
Please note here if the review is uploaded as an attachment.**

**Reviewer #1:**

In this article, Subramanian et al. systematically compute and analyze the PDCUB across the whole human transcriptome. They use this metric to predict starting AUG codons, identify enriched and depleted functional groups, and characterize the translational dynamics at the beginning of coding sequences. They compare this metric with CAI and tAI, as well as with empirical datasets (protein to RNA ratios and ribosome profiling).

I found the article solid and exhaustive, with very clear and thorough methodologies and analyses. I believe it is relevant and publishable in this journal. However, the article could benefit from some additional clarifications. Major comments:

- There are many metrics of codon usage out there (which have already been reviewed extensively, e.g. 10.1016/j.csbj.2021.04.042). When proposing the PDCUB for analyzing human protein synthesis, it would be useful to show the correlation of this and other metrics (tAI, CAI, basic CUB without position-dependence) with experimental genome-wide measurements of mRNA translation (protein to mRNA ratios, or Ribo-seq/RNA-seq ratios, aka Translation Efficiency).

>>We appreciate this suggestion, and we agree of the importance to comparing to other metrics and with experimental data. For this reason, we have included a correlation of CAI in Supplementary Figure 22 and 23, comparisons with tAI (Supplementary Figure 20 and 21). We have also compared to protein to mRNA ratios and found no significant correlation. We therefore hypothesize that the PDCUB pattern has more to do with translation initiation, which is why we observe lower ribosomal occupancy at that position and GO terms that require rapid translation/precise timing of expression such as developmental genes and immune response.

- The authors characterize the translation dynamics in the first 300 nt, and they use the tAI for that purpose. Here there are three comments: (a) Why was the tAI preferred over the PDCUB to show profiles? (b) The tAI depends on the modeling of wobble base pairings, whose affinities are captured by the S_ij_ scores. However, these scores were developed for *S. cerevisiae*. Improved scores have been developed for other species (10.1093/dnares/dsu017, 10.3389/fmolb.2023.1218518). (c) The analysis is done by comparing differences between quintiles of PDCUB, but a more systematic analysis by clustering or decomposing recurrent patterns of translation dynamics across human transcripts would be very interesting and add beyond what has already been described.

>>Thank you for these points. For point a, the tAI was shown here in comparison with PDCUB because PDCUB itself cannot be viewed as a profile. PDCUB is a score that selects for a specific profile of codon usage for the codon sequence, resulting in a singe value. The observation here is that tAI profiles, which as you know can indicate a translational ramp, show the greatest ramp effect when examining the highest PDCUB scores specifically. We were surprised to see this striking correlation. We were struck to see that PDCUB, a score that describes position dependent usage for all coding sequences in aggregate, can produce a spectrum of scores for this preferred codon usage, and to then observe that what has been called a “translational ramp”, and is a feature of examining tAI over the coding sequences, shows a consistent pattern that matches the PDCUB spectrum.

For point b, we agree to the point that using human specific tAI values is necessary. For this reason, we used data computed from human tRNA data that are provided in supplementary table 1, of Tuller 2010 (reference 22 in References). These are human-specific tAI values. Given that the Tuller paper is a landmark paper on this subject in Cell, we thought it prudent to use this, and had questions about the genetic algorithm used in the paper you cited. Our recollection is that a previous reviewer had even suggested to use the Tuller paper. This being said, we do not expect a significant difference with the updated data that was published after our PDCUB project began.

For point c, we agree on this point and would be excited to do that analysis. This would be more of a de novo approach and may turn up some interesting findings. Our approach was to investigate what the primary signal when we align all coding regions, and hypothesize that the aggregate trend represents conditions that are favorable, as this rationale applies to other similar models, e.g. the highest position weight matrix score corresponds to modeling the higher affinity binding sites. All this being said, to address this, we have added this point to the discussion as a proposal for future work, and we would hope be satisfactory to save this for future work.

- The PDCUB contains information both on the amino acid usage and the synonymous codon usage. It would be clarifying to disentangle both effects, since only the second will be applicable to e.g. biotechnological applications to optimize sequences.

>>We agree of the need to disentangle the effects of amino acid usage and synonymous codon usage. We also appreciate the point that for biotechnology applications the desired/encoded protein sequence is established/provided as input and the nucleotide sequence should encode this specific protein. To address this, we investigated the effect of shuffling the nucleotide sequence such that it preserves the encoded amino acid sequence. We call this “synonymous shuffling”. We found that specific codons are enriched compared to the synonymously shuffled sequences (Fig 1B). Indeed, certain specific codons for the same amino acid are favored over others. When creating a sequence to encode a specific protein, one can still optimize PDCUB by selecting codons that encode the sequence, and this effect is mostly in the third position of the codons, which of course is often degenerate with respect to the encoded sequence. This analysis, and the result that PDAAUB does not identify start codons as well as PDCUB, show that the sequence features that are the hallmark of codon sequences are part of codon usage rather than amino acid usage patterns.

- When analyzing the ribosome profiling data, an offset of 15nt from the 5’ end of reads is used to determine the A sites. This offset can nevertheless be variable depending on read length, which should be considered. Specific softwares have also been developed for that goal (10.1016/j.cels.2017.12.007, 10.1371/journal.pcbi.1006169).

>>We agree that is important to select a good offset from the 5’ end. We did experiment with some different offsets, and did not see any meaningful difference. We therefore went with the standard shift. This was about half of the read length in this case, so it made some sense.

- Supplementary table 1 was not included, which could not be reviewed.

>>We do not know how or why this happened. We have taken steps to make sure that this is included, but it is possible that due to an oversight, it was left out. We will double check on the resubmission and expect it to be there.

Minor comments:

- In the GO analysis, several tissue-specific functions are detected. It would be interesting to directly analyze enrichments of known tissue-specific protein sets (10.1186/s13059-023-02868-2) or proliferation/differentiation sets, which have profound differences in codon usage (10.1016/j.cell.2014.08.011, 10.1016/j.cels.2023.02.002).

>>This is an interesting idea, but we did not have time to complete this one. Given that is provided as a minor comment, we decided to leave this for future work. To address this comment, we have added this point to the discussion.

- Titles of results sections and figures are often short and scarcely describing the main outcomes of the sections.

>>Thank you for pointing this out. To address this, we have enhanced the section titles throughout the manuscript, particularly in Results.

- Fig 1A: colors used in clustering are not colorblind-friendly. Also, it is not clear how many sequences are used to compute the z-score of each of the 50 bins of the heatmap. If this number changes per bin, the *n* should be provided.

>>Thank you for this point. We have changed to dendrogram to be color-blind safe. We believe that the heatmap itself is color-blind safe. Furthermore, we agree that it should be noted how many sequences are used for the heatmap and we have added this to the caption. In this figure, we require a valid CDS, and the number of sequences at a given length range from 23,074 to 2,152.

- Sup Fig 2: the x axis should be the Hamming distance between each pair of codons.

>>There may be some confusion on this plot. We have edited the caption to make this more clear, but the x-axis is not edit distance between each pair of codons. The x-axis is the number of Gs or Cs in the codon group.

- Sup Fig 3: 3D visualization is confusing, might benefit from faceting 2D plots.

>>We agree that this 3D visualization is confusing. In earlier drafts, we had this figure as Figure 1B, but moved it to the Supplement in place of a better Figure 2B. However, the same information of this 3D plot is shown in a heatmap, in Figure 1A, but organized more explicitly by GC groupings. The dendrogram in Figure 1A inspired this 3D plot.

- Sup Fig 5: only the shuffled data is shown, but the same figure with amino acid z-scores should also be included.

>>We believe that this supplementary figure to have an incorrect caption. The caption looks like it was copied from Supplementary Figure 4, and was edited, but not finished. This is because it cannot be shuffled by codons and show amino acid heatmap. This is not possible because the amino acid frequencies would be unchanged upon such a shuffling. Therefore, this figure is just what you requested, and only has the amino acid z-scores and no shuffling. The caption has been corrected.

- Fig 2C: The highest IC point is around 0.35 bits, but histogram ends at 0.14.

>>Thank you for pointing this out. The x-axis range has been truncated to only include the values corresponding to the PDCUB pattern. The single value at 0.35 corresponds to the first position. Nevertheless, we have added a note in the caption to make clear that this single value was excluded to make the content of the inset figure visible.

- The performance of the start codon model is often called recall, accuracy, “fraction of high scoring AUGs”, “start codon identification rate”. It would be clearer if the same term is used throughout the text.

>>We agree that this can be confusing. All of these terms mean the same thing, and we expect that the variety used was just to make things more interesting but agree that it is also a source of confusion. To address this, we have changed to use the same terminology throughout.

- Fig 3A: It is not clear what the values of the x axis mean (kb?).

>>We agree that the intended meaning of 1000nt, or 1000 nucleotides should just be kb and have changed all figures for this.

- The number and size of bins are the only trainable parameters, and defined at the beginning at 3nt, 100 bins. However, during the cross-species analysis, the authors mention that the model was trained for each species. What did this training involve?

>>For the cross-species analysis, we used the same parameters identified for human. The signal preferring single-codon bins was so strong, we expect it to not change if mouse has PDCUB. Future work could systematically examine the best parameters of defining PDCUB in different species.

- Sup Fig 12: Please indicate what the blue color represents in the legend.

>>These figures show overlayed histograms comparing signal peptide scores for significant PDCUB compared to background. The captions have been updated to clarify this. Also, it is possible that you are referring to the purplish color that takes up most of the histogram, and this is the overlap of the two semi-transparent histograms.

- Sup Fig 14: is it showing the SignalP and PrediSi scores? Maybe it would benefit from a boxplot representation.

>>We agree that this would be a better representation, but did not have time to complete this change. If given the opportunity for another revision, we will be able to do this.

- Sup Fig 19: Ratios and differences use very different scales, it would be better to add a secondary y axis or log2 transform ratios.

>>We agree that this would be better, but to resolve this issue easiest we opted to remove the differences as the ratio is much more clear in this instance I think.

- Sup Fig 21-22: It is not clear how the trend of translation initiation ramp is defined.

>>We are unclear on this comment as there is no mention of a translation initiation ramp in this figure. The correlations here are not great, but they shift according to different PDCUB quintiles is the main idea for these figures. The window of 30 codons was selected according to the region of maximum change in the tAI plots. Apologies if we are missing something here.

**Reviewer #2:**

Subramanian, Waugh and colleagues describe an analysis of position-dependent codon usage patterns in human genes, using a new metric they call PDCUB. Position-dependent codon usage bias is a well known property of many genomes, and it has multiple possible causes including selection for RNA secondary structure, translational ramps, and nucleotide composition of regions around gene promoters. Despite this, there are no widely accepted measures of position-dependent codon bias and little is known about how this type of bias correlates with functional properties of human genes. This study aims to address this important knowledge gap and provides a number of observations on the correlation of PDCUB with translation, codon adaptation measures and gene ontology classification of genes. Although the results are interesting, I found parts of the paper difficult to follow and I recommend these parts to be thoroughly rewritten before publication.

Major concerns:

1. The section "Optimizing PDCUB weight matrix" unnecessarily complicates what you are trying to do. If I understood this correctly, PDCUB is a simple score based on a match between the sequence of an arbitrary 100-codon window of a gene and a PSSM derived from the empirical frequencies of codons in a 100-codon window downstream of the start codon of human genes? If so, this information is difficult to extract from the manuscript, which instead focuses on describing a convoluted method to predict the start codon of a gene, a task that can be trivially achieved simply by searching for the first ATG substring. I suggest replacing this section with a definition of the PDCUB score, explained in a way that will be accessible to your readers. Once the PDCUB score has been defined, you can then use it to predict start codons and other properties of genes in subsequent sections.

>>Thank you for pointing this out, and we think there are some valuable takeaways here. First, we agree that it is not a difficult task to get high accuracy of start codon prediction by picking the first ATG triplet. However, this is not the point. The point is that we are trying to learn sequence features that discriminate the start codon from the many other ATGs within mRNAs. The “start codon prediction task” is a way of showing that we have extracted meaningful signals associate with real start codons because the PDCUB weight matrix is able to identify them purely on the sequence that comes after it. To address this point, we have clarified that selecting the first AUG would solve this prediction task, and reframed it a little better as we are trying to validate that the models have correctly extracted the sequence features that help define the coding signal.

2. The current PDCUB metric seems to conflate codon usage and amino acid usage. Other methods to quantify CUB isolate the effects of codon usage by calculating frequencies of codons relative to other codons for the same amino acid. If PDCUB indeed represents a combination of amino acid usage and codon usage, this should be stated explicitly and prominently in order not to confuse the readers. It might be better to change the name of the metric to avoid confusion.

>>We agree that it is important to not conflate codon usage and amino acid usage. For this reason, we have performed several controls and additional analysis to uncouple codon usage patterns from amino acid usage patterns. We compared to only using amino acids with PDAAUB, and we have also compared to synonymous shuffling of the coding sequence, whereby we preserve the amino acid sequence but alter the codons that encode it. These two analyses uncouple these effects, but do show that PDAAUB does have some value, but when compared to the shuffling data, we show that this effect is not inherent in the amino acids, but rather in typically one specific codon for that amino acid.

3. The correlations of PDCUB with GO terms and signal peptides could simply represent the differences in amino acid sequences of genes in GO categories or genes with and without signal peptides. To confirm that the results reflect differences in codon usage bias, you should verify that the same patterns are not observed when applying the PDAAUB metric to the GO and signal peptide classification tasks.

>>To address this, we performed a GO enrichment analysis with the set of genes that are predicted to have a signal peptide match with the same web tool. Although there is some overlap as expected, by and large, the groups of enriched GO terms are quite different. Many GO terms that are enriched in the signal peptide group are depleted in the PDCUB set, and vice versa, such as “immune response”.

4. The entire description of PDCUB calculation in the methods section should be revisited, the sentence that starts with "bins b of size w" is particularly unclear.

>>We have attempted to make this clearer, although it is not obvious how to make this more clear. The bins are referred to in the equation by the variable b, and the common way of describing such as thing is to say the “for each bin b” etc when describing and equation. We use of the equation font when referring to each variable in the text to distinguish it from the text.

Minor comments:

- In Fig 1B, it would make more sense to invert the colours by displaying the (original - shuffled) scores, rather than as done currently, (shuffled - original).

>>We have made this change, and now Figure 1B shows original – shuffled. We interpret this difference as the change due to “unshuffling”, going from the shuffled to the unshuffled based on the “final minus initial” interpretation of the difference. But higher terms correspond to more enrichment, which we agree could make more sense to the reader.

- In Figure 3A, all models have a very poor predictive power below 1% - perhaps a problem with axis labeling?

>>This is a good catch. The figure was displaying identification rate as a fraction rather than as a percent. To address this, we have changed the axis labels values to a percentage (e.g. 0.5 -> 50%).

- Figure S3 makes it difficult to see the pattern: can this be displayed in the same format as Fig 1?

>>This figure shows essentially the same information as Figure 1, but in 3D. It isn’t a great figure, but the dendrogram in Figure 1A correspond nicely to the groupings in Supp Fig 3. It isn’t perfect, but it is where the idea came from. But we believe that the same idea can be gleaned from Figure 1A.

- p13, it is not unexpected that PDCUB outperforms both PDAAUB and GC3 models given that PDCUB incorporates both amino acid and codon bias information. Would it be possible to check how PDCUB compares with a combined model that includes PDAAUB and GC3?

>>We did not have time to complete this suggestion, but it is a good suggestion. PDAAUB is the second-best method and does capture a lot of the prediction rate. But with 20 amino acids to choose from, and 61 codons to choose from, the information gain in a codon pattern is higher when considering the log2(61) > log2(20). The PDAAUB does largely match the synonymously shuffled heatmap, suggesting that the informative amino acids have enriched codons that represent them, but because it is only one codon (or two) and always the codon with the GC3 signal, it is plausible that the PDCUB signal is a combination of the PDAAUB and the GC3 signal, but PDCUB is a more succinct way to describe this pattern, as at the end of the day it is a codon signal due to specific codons.

**Reviewer #3:**

The authors present a novel position dependent codon usage bias (PDCUB) score which is based on the distribution of the first 100 codons of genes. They then study PDCUB and among others they show that it can be used to predict translation-initiating codons with greater accuracy than other models. They report relation to position specific GC content, signal peptide, and more. They suggest that PDCUB defines a spectrum of translational efficiency profiles of tAI. An inverse correlation was found between PDCUB score and ribosomal occupancy in the early transcript. They also examine the relationship between PDCUB intensity and functional enrichment.
This is a potentially interesting study, but additional analysis is required; in addition, the authors should better review and discuss previous studies in the field to better understand the results and their novelty.

1) The study is focused on human genome. I expect such a paper to analyze at least a few organisms from different domains of the tree of life.

>>We agree that this would be a good idea, and to address this we did investigate primates and mouse in one figure, but did not solidify the analysis enough to say anything with confidence. We basically see that PDCUB is conserved in the primates, and gets more dispersed as you move away from the primates. However, as noted by other reviewers, the optimal window of PDCUB could be species-specific, and to do an analysis of the phylogentic scope of PDCUB, it would be a much larger undertaking. We hope that we can leave this beyond the scope of this work. We did do a comparison with mouse

2) There are many important previous studies that should be cited and discussed as they may explain some of the reported results. For example, GC content can be explained by mRNA folding profiles and the beginning of the ORF is populated by many overlapping signals related to AUGs, mRNA folding, AA distribution and more (PMID: 25505165, PMID: 32151272).

>>Thank you for pointing these out. To address this, we have added these references and these points to the discussion and introduction. We agree that the mRNA folding would be the most plausible connection to the PDCUB signal. If the 5’ end is to show greater mRNA folding, the only place where one can add GC nucleotides to favor folding stability is in the third position of codons. However, not all GC3 codons are favored. In the end, it may be difficult to uncouple the mRNA folding and the codon usage bias at the beginning of coding regions, and they may describe the same underlying trend. However, given that the tAI is increased for the high PDCUB transcripts, there appears to be a correlation between the PDCUB pattern and translational efficiency of the codons, which would be distinct from mRNA folding. Furthermore, the lower ribosomal occupancy for high PDCUB codon regions suggest a faster moving ribosome, which is at odds with the coding sequence being folded up in base pairs.

3) Figure 2. It will be helpful to generate a model (e.g. regressor) based on all models or a partial correlation analysis to understand the independent contribution of the PDCUB when controlling for all other models *together*.

>>We did not have time to complete this suggestion, but it is a good idea. Typically combined regression models with multiple features would perform better than individual models. But even if so, the highest information comes from the PDCUB score. We expect that even a regressor model using only PDAAUB and GC3 may be as good as PDCUB, but the shuffling data in Figure 1B shows that the performance of PDAAUB is solely do to one GC3 codon for each of the enriched amino acids.

4) Figure 2C: see comments 2), it is easy to explain this region based on the many signals known to appear there.

>>Please see our response to comment 2. We agree to this point, and we have added this to the discussion. It may be difficult to uncouple these effects, and when nature is evolving mRNA folding patterns, the best “wiggle room” is in the third position of codons, leading to the choice of some GC3 codons. However, not all GC3 codons are used in the beginning, which supports the use of a codon model.

5) Figure 2A. It was not clear to me if you also check different lengths of the region or only checked 100 codons ? if not- you should clearly try to optimize the length of the region (check shorter and longer lengths). I believe that this length is organism specific (see comments 1)).

>>We narrowed in on this region in part due to the increased information content observed in Figure 2C, and by visual inspection of the heatmaps. Furthermore, and in our minds more important, is the fact that our neural network, mRNN, pinpointed this region of 1-200nt downstream of the start codon as most informative for distinguishing mRNAs from lncRNA that led us to this window. We agree that it could be optimized further, but we did try a few lengths and 300nt was the best of what we tried. We also agree that the optimal window would be species-specific. However, performing a species-specific analysis of the optimal windows for PDCUB would be a much greater undertaking. To address this, we have listed this as a limitation of our study.

6) “Even though PDCUB only considers the first 300 nt of the coding sequence, we observed improved performance with shorter transcripts.” Is it related to the fact that shorter transcripts are highly expressed? have less splicing ?

>>Based on the data we have seen, we think that this is partly due to there being fewer AUG triplets to select from in the shorter transcripts, but the PDCUB model still performs much better with a higher improvement on the shorter transcripts compared to the null/random model. To address this question, we have speculation in the discussion that the shorter transcripts need more clear signals to be distinguished as mRNAs from the lncRNAs, which also tend to be shorter.

7) GO term analysis: it will be helpful to understand if the signal of part of it is simply related to the expression levels (e.g. there are terms that tend to includes genes with higher expression and this may be the reason you see the association).

>>Thank you for this question. It is hard to assess “expression” in general, as it is of course tissue- and celltype-specific. We did not see a correlation between PDCUB and protein-to-mRNA ratio based on our analysis of protein and RNA data. We hypothesize that PDCUB has more to do with efficient initiation, rather than overall efficient translation. This is consistent with our discussion on the genes with the highest PDCUB require more rapid expression as dictated by regulation of timing (e.g. during development or immune response). This being said, expression level can mean a lot of things, and it is possible these effects could be tissue-specific, and the analysis would be a very large undertaking to tease apart tissue-specific expression and PDCUB. For this reason, we have decided to address this comment by adding this as a limitation to our study in the second to last paragraph of the discussion.

8) “translational valley” – a pattern some-how similar were reported (for example) for mRNA folding (see point 2) and also PMID: 22050731).

>>This study mentioned is in yeast and E. coli, but we believe there could be some correspondence with human. The distinction here is that we have delineated a spectrum of the effects described in this paper. PDCUB is a score that allows us to separate out this spectrum. The study by Tuller et al 2011 seems to be on yeast and bacteria, and is an aggregate analysis rather than separating out the spectrum. To address this comment, we have added some text to the discussion about the fact that PDCUB could be related to mRNA binding with the caveat that there may be more to it given that only some GC3 codons are enriched. mRNA folding is hard to reconcile with specific codons rather than a greater increase in GC content, which is true, but the effect we observe, in particular Fig 1B show that the trend is around specific codons being enriched and depleted in a specific way. At any rate, further work is needed to disentangle codon usage patterns from mRNA folding. We now note in the discussion that PDCUB does not explain the biochemistry or biophysics of what it is actually doing, and it maybe just only be a useful to for investigating other patterns in this region of the coding sequence that have previously been found.

9) A general comment: each p-value should be positive. See for example: “PDCUB score for the coding transcriptome and found significant correlation between the two parameters (2 ≈ 0.3, = 0.0, Supplementary Figure 20).”.

>>We agree that p-values should be strictly greater than 0. This is a common issue with floating point precision, where a p-value is calculated so low, as it is below the floating point precision for outputting a meaningful number, and this can lead to p-values being computed as 0.0 when in fact they are 10^-40 or some very small value. That being said, I don’t claim to fully understand the inner workings of the algorithm that scipy.stats uses to calculate p-values from correlation data. To address this, we have removed the p-value in this instance as the R^2 is probably more informative.

10) Ribo-seq (e.g. figure 4 D). I expect to see larger differences among the different quantiles. I suggest to check additional dataset and also try to check the average of a few datasets.

>>We agree in the importance of comparing to multiple datasets as a control. For this reason, we performed the same analysis on a dataset from another lab (Supplementary Figure 24) with and with CHX treatment using another dataset (supplementary figure 25) and in a different cell line (HeLa cells shown in Supplementary Figure 26). We see no significant difference between these other datasets. We should clarify that we are showing a relative ribosomal occupancy, where we normalize across the length of the transcript. In this sense, if we sum all the terms of the ribosomal occupancy, it should sum to 1. This is perhaps different, but it gets at the key idea, which is the shape of the ribosomal occupancy pattern, and where the ribosome is spending more time.

11) “Notably, while Tuller and others have examined the features of translational ramps in terms of complete transcriptomes for a given species, we have investigated individual subgroups of human transcripts and found that there is a spectrum of tAI trajectories”..“Previous studies have been conflicted about the presence of a translational ramp in mammals, with some reporting no ramp [28] and others reporting a distinct ramp [22]. We find that both results are true depending on where one looks within the PDCUB spectrum, with the highest-PDCUB transcripts having both a strong initial increase in and steady-state average tAI (translational highway), intermediate PDCUB transcripts having a weaker ramp or no ramp...” note that previous studies claims that some of the genes (e.g. based on expression and function) have ramp (or stronger ramp) and some don’t and an analysis not only based on all genes has been reported. Please read carefully the papers mentioned in 2), 8) and ref. [22]. You should better describe the novelty of your paper (which of course exist).

>>We agree to better describe the novelty of our paper, and we have added more references of prior work. They key novelty that we have described is by using PDCUB score, we are able to separate out a spectrum of transcripts that we think better describes what others have shown before. While other studies have shown more aggregate effects of all transcripts, we show that a score based on the codon usage patterns demonstrates that specific codons are responsible for the position-dependent trends. We made edits in the discussion to make clear that PDCUB is a score, but it does not explain its biological significance, which may be due to things like amino acid usage and/or mRNA folding. However, this score may capture something useful, that can also shed light on the other interesting patterns in this region. Hopefully these edit better set the tone that PDCUB and our score do not replace these other studies
