# Supplementary material for: Position-dependent codon occurrence is associated with patterns of translational efficiency and ribosomal occupancy in the human transcriptome
Source: PLoS Comput Biol. 2026 Jul 20;22(7):e1014501. doi: 10.1371/journal.pcbi.1014501 (PMC13399515; doi:10.1371/journal.pcbi.1014501)
Supplement: Attachment — Submitted filename: reviews.docx [file pcbi.1014501.s045.docx]

We thank the editors for the opportunity to revise our manuscript and for the flexibility on the deadline. We are pleased to report that we have addressed every point and that the revisions have greatly improved our manuscript.

We thank all the reviewers for their thoughtful comments and feedback. We have responded to each point, and have significantly updated our manuscript. We are pleased that the reviewers found our previous revision to be improved, and we acknowledge the weaknesses in our previous revision in regard to many of the reviewers’ comments. However, we feel that by carefully addressing each of the reviewers’ comments, we have substantially improved our manuscript, and feel more confident about the conclusions based on the added controls and investigations that have been recommended.

Because it was requested that we rename the method, in many cases we have recreated the original figures, allowing us to further evaluate the pipeline. Also, because there were requests to compare GO term enrichment, this necessitated that we completely redo the entire GO term enrichment because the database used by the tool that we used has been updated. However, almost no conclusions were changed compared to previous work, except one point about “skin” related function was not observed with the new GO database. We also found differences in GO terms related to signaling, and therefore removed those from the results and discussion. We still observe the same enrichment in terms related to developmental biology and depletion of GO terms related to immune system response.

We address each comment in detail as follows. Because this document has gotten complex, to better delineate this document we have begun our responses in this revision with “>>>>” and the end of each comment section with “<<<<” to help also distinguish quoted text from our previous response to reviewers, which begain with >>. We added this and other details to better distinguish our comments from quoted previous comments and when quoting the reviewers’ previous comments.

**Reviewers' comments:**

Reviewer's Responses to Questions

**Comments to the Authors:
Please note here if the review is uploaded as an attachment.**

Reviewer #1: Thank you for the efforts in addressing the reviews. Most of my comments are now correctly addressed, and I believe the article is now improved. Nonetheless the authors themselves mention several times in their response that some of the reviewers' suggestions (although mostly minor) could not be implemented due to the lack of time. I believe a second revision would grant them the needed time for this.

In particular, my only major concern is the lack of evidence for the statement "PDCUB is Associated with Patterns of Translational Efficiency". The authors only compare PDCUB to estimates of TE like tAI or CAI, but there is no data supporting that PDCUB correlates with protein-to-mRNA ratios or Ribo-seq/RNA-seq ratios (aka Translation
Efficiency). The authors actually mention in the revision that "We have also compared to protein to mRNA ratios and found no significant correlation", and they even suggest that the effect is more on the distribution of ribosome in the transcript rather than in the overall TE. Therefore, the claim that PDCUB is associated with TE is not sufficiently supported. To prove the claim, one would need to show the correlation R2 of PDCUB vs experimental data of TE (either from protein/mRNA or Ribo-seq/RNA-seq). And to grasp how much of this TE variance is better explained by PDCUB compared to other metrics, compare the obtained R2 with "tAI vs TE" and "CAI vs TE".

>>>>We agree with this point, and we thank the reviewer for pointing out this issue with the previous revision. To address this, we have computed TE for all transcripts using Ribo-seq and RNA-seq data from the following paper:

Hia F, Yang SF, Shichino Y, Yoshinaga M, Murakawa Y, Vandenbon A, Fukao A, Fujiwara T, Landthaler M, Natsume T: **Codon bias confers stability to human mRNA s**. *EMBO reports* 2019, **20**(11):e48220.

We have added these data as new Figure 5A and Supplementary Figure 38. Please note that we have changed the name of our score per the request of Reviewer 2 and now call it the POsition-Specific Codon Occurrence (POSCO) score. We found that there was a comparable level of correlation for POSCO vs TE as there was for CAI vs TE and tAI vs TE when grouped into quintiles, using an approach similar to that shown in the above paper. The difference is we used quintiles to be consistent with the rest of our paper. We believe that this figure shows that POSCO is comparable to the baseline provided by CAI and tAI, and therefore conclude that we have the evidence needed to conclude that POSCO is associated with patterns of translational efficiency. In addition, we have compared protein-to-RNA ratios, and included this in Supplementary Figure 34 in response to Reviewer 3, and we believe this also supports this conclusion. The correlation is not as strong, as these data tend to be a bit noisier, but we can see for the highest POSCO, CAI, and tAI scores, the protein-to-RNA ratio is the highest. Notably, the lowest quintile for POSCO has lower protein-to-RNA than the lowest quintile of tAI, suggesting a benefit of POSCO in this regard.

<<<<

**Reviewer #2:**

 The authors seem to have largely ignored comments in my original review, and I do not recommend publication.

>>>>We apologize for any omission in the previous revision, and we seek to address each comment point-by-point, and satisfactorily address each unaddressed point from the previous submission. We believe we have done so and it has improved our manuscript.

<<<<

In the first round of review, I recommended that the authors remove or thoroughly rewrite the section that deals with prediction of start codons. The authors now added some text to this section but this does not address my concerns. I find this part of the manuscript to be of little interest, because (1) we already know where start codons are, and there is no practical benefit in predicting start codons based on codon bias; (2) the ability to predict a start codon from codon bias is not surprising, given the known variation of GC content, amino acid content and codon bias along genes (as shown in previous publications and in Figure 1 of the present mauscript); (3) it is unlikely that codon bias in a 100-codon window mechanistically influences start codon selection. Instead of focusing on the prediction of start codons, it would be better to document the position-dependent codon bias, define the PDCUB metric more clearly, and analyse the possible causes and consequences of PDCUB.

>>>>Thank you for these comments, and we feel that by addressing these points our manuscript has greatly improved in clarity. Firstly, this comment is based on a statement in the original review where the reviewer claimed that the first AUG is typically the start codon by stating that the start codon prediction task is “a task that can be trivially achieved simply by searching for the first ATG substring”, however this assumption is not true. It may be true that in bacteria or other organisms, but it is not true in mammalian transcriptomes. Studies have shown that in fact only around half of transcripts use the first AUG as the start codon in human and rodent genomes:

Calvo S.E., Pagliarini D.J., Mootha V.K. Upstream open reading frames cause widespread reduction of protein expression and are polymorphic among humans. Proceedings of the National Academy of Sciences of the United States of America. 2009;106:7507–7512. doi: 10.1073/pnas.0810916106.

Iacono M., Mignone F., Pesole G. uAUG and uORFs in human and rodent 5′untranslated mRNAs. Gene. 2005;349:97–105. doi: 10.1016/j.gene.2004.11.041.

Matsui M., Yachie N., Okada Y., Saito R., Tomita M. Bioinformatic analysis of post-transcriptional regulation by uORF in human and mouse. FEBS Letters. 2007;581:4184–4188. doi: 10.1016/j.febslet.2007.07.057.

These studies observe upstream AUGs (uAUGs) before the annotated start codon and suggest that this is involved in regulation of translational efficiency. Indeed, we find that the percentage of transcripts with uAUGs is about 48% in GENCODE v34 used in our analysis. Therefore, in a transcriptome with uAUGs, it does raise the question of what sequence and structural features the ribosome would use to identify the start codon.

Secondly, we note that our goal is not to predict start codons, and it is possible that our current revised manuscript or our responses to the previous review did not make this clear. We have renamed this section and thoroughly rewritten the manuscript to remove all mention of “prediction of start codons” or similar phrases accordingly, and have renamed this task as the “match rate” to describe what percentage of transcripts match this pattern for start codons better than elsewhere in the transcript. We were using this task to assess whether the sequence features associated with POSCO (we renamed PDCUB to POSCO in regard to another of your points below) are enriched in start codons compared to elsewhere in the transcript. The reasoning is that it is a defining feature of start codons inasmuch as it does not occur randomly throughout the transcript, or downstream of the start codon after another AUG codon as observed for CAI. We find that computationally the best (and possibly only) way for us to set the length of 100 codons, and the bin size, and to assess to what extent POSCO describes sequence features associated with start codons as measured by the “match rate”.

Thirdly, in the first review, you stated that the window of 100 codons was “arbitrary”, which is not true. In fact, it is based on multiple observations from the current manuscript, such as the z-score heatmaps and including a new Supplementary Figure 7 that we added to systematically determine an optimal model length in regard to a point made by Reviewer 3, which further demonstrates that this window is not arbitrary. Moreover, In our previous studies that demonstrate that a neural network (mRNN) trained to distinguish mRNAs from lncRNAs, and with no prior biological input/knowledge of start codons, ORFs, or amino acids, made its decision around 200nt (~100 to ~300) from the start codon. This work convinced us to further explore this region of the transcriptome specifically, which motivated this manuscript.

Fourthly, you have a hypothesis that the first ~100 codons are unlikely to mechanistically influence start codon selection. It is uknown whether this hypothesis is true, and many of the papers cited above hypothesize that sequence features around the actual codon could help to distinguish real start codons from uAUGs. We are in part motivated by our neural network mentioned above that, without any prior biological knowledge of translation, made its decision in this very region. I know that this is not biological evidence of this fact, but to us, it is a hypothesis that the translational machinery may be influenced by this region as well. One of our goals for the project was to try to understand how, in a transcriptome with uAUGs and lncRNAs with ORFs, does the ribosome identify the start codon of real mRNAs. Furthermore, we show, based on new Supplementary Figure 38 that we created based on comments from Reviewer 3, that high-POSCO transcripts show a stronger local mRNA folding immediately after the start codon, consistent with published data and hypotheses that in fact local folding may halt the scanning by the pre-initiation complex to better recognize the start codon. Therefore, these data add a further layer of interpretation of POSCO in that it is associated with position-specific enrichment of specific codons that increase GC content (particularly GC3) that may facility local folding in this region after the start codon and at the same time while also being codons that are associated with highly available tRNAs (high tAI). This set-up is known prevent ribosomal traffic jams, and lead to rapid movement of the ribosome, despite the local folding as observed in our collective data presented here. Although we have removed “start codon prediction” from our manuscript, we feel that that the data we present support the idea that sequence features associated with POSCO may in fact help the translational machinery identify the start codon.

Fifthly, and importantly, Reviewer 3 wanted us to add additional results related to this task, including a regression model (new Table 1, new Supplementary Figure 11) and further data supporting the choice of 100 codons (new Supplementary Figure 7). We would like to address the comments of every reviewer, and we feel that our solution to rename the task as “match rate” to better reflect our goal and to make clearer that our goal is not to simply predict start codons, we have reached a better compromise for all reviewers.

In summary, to address this point, even though the literature supports the idea that the first AUG is not always the start codon, and many have suggested that sequence features may help the ribosome distinguish start codons from uAUGs, we have significantly rewritten these sections using different terminology to better articulate the goal of this work. Our goal is to assess whether and to what extent that the sequence features encapsulated within the POSCO score consistently reflect those of start codons. Therefore, we use the term “POSCO match rate” to better describe this goal. We use the rate at which the start codon has a highest POSCO score compared to other AUGs as a way to assess whether or not POSCO is a good description for the position-dependent codon trends observed and as a way to optimize the length and bin size that we use to define our POSCO score because there is no other good way to do this. Finally, our data and the literature are consistent with the notation that the sequence features delineated by the POSCO score are associated with 1) increased mRNA folding after the start codon, which helps the pre-initiation complex identify the start codon and 2) utilizes highly available tRNAs (high tAI) resulting in overall improved translational efficiency.

<<<<

In answer to my major comments 2 and 3, the rebuttal letter reiterates some points made in the original manuscript, but it is not clear if any new analyses were made or if changes were introduced in the manuscript in response to these comments. It still seems that a good part of the observations reported in the manuscript might be caused by patterns of amino acid usage along proteins.

>>>>Thank you for returning to these important points and we feel that by addressing these points our manuscript is substantially improved. There are several points here, so we will carefully address each one as follows. For major comment 2, the original comment was:

“2. The current PDCUB metric seems to conflate codon usage and amino acid usage. Other methods to quantify CUB isolate the effects of codon usage by calculating frequencies of codons relative to other codons for the same amino acid. If PDCUB indeed represents a combination of amino acid usage and codon usage, this should be stated explicitly and prominently in order not to confuse the readers. It might be better to change the name of the metric to avoid confusion.”.

Upon revisiting this comment, we have a better appreciation for this point. The point as we understand it is that other metrics of CUB such as CAI measure the contribution of a codon relative to other synonymous codons, and this “relative to synonymous” part is what defines codon usage bias as a term. To avoid this problem, we will change the name of our codon PSSM score to POsition-Specific Codon Occurrence (POSCO) score. Furthermore, we have stated clearly in the last sentence of the Introduction section and the second sentence of the Discussion that our model unlike CAI is not relative to synonymous usage directly in the score, but rather relative to the average probability of the same codon over all positions. This being updated, we do believe that the heatmap presented in Figure 1B does reveal some interesting patterns of synonymous codon usage, hence the PDCUB phenomena, where codons enriched for blue do show preferential usage of synonymous codons restricted to the first 100 codons (side note, this is further motivation for the 100 codon window) but also we recognize that this heatmap is distinct from our POSCO PSSM score, and will rename it to avoid confusion. Future work could define a score that builds into it the position-dependent codon usage that is relative to synonymous codons.

The original comment 3 was:

“3. The correlations of PDCUB with GO terms and signal peptides could simply represent the differences in amino acid sequences of genes in GO categories or genes with and without signal peptides. To confirm that the results reflect differences in codon usage bias, you should verify that the same patterns are not observed when applying the PDAAUB metric to the GO and signal peptide classification tasks.”

To address this, we now include new some figures regarding this in the Supplementary materials. We had to redo all GO Term analysis with an updated 2025 database to make things consistent for this new analysis, and this had some changes that have been updated. For Signal Peptides, we did an additional GO term analyses using high POSCO transcripts without signal peptides in comparison to our original list (new Supplementary Figure 21 and new Supplementary Table 3). We note that the majority of GO terms and all the GO terms discussed in the results persist even when genes with Signal Peptides are removed. This includes an enrichment of developmental genes for brain, heart, skeletal, and many other systems. In addition, the noted depleted terms persist even when genes encoding signal peptides are removed. In fact, as it turns out we found that genes with signal peptides are enriched for immune system and detection of chemical stimulus and smell related GO terms, while POSCO is depleted. However, upon redoing the GO term analysis we didn’t see as clear a trend in “Signaling and Transport” related terms, and have removed those sections. Any other statement about GO term enrichment or depletion that was not found in the updated 2025 analysis was removed. It’s fair to say that signal peptide encoding transcripts do not have the same GO Terms as high POSCO transcripts, quite the opposite. In some cases, we do observe some “biological development” related GO terms to be enriched in the signal peptides, but these are related to different systems than the ones we have noted.

In addition, your question about the signal peptides further motivated us to do additional controls. We first created a codon z-score heatmap for all transcripts with signal-peptide-encoding transcripts removed (new Supplementary Figure 22). We found that other than a reduction in leucine codons, which are known to be enriched in signal peptides, the heatmap was largely unchanged. Moreover, we tested whether the POSCO score created from transcripts with signal peptides removed would change the observed “match rate”, and found that it only slightly decreased the score, which remained a higher match rate compared to all other scores (Supplementary Figure 23). All these data together support that although signal peptides may play a small part, it does not explain what is going on with the POSCO score.

Next, we addressed your point about PDAAUB, which have now renamed to “AAPSSM” in regard to your previous point to avoid confusion since we no longer use the term PDCUB to describe our score. We found that the distribution of AAPSSM scores is much narrower than POSCO scores, and shows a greater overlap with the background distribution, which results in fewer significant AAPSSM transcripts at an FDR of 0.05 (new Supplementary Figure 24). This could be in part due to the fact that in many cases codons for the same amino acid cancel each other out. For example, valine has both 5’ enriched and 5’ depleted codons but is not enriched or depleted at the amino acid level. POSCO allows for greater delineation, and hence a wider range of scores. AAPSSM for the most part does not account for variation in GC content observed at the 5’ end. Furthermore, we found that most of the enriched and depleted GO terms after we examine GO terms for significant POSCO transcripts after significant AAPSSM genes are removed (new Supplementary Figure 25 and new Supplementary Table 4). Although there are some changes in significant GO terms, all notable GO terms previously discussed remain and no conclusions were changed about enriched function.

<<<<

Minor comment:

Figure 3B - there are errors in the figure legend: "Frame 1 triplet" is repeated three times instead of Frame 1, 2 ,and 3; and the dashed line that indictaes the start codon is blue in the legend but green in the plot.

>>>>Thank you for catching this error; It has been fixed.

<<<<

Reviewer #3: The paper was improved but still there is a lot of work (I am spending a lot of time on this review and hope that the authors will take my comments seriously and update the paper comprehensively):
1) My prev. comment 1: I do think that you should add at least one additional organism. If this is so challenging it may suggest that the methodology & conclusions are problematic and cannot be generalized. If there are differences among other organisms this is clearly interesting and should be reported.

>>>>Thank the reviewer for their comments and we do take the comments seriously and hope to carefully address them. We feel we have carefully addressed this point in our latest revision and it has improved our manuscript. To address the comparisons with other species, we examined POSCO scores (Please note, we have renamed the model to POsition-Specific Codon Occurrence (POSCO) score in regard to a comment from Reviewer 2) in single-copy orthologs across primates and mouse (new Supplementary Figure 12). We observed that while POSCO scores are largely conserved and unchanged in the higher apes (chimps, orangutan, gorilla), it is highly diverged in other primates (macaque and mouse lemur) and even more diverged in mouse. This effect is quantified by the R^2^ value in the legend. We further examined mouse POSCO to describe how it was so different from humans in new Supplementary Figures. First, we found that the codon z-score heatmaps were strikingly similar to that of human, with some amount of additional noise and differences (new Supplementary Figure 13 and 14). We found that the region of enrichment extends further in mouse, and there are some sporadic points of enrichment in mouse. We next updated what is now Supplementary Figure 15 and found comparable match rates for human POSCO scores applied to mouse, and mouse POSCO scores applied to human compared to human-to-human and mouse-to-mouse sets. We redid this analysis to ensure that both were treated the same using an 80/10/10 train/test/validation split and computed the POSCO score on the training set and evaluated on the test set and to double check the pipelines. In conclusion, we show that the POSCO scores are conserved among the primates, but more diverged for individual genes in mouse. However, overall mouse shows similar aggregate statistical patterns of the codon z-score heatmap. While individual genes are different in POSCO compared to humans, collectively they are similar.

<<<<

2) My prev. comment 2: I must say that your answer is not 100% clear to me (see comments below). In any case, you should add a clear discussion about this point with relevant references to the discussion section.

. We agree that the mRNA folding would be the most plausible connection to the PDCUB signal. If the 5’ end is to show greater mRNA folding, the only place where one can add GC nucleotides to favor folding stability is in the third position of codons. However, not all GC3 codons are favored. In the end, it may be difficult to uncouple the mRNA folding and the codon usage bias at the beginning of coding regions, and they may describe the same underlying trend.
- there are combinations of constraints on the coding sequence and the fact that not all GC3 codons are favored does not prove/disprove that mRNA folding is not an important variable here. In addition, folding is effected by combination of codons and just looking at GC3 codon is not good enough.

However, given that the tAI is increased for the high PDCUB transcripts, there appears to be a correlation between the PDCUB pattern and translational efficiency of the codons, which would be distinct from mRNA folding.
The correlation is far from perfect; thus, this can clearly explain only part of the signal.
Furthermore, the lower ribosomal occupancy for high PDCUB codon regions suggest a faster moving ribosome, which is at odds with the coding sequence being folded up in base pairs.
The mRNA folding can affect ribosomal density and movement in various ways. It can increase and decrease the density in various ways.

** Note that I mentioned other patterns (other than folding) at the beginning of the ORF. You did not consider this at all.

>>>>Thank you for revisiting this point as it is very important. This question has inspired some further analysis and overall improved our manuscript. First, we have added several points and references to the Introduction and Discussion to better describe prior work in this area, as per your comment in the previous review. Secondly, we performed a new analysis to compare local folding energy in windows relative to the start codon as has been done previously. We found that high POSCO transcripts show much lower, more stable folding energy after the start codon (shown in new Figure 5B). We believe that this is consistent with two points from the literature. As your references noted, it is hypothesized that local folding after the start codon may help halt the scanning by the pre-initiation complex, thereby improving identification of the start codon by the translational machinery. We believe this may be part of the local folding, which may facilitate more efficient initiation. Another point is that local folding after the start codon may reduce traffic jams. This point, the reduced traffic jams, may in part explain why we see reduced ribosomal occupancy around the start codon, because the net effect is more efficient movement despite the ”speed bump”. Moreover, the use of codons associated with highly available tRNAs (high tAI) may further help increase ribosomal speed early on, thereby lowering the occupancy. POSCO seems to capture signals that are associated with increased stability of local mRNA folding and at the same time high tAI codons. A powerful combination that may be help facility the increased translational machinery that we observe in the new Figure 5A. Regarding the other points, amino acid usage is another possible pattern. Apart from translation involving a degenerate code, there is always information lost when translating from codons to amino acids. This limits the utility of amino acids biases for correlating with many other patterns, such as mRNA folding. For example, we show that in some cases, such as valine, there are both enriched and depleted codons, but valine is not enriched at the amino acid level. We have several figures throughout the manuscript showing that amino acid patterns do not explain POSCO, and are not as good as correlating with the features that POSCO correlates with.

<<<<

3) My previous comment 3: The fact that you "did not have time" is not an "excuse". You can ask the editor for an extension and finish all the comments (this one and others). It does not make sense to publish a paper with so many " holes" and problems.

>>>>Thank you for returning to this point up in this revision, and we apologize that we had exceeded our requests for extensions before the previous revision due to personal family matters. For clarification, here is the comment 3 from the previous review:

“3) Figure 2. It will be helpful to generate a model (e.g. regressor) based on all models or a partial correlation analysis to understand the independent contribution of the POSCO when controlling for all other models *together*.”

To address this comment, we have created a logistic regression model with all models combined to assess performance on the POSCO match rate, which are presented in new Supplementary Figure 11 and new Table 1 in the main text. We find that the combined regressor of all models does perform better and produces a higher “match rate”, which is what we have renamed what was previously called “start codon identification” in response to another reviewer’s comment. We used a logistic regression model with all model scores as features. By max-min normalizing all features (scaling from 0 to 1), we can directly compare the weighted contribution of each model. When we combined all models, the highest weights were indeed found for AAPSSM (we have renamed PDAAUB to AAPSSM in response to the update of PDCUB to POSCO requested by another reviewer), while the second highest weight is found for POSCO. However, when we remove the AAPSSM from the combined model, the match rate is almost completely unchanged, and the POSCO model is up weighted to the highest weight, at similar levels to the combined weights of POSCO and AAPSSM in the all-model regressor. To us, these observations to suggest that POSCO provides all the information that AAPSSM does, but these kinds of regression models will split the weights with correlated features. But given that the genetic code is degenerate, there is always a loss of information at the amino acid level compared to the codon level. We think this is why POSCO has a higher match rate on its own compared to all other models on their own including AAPSSM.

Along these same lines, we have provided other related data to this point. First, we show that POSCO shows a greater difference compared to the background distribution (defined by non-start AUGs) than AAPSSM does. When you compare new Supplementary Figure 24 to Supplementary Figure 16, it is clear why POSCO performs better due to less overlap with background and a wider distribution. Furthermore, we find that by removing transcripts from the significant POSCO set that also have significant ASPSSM scores, we do not see a major change in the functional enrichment that we previously noted (new Supplementary Figure 25 and new Supplementary Table 3).

<<<<

4) My previous point 4: please see my comment 2) above.

>>>>Thank you for returning to this important point. For clarification, here is the comment 4 from the previous review:

“4) Figure 2C: see comments 2), it is easy to explain this region based on the many signals known to appear there.”

This comment was regarding Figure 2C, the information content plot. We have added more text to discuss other known signals in this region when presenting these results. This plot is showing position-specific deviations from the global frequency of codons in particular bins relative to the start codon as quantified by the change in Shannon Entropy relative the background. In essence, we look at this as another way to examine the extent to which there are position-specific patterns in codons and collapses the 3D data shown in Supplementary Figure 2 (codon heatmap) into two dimensions. In fact, this information content, defined as the change in entropy, is computed from the same probability matrices used to compute the POSCO matrix. In other words, the information content in Figure 2C is analogous to the height of a sequence logo used to describe sequence motifs but used to describe the POSCO matrix used in our score. I expect that a similar plot made using amino acid frequencies or single-nucleotide frequencies would not have as much information. This is in part because sense codons have the largest number of states, 61 codons compared to 20 amino acids or 4 nucleotides. Certainly, there are many overlapping patterns here, but because this quantification specifically parcels into codons may be meaningful here. There are many different overlapping signals, particularly when we combine frequencies computed from all transcripts into this data. We do show that many of the known signals in this region are correlated with one score, the POSCO score. This includes tAI ramp magnitude and slope, folding energies. We have done our best to address comment 2 in a previous point, and we have included additional figures and discussion and introduction about the other signals. Hopefully this is sufficient, and if not please do let us know what other signals should be discussed in regard to this region.

<<<<

5) My previous point 5: see my comment 1) above. The length is clearly organism specific, and it will be good to show this (it is strange to me that you unable to do this).

>>>>Thank you for this point. For clarification, here is comment 5 from the previous review:

“Figure 2A. It was not clear to me if you also check different lengths of the region or only checked 100 codons ? if not- you should clearly try to optimize the length of the region (check shorter and longer lengths). I believe that this length is organism specific (see comments 1)).”

To address this point, we have included a new Supplementary Figure 7 as a way of checking different lengths for the model to optimize the length of the region. We plotted the match rate as a function of the length of the model, and show that this does keep increasing, but at a lower rate. The length of 100 corresponds to about when the smoothed derivative first approaches 0, and where the derivative flattens out. We also examined mouse, and found in the new Supplementary Figure 13 there are large scale differences with mouse. However, on the zoomed in level, this looks strikingly similar to human (see new Supplementary Figure 14. There are some sporadic points of enrichment, but on the whole, pretty consistent. That said, we completely agree that if we went further out, to Drosophila for example, we would not see the same patterns. We will note this point in the Discussion about the level of conservation of this patten and that future work needs to explore this in more species.

<<<<

6) My previous point 7: Your answer is not clear to me.

>>Thank you for this question. It is hard to assess “expression” in general, as it is of course tissue- and celltype-specific. We did not see a correlation between PDCUB and protein-tomRNA ratio based on our analysis of protein and RNA data.
*did you check relations to protein levels alone and/or mRNA levels along (you can use mean over tissues). If there is not relations it is very strange in my opinion.

We hypothesize that PDCUB has more to do with efficient initiation, rather than overall efficient translation. This is consistent with our discussion on the genes with the highest PDCUB require more rapid expression as dictated by regulation of timing (e.g. during development or immune response).
Efficient initiation usually (almost always, in natural conditions) will have a significant effect on translation.

This being said, expression level can mean a lot of things, and it is possible these effects could be tissuespecific, and the analysis would be a very large undertaking to tease apart tissue-specific expression and PDCUB. For this reason, we have decided to address this comment by adding this as a limitation to our study in the second to last paragraph of the discussion.
You can easily check if there is a relation by comparing to mean expression over all tissues (there is high correlation between the expression levels of genes among different tissues; thus, this is a very good proxy to typical tissue specific expression).

>>>>Thank you for raising this comment from the last review. To address this, we have compared protein-to-RNA ratios based on published data and using the average expression across multiple tissues (new Supplementary Figure 34). We compared two studies mentioned previously in our paper, with protein GCMS data and RNA-seq for different tissues. We have significantly revamped the pipeline regarding protein and RNA expression and removed some previous supplementary figures on this that did effectively connect with the rest of the manuscript. In replacement, we have also included these figures, and the histogram of the log protein-to-RNA rations (new Supplementary Figure 33). We used quintiles to show this similar to other analyses in the paper. We find that POSCO has a comparable enriched protein-to-RNA ratio for the highest POSCO genes similar to tAI and CAI, but also show a lower protein-to-RNA ratio for the lowest quintile that tAI. Furthermore, we have also compared Ribo-seq to RNA-seq TE ratios on data based on Hia 2019 paper in response to Reviewer 1 and presented in a new Figure 5A and Supplementary Figure 38. We show a comparable correlation with CAI and tAI. Taken together, these data support the idea that POSCO scores are associated with higher translational efficiency. In relation the the mRNA folding point from above, this further sheds light on the potential for RNA folding to play a role connected with all other data, it supports the idea of reducing traffic jams in light of the reduced ribosomal occupancy data for high POSCO transcripts. We think the location the increased folding also supports the idea of halting pre-initiation complex scanning. All of these points have been expanded upon in the Intro, Results, and Discussion.

7) My previous comment 9: it is very important to add a p-value. You should write for example p<10^(-40) if this is your resolution.

>>>>Thank you for this clarification. Floating point precision 10^-308 for python, but this may be a bit extreme and we have taken your suggestion of p<10^-40.

8) My previous comment 11: you still do not describe accurately the previous studies that have reported scores for single genes and groups of genes (contrary to what you wrote).

>>>>Thank you for returning to this point as it is important. For clarity, the comment 11 from the previous revision was:

“11) “Notably, while Tuller and others have examined the features of translational ramps in terms of complete transcriptomes for a given species, we have investigated individual subgroups of human transcripts and found that there is a spectrum of tAI trajectories”..“Previous studies have been conflicted about the presence of a translational ramp in mammals, with some reporting no ramp [28] and others reporting a distinct ramp [22]. We find that both results are true depending on where one looks within the PDCUB spectrum, with the highest-PDCUB transcripts having both a strong initial increase in and steady-state average tAI (translational highway), intermediate PDCUB transcripts having a weaker ramp or no ramp...” note that previous studies claims that some of the genes (e.g. based on expression and function) have ramp (or stronger ramp) and some don’t and an analysis not only based on all genes has been reported. Please read carefully the papers mentioned in 2), 8) and ref. [22]. You should better describe the novelty of your paper (which of course exist).”

We are pleased to read that you feel that novelty exists in our manuscript. We have carefully read all of these papers and did our best to better articulate our findings in the context of this prior work to better characterize what is novel about our manuscript.

We do recognize that this paper here (PMID 22050731):

<https://genomebiology.biomedcentral.com/articles/10.1186/gb-2011-12-11-r110>

does indeed examine different groups of genes, and show that, based on a stratification by ribosomal density, high-ribosome density genes are associated with more stable local folding energy (which would be linked to higher GC-content) and local tAI. As such, we have removed any claims of novelty regarding partitioning into groups, and have also removed language that sounds like we are clamining to “coin a term”, which we agree sounds bad. We have reworded to just state that it is a way of describing what we are observing and did a better job to describe the work that has been done related to this work.

**Have the authors made all data and (if applicable) computational code underlying the findings in their manuscript fully available?**
The [PLOS Data policy](https://journals.plos.org/ploscompbiol/s/materials-and-software-sharing) requires authors to make all data and code underlying the findings described in their manuscript fully available without restriction, with rare exception (please refer to the Data Availability Statement in the manuscript PDF file). The data and code should be provided as part of the manuscript or its supporting information, or deposited to a public repository. For example, in addition to summary statistics, the data points behind means, medians and variance measures should be available. If there are restrictions on publicly sharing data or code —e.g. participant privacy or use of data from a third party—those must be specified.

Reviewer #1: Yes

Reviewer #2: None

Reviewer #3: Yes
