# Supplementary material for: Position-dependent codon occurrence is associated with patterns of translational efficiency and ribosomal occupancy in the human transcriptome
Source: PLoS Comput Biol. 2026 Jul 20;22(7):e1014501. doi: 10.1371/journal.pcbi.1014501 (PMC13399515; doi:10.1371/journal.pcbi.1014501)
Supplement: Attachment — Submitted filename: Response_to_Reviewers.docx [file pcbi.1014501.s046.docx]

**We thank the reviewers for taking the time to review our last submission. We are pleased that Reviewers #1 and #2 found that our edits were appropriate and thorough, and we are pleased that Reviewer #3 found that the manuscript was improved. We have implemented the suggested additions from Reviewer #3, and added them to the main text as requested.**

**Reviewers' comments:**

Reviewer's Responses to Questions

Reviewer #1: The authors have addressed all my comments and concerns regarding the manuscript.

>>We thank Reviewer #1 for all their helpful suggestions and are glad that we have addressed the comments and concerns.

Reviewer #2: The authors have comprehensively addressed my comments, I appreciate the detailed response.

>>We are glad that we have comprehensively addressed the comments, and we appreciate the suggestions and feel that the manuscript is improved.

Reviewer #3: The paper was improved: There were many comments, and the authors answered most of them. Currently, the most important point for publication is as follows:
Since there are many known patterns and signals at the beginning of the coding regions (mentioned in my previous comments and also cited in the paper), the authors should add *to the main text* (not the supplementary ) a clear analysis the evaluate the contribution of each of the features to their measure and to patterns they report that are correlated with their measure( with a relevant figure). This should be done via analysis of all the new and known features/measures/measurements *together*, for example using regression, partial correlation or any other conventional approach.

There are many relevant previous features that have been reported. They should at least include tAI, GC-content, local mRNA folding, and AA bias. The analysis should include clear quantitative contribution of each of the features to their measure and to patterns they report that are correlated with their measure (e.g. regression coefficient, SHAP, partial correlations).

>>We thank Reviewer #3 for the assurance that the manuscript was improved and that we have answered most of the comments. We have created the final figure that was requested, comparing the features that have been suggested. We have created a combined logistic regression model using POSCO, AAPSSM (our position-dependent amino acid score), GC, GC3, CAI, tAI, delta-G of RNA folding in the 40-nt window 10-nt downstream of the putative start codon or AUG triplet. We used max-min scaling to map every feature to the same scale. Because these features are correlated, we experimented with removing the top features, which were POSCO and AAPSSM. We saw some interesting things when doing this. In particular, the GC3 contribution went from negative to positive when removing POSCO, which, along with the fact that POSCO and GC3 show a correlation, confirms the expectation the POSCO incorporates amino acid bias and GC3 bias.

This is a recalculation of the model with all features at the reviewer’s request, and we have moved this figure to the main text also requested by the reviewer. We removed the previous supplementary figure on this and the table of weights from the previous submission as this are now all included as part of the new Figure 6. The difference in this current version is that we used all features, and that we created a balanced data set from training and testing of the logistic regression model, which to our understanding will improve the model.
