# Supplementary material for: Position-dependent codon occurrence is associated with patterns of translational efficiency and ribosomal occupancy in the human transcriptome
Source: PLoS Comput Biol. 2026 Jul 20;22(7):e1014501. doi: 10.1371/journal.pcbi.1014501 (PMC13399515; doi:10.1371/journal.pcbi.1014501)
Supplement: Peer review history

# Peer review: Decision Letter 0

**DOI:** 10.1371/journal.pcbi.1014501.r001

**Authors:** Ilya Ioshikhes, Martin Schaefer

25 May 2024

Dear Dr Hendrix,

Thank you very much for submitting your manuscript "Position-dependent Codon Usage Bias is Associated with Patterns of Translational Efficiency and Ribosomal Occupancy in the Human Transcriptome" for consideration at PLOS Computational Biology.

As with all papers reviewed by the journal, your manuscript was reviewed by members of the editorial board and by several independent reviewers. In light of the reviews (below this email), we would like to invite the resubmission of a significantly-revised version that takes into account the reviewers' comments.

We cannot make any decision about publication until we have seen the revised manuscript and your response to the reviewers' comments. Your revised manuscript is also likely to be sent to reviewers for further evaluation.

When you are ready to resubmit, please upload the following:

[1] A letter containing a detailed list of your responses to the review comments and a description of the changes you have made in the manuscript. Please note while forming your response, if your article is accepted, you may have the opportunity to make the peer review history publicly available. The record will include editor decision letters (with reviews) and your responses to reviewer comments. If eligible, we will contact you to opt in or out.

[2] Two versions of the revised manuscript: one with either highlights or tracked changes denoting where the text has been changed; the other a clean version (uploaded as the manuscript file).

Important additional instructions are given below your reviewer comments.

Please prepare and submit your revised manuscript within 60 days. If you anticipate any delay, please let us know the expected resubmission date by replying to this email. Please note that revised manuscripts received after the 60-day due date may require evaluation and peer review similar to newly submitted manuscripts.

Thank you again for your submission. We hope that our editorial process has been constructive so far, and we welcome your feedback at any time. Please don't hesitate to contact us if you have any questions or comments.

Sincerely,

Martin H. Schaefer

Guest Editor

PLOS Computational Biology

Ilya Ioshikhes

Section Editor

PLOS Computational Biology

***********************

Reviewer's Responses to Questions

**Comments to the Authors:**

**Please note here if the review is uploaded as an attachment.**

Reviewer #1: All comments have been uploaded as an attachment.

Reviewer #2: Subramanian, Waugh and colleagues describe an analysis of position-dependent codon usage patterns in human genes, using a new metric they call PDCUB. Position-dependent codon usage bias is a well known property of many genomes, and it has multiple possible causes including selection for RNA secondary structure, translational ramps, and nucleotide composition of regions around gene promoters. Despite this, there are no widely accepted measures of position-dependent codon bias and little is known about how this type of bias correlates with functional properties of human genes. This study aims to address this important knowledge gap and provides a number of observations on the correlation of PDCUB with translation, codon adaptation measures and gene ontology classification of genes. Although the results are interesting, I found parts of the paper difficult to follow and I recommend these parts to be thoroughly rewritten before publication.

Major concerns:

1. The section "Optimizing PDCUB weight matrix" unnecessarily complicates what you are trying to do. If I understood this correctly, PDCUB is a simple score based on a match between the sequence of an arbitrary 100-codon window of a gene and a PSSM derived from the empirical frequencies of codons in a 100-codon window downstream of the start codon of human genes? If so, this information is difficult to extract from the manuscript, which instead focuses on describing a convoluted method to predict the start codon of a gene, a task that can be trivially achieved simply by searching for the first ATG substring. I suggest replacing this section with a definition of the PDCUB score, explained in a way that will be accessible to your readers. Once the PDCUB score has been defined, you can then use it to predict start codons and other properties of genes in subsequent sections.

2. The current PDCUB metric seems to conflate codon usage and amino acid usage. Other methods to quantify CUB isolate the effects of codon usage by calculating frequencies of codons relative to other codons for the same amino acid. If PDCUB indeed represents a combination of amino acid usage and codon usage, this should be stated explicitly and prominently in order not to confuse the readers. It might be better to change the name of the metric to avoid confusion.

3. The correlations of PDCUB with GO terms and signal peptides could simply represent the differences in amino acid sequences of genes in GO categories or genes with and without signal peptides. To confirm that the results reflect differences in codon usage bias, you should verify that the same patterns are not observed when applying the PDAAUB metric to the GO and signal peptide classification tasks.

4. The entire description of PDCUB calculation in the methods section should be revisited, the sentence that starts with "bins b of size w" is particularly unclear.

Minor comments:

- In Fig 1B, it would make more sense to invert the colours by displaying the (original - shuffled) scores, rather than as done currently, (shuffled - original).

- In Figure 3A, all models have a very poor predictive power below 1% - perhaps a problem with axis labeling?

- Figure S3 makes it difficult to see the pattern: can this be displayed in the same format as Fig 1?

- p13, it is not unexpected that PDCUB outperforms both PDAAUB and GC3 models given that PDCUB incorporates both amino acid and codon bias information. Would it be possible to check how PDCUB compares with a combined model that includes PDAAUB and GC3?

Reviewer #3: The authors present a novel position dependent codon usage bias (PDCUB) score which is based on the distribution of the first 100 codons of genes. They then study PDCUB and among others they show that it can be used to predict translation-initiating codons with greater accuracy than other models. They report relation to position specific GC content, signal peptide, and more. They suggest that PDCUB defines a spectrum of translational efficiency profiles of tAI. An inverse correlation was found between PDCUB score and ribosomal occupancy in the early transcript. They also examine the relationship between PDCUB intensity and functional enrichment.

This is a potentially interesting study, but additional analysis is required; in addition, the authors should better review and discuss previous studies in the field to better understand the results and their novelty.

1) The study is focused on human genome. I expect such a paper to analyze at least a few organisms from different domains of the tree of life.

2) There are many important previous studies that should be cited and discussed as they may explain some of the reported results. For example, GC content can be explained by mRNA folding profiles and the beginning of the ORF is populated by many overlapping signals related to AUGs, mRNA folding, AA distribution and more (PMID: 25505165, PMID: 32151272).

3) Figure 2. It will be helpful to generate a model (e.g. regressor) based on all models or a partial correlation analysis to understand the independent contribution of the PDCUB when controlling for all other models *together*.

4) Figure 2C: see comments 2), it is easy to explain this region based on the many signals known to appear there.

5) Figure 2A. It was not clear to me if you also check different lengths of the region or only checked 100 codons ? if not- you should clearly try to optimize the length of the region (check shorter and longer lengths). I believe that this length is organism specific (see comments 1)).

6) “Even though PDCUB only considers the first 300 nt of the coding sequence, we observed improved performance with shorter transcripts.” Is it related to the fact that shorter transcripts are highly expressed ? have less splicing ?

7) GO term analysis: it will be helpful to understand if the signal of part of it is simply related to the expression levels (e.g. there are terms that tend to includes genes with higher expression and this may be the reason you see the association).

8) “translational valley” – a pattern some-how similar were reported (for example) for mRNA folding (see point 2) and also PMID: 22050731).

9) A general comment: each p-value should be positive. See for example: “PDCUB score for the coding transcriptome and found significant correlation between the two parameters (2 ≈ 0.3, = 0.0, Supplementary Figure 20).”.

10) Ribo-seq (e.g. figure 4 D). I expect to see larger differences among the different quantiles. I suggest to check additional dataset and also try to check the average of a few datasets.

11) “Notably, while Tuller and others have examined the features of translational ramps in terms of complete transcriptomes for a given species, we have investigated individual subgroups of human transcripts and found that there is a spectrum of tAI trajectories”..“Previous studies have been conflicted about the presence of a translational ramp in mammals, with some reporting no ramp [28] and others reporting a distinct ramp [22]. We find that both results are true depending on where one looks within the PDCUB spectrum, with the highest-PDCUB transcripts having both a strong initial increase in and steady-state average tAI (translational highway), intermediate PDCUB transcripts having a weaker ramp or no ramp...” note that previous studies claims that some of the genes (e.g. based on expression and function) have ramp (or stronger ramp) and some don’t and an analysis not only based on all genes has been reported. Please read carefully the papers mentioned in 2), 8) and ref. [22]. You should better describe the novelty of your paper (which of course exist).

**********

**Have the authors made all data and (if applicable) computational code underlying the findings in their manuscript fully available?**

The PLOS Data policy requires authors to make all data and code underlying the findings described in their manuscript fully available without restriction, with rare exception (please refer to the Data Availability Statement in the manuscript PDF file). The data and code should be provided as part of the manuscript or its supporting information, or deposited to a public repository. For example, in addition to summary statistics, the data points behind means, medians and variance measures should be available. If there are restrictions on publicly sharing data or code —e.g. participant privacy or use of data from a third party—those must be specified.

Reviewer #1: **No:**  Code is available. Data used in figures should be added. Supplementary table 1 was missing.

Reviewer #2: Yes

Reviewer #3: None

**********

PLOS authors have the option to publish the peer review history of their article (what does this mean?). If published, this will include your full peer review and any attached files.

If you choose “no”, your identity will remain anonymous but your review may still be made public.

**Do you want your identity to be public for this peer review?**  For information about this choice, including consent withdrawal, please see our Privacy Policy.

Reviewer #1: **Yes:**  Xavier Hernandez-Alias

Reviewer #2: No

Reviewer #3: No

Figure Files:

While revising your submission, please upload your figure files to the Preflight Analysis and Conversion Engine (PACE) digital diagnostic tool, https://pacev2.apexcovantage.com. PACE helps ensure that figures meet PLOS requirements. To use PACE, you must first register as a user. Then, login and navigate to the UPLOAD tab, where you will find detailed instructions on how to use the tool. If you encounter any issues or have any questions when using PACE, please email us at figures@plos.org.

Data Requirements:

Please note that, as a condition of publication, PLOS' data policy requires that you make available all data used to draw the conclusions outlined in your manuscript. Data must be deposited in an appropriate repository, included within the body of the manuscript, or uploaded as supporting information. This includes all numerical values that were used to generate graphs, histograms etc.. For an example in PLOS Biology see here: http://www.plosbiology.org/article/info%3Adoi%2F10.1371%2Fjournal.pbio.1001908#s5.

Reproducibility:

To enhance the reproducibility of your results, we recommend that you deposit your laboratory protocols in protocols.io, where a protocol can be assigned its own identifier (DOI) such that it can be cited independently in the future. Additionally, PLOS ONE offers an option to publish peer-reviewed clinical study protocols. Read more information on sharing protocols at https://plos.org/protocols?utm_medium=editorial-email&utm_source=authorletters&utm_campaign=protocols

Attachment Submitted filename: review_PCOMPBIOL-D-23-01868.docx

# Author comment: Author response to Decision Letter 1

**DOI:** 10.1371/journal.pcbi.1014501.r002

3 Dec 2024

Attachment Submitted filename: review_PCOMPBIOL-D-23-01868_2.docx

# Peer review: Decision Letter 1

**DOI:** 10.1371/journal.pcbi.1014501.r003

**Authors:** Ilya Ioshikhes, Martin Schaefer

17 Jan 2025

PCOMPBIOL-D-23-01868R1

Position-dependent Codon Usage Bias is Associated with Patterns of Translational Efficiency and Ribosomal Occupancy in the Human Transcriptome

PLOS Computational Biology

Dear Dr. Hendrix,

Thank you for submitting your manuscript to PLOS Computational Biology. After careful consideration, we feel that it has merit but does not fully meet PLOS Computational Biology's publication criteria as it currently stands. Therefore, we invite you to submit a revised version of the manuscript that addresses the points raised during the review process.

Please submit your revised manuscript within 60 days Mar 19 2025 11:59PM. If you will need more time than this to complete your revisions, please reply to this message or contact the journal office at ploscompbiol@plos.org. When you're ready to submit your revision, log on to https://www.editorialmanager.com/pcompbiol/ and select the 'Submissions Needing Revision' folder to locate your manuscript file.

Please include the following items when submitting your revised manuscript:

* A rebuttal letter that responds to each point raised by the editor and reviewer(s). You should upload this letter as a separate file labeled 'Response to Reviewers'. This file does not need to include responses to formatting updates and technical items listed in the 'Journal Requirements' section below.

* A marked-up copy of your manuscript that highlights changes made to the original version. You should upload this as a separate file labeled 'Revised Manuscript with Track Changes'.

* An unmarked version of your revised paper without tracked changes. You should upload this as a separate file labeled 'Manuscript'.

If you would like to make changes to your financial disclosure, competing interests statement, or data availability statement, please make these updates within the submission form at the time of resubmission. Guidelines for resubmitting your figure files are available below the reviewer comments at the end of this letter

We look forward to receiving your revised manuscript.

Kind regards,

Martin H. Schaefer

Guest Editor

PLOS Computational Biology

Ilya Ioshikhes

Section Editor

PLOS Computational Biology

**Additional Editor Comments:**

As you will see from the reviewers’ comments, there are still doubts regarding how rigorously their initial concerns have been addressed. Specifically, the criticism persists that some claims require additional support through further analyses and certain parts of the manuscript lack clarity.

While we understand that the authors faced challenges in addressing all comments within the allocated timeframe, we must insist in a thorough resolution of the reviewers' main concerns in a subsequent revision. Should additional time be required for resubmission, we are happy to discuss an extension of the deadline with the authors.

**Reviewers' comments:**

Reviewer's Responses to Questions

**Comments to the Authors:**

**Please note here if the review is uploaded as an attachment.**

Reviewer #1: Thank you for the efforts in addressing the reviews. Most of my comments are now correctly addressed, and I believe the article is now improved. Nonetheless the authors themselves mention several times in their response that some of the reviewers' suggestions (although mostly minor) could not be implemented due to the lack of time. I believe a second revision would grant them the needed time for this.

In particular, my only major concern is the lack of evidence for the statement "PDCUB is Associated with Patterns of Translational Efficiency". The authors only compare PDCUB to estimates of TE like tAI or CAI, but there is no data supporting that PDCUB correlates with protein-to-mRNA ratios or Ribo-seq/RNA-seq ratios (aka Translation

Efficiency). The authors actually mention in the revision that "We have also compared to protein to mRNA ratios and found no significant correlation", and they even suggest that the effect is more on the distribution of ribosome in the transcript rather than in the overall TE. Therefore, the claim that PDCUB is associated with TE is not sufficiently supported. To prove the claim, one would need to show the correlation R2 of PDCUB vs experimental data of TE (either from protein/mRNA or Ribo-seq/RNA-seq). And to grasp how much of this TE variance is better explained by PDCUB compared to other metrics, compare the obtained R2 with "tAI vs TE" and "CAI vs TE".

Reviewer #2: The authors seem to have largely ignored comments in my original review, and I do not recommend publication.

In the first round of review, I recommended that the authors remove or thoroughly rewrite the section that deals with prediction of start codons. The authors now added some text to this section but this does not address my concerns. I find this part of the manuscript to be of little interest, because (1) we already know where start codons are, and there is no practical benefit in predicting start codons based on codon bias; (2) the ability to predict a start codon from codon bias is not surprising, given the known variation of GC content, amino acid content and codon bias along genes (as shown in previous publications and in Figure 1 of the present mauscript); (3) it is unlikely that codon bias in a 100-codon window mechanistically influences start codon selection. Instead of focusing on the prediction of start codons, it would be better to document the position-dependent codon bias, define the PDCUB metric more clearly, and analyse the possible causes and consequences of PDCUB.

In answer to my major comments 2 and 3, the rebuttal letter reiterates some points made in the original manuscript, but it is not clear if any new analyses were made or if changes were introduced in the manuscript in response to these comments. It still seems that a good part of the observations reported in the manuscript might be caused by patterns of amino acid usage along proteins.

Minor comment:

Figure 3B - there are errors in the figure legend: "Frame 1 triplet" is repeated three times instead of Frame 1, 2 ,and 3; and the dashed line that indictaes the start codon is blue in the legend but green in the plot.

Reviewer #3: The paper was improved but still there is a lot of work (I am spending a lot of time on this review and hope that the authors will take my comments seriously and update the paper comprehensively):

1) My prev. comment 1: I do think that you should add at least one additional organism. If this is so challenging it may suggest that the methodology & conclusions are problematic and cannot be generalized. If there are differences among other organisms this is clearly interesting and should be reported.

2) My prev. comment 2: I must say that your answer is not 100% clear to me (see comments below). In any case, you should add a clear discussion about this point with relevant references to the discussion section.

We agree that the mRNA folding would be the most plausible connection to the PDCUB signal. If the 5’ end is to show greater mRNA folding, the only place where one can add GC nucleotides to favor folding stability is in the third position of codons. However, not all GC3 codons are favored. In the end, it may be difficult to uncouple the mRNA folding and the codon usage bias at the beginning of coding regions, and they may describe the same underlying trend.

- there are combinations of constraints on the coding sequence and the fact that not all GC3 codons are favored does not prove/disprove that mRNA folding is not an important variable here. In addition, folding is effected by combination of codons and just looking at GC3 codon is not good enough.

However, given that the tAI is increased for the high PDCUB transcripts, there appears to be a correlation between the PDCUB pattern and translational efficiency of the codons, which would be distinct from mRNA folding.

The correlation is far from perfect; thus, this can clearly explain only part of the signal.

Furthermore, the lower ribosomal occupancy for high PDCUB codon regions suggest a faster moving ribosome, which is at odds with the coding sequence being folded up in base pairs.

The mRNA folding can affect ribosomal density and movement in various ways. It can increase and decrease the density in various ways.

** Note that I mentioned other patterns (other than folding) at the beginning of the ORF. You did not consider this at all.

3) My previous comment 3: The fact that you "did not have time" is not an "excuse". You can ask the editor for an extension and finish all the comments (this one and others). It does not make sense to publish a paper with so many " holes" and problems.

4) My previous point 4: please see my comment 2) above.

5) My previous point 5: see my comment 1) above. The length is clearly organism specific, and it will be good to show this (it is strange to me that you unable to do this).

6) My previous point 7: Your answer is not clear to me.

>>Thank you for this question. It is hard to assess “expression” in general, as it is of course tissue- and celltype-specific. We did not see a correlation between PDCUB and protein-tomRNA ratio based on our analysis of protein and RNA data.

*did you check relations to protein levels alone and/or mRNA levels along (you can use mean over tissues). If there is not relations it is very strange in my opinion.

We hypothesize that PDCUB has more to do with efficient initiation, rather than overall efficient translation. This is consistent with our discussion on the genes with the highest PDCUB require more rapid expression as dictated by regulation of timing (e.g. during development or immune response).

Efficient initiation usually (almost always, in natural conditions) will have a significant effect on translation.

This being said, expression level can mean a lot of things, and it is possible these effects could be tissuespecific, and the analysis would be a very large undertaking to tease apart tissue-specific expression and PDCUB. For this reason, we have decided to address this comment by adding this as a limitation to our study in the second to last paragraph of the discussion.

You can easily check if there is a relation by comparing to mean expression over all tissues (there is high correlation between the expression levels of genes among different tissues; thus, this is a very good proxy to typical tissue specific expression).

7) My previous comment 9: it is very important to add a p-value. You should write for example p<10^(-40) if this is your resolution.

8) My previous comment 11: you still do not describe accurately the previous studies that have reported scores for single genes and groups of genes (contrary to what you wrote).

**********

**Have the authors made all data and (if applicable) computational code underlying the findings in their manuscript fully available?**

The PLOS Data policy requires authors to make all data and code underlying the findings described in their manuscript fully available without restriction, with rare exception (please refer to the Data Availability Statement in the manuscript PDF file). The data and code should be provided as part of the manuscript or its supporting information, or deposited to a public repository. For example, in addition to summary statistics, the data points behind means, medians and variance measures should be available. If there are restrictions on publicly sharing data or code —e.g. participant privacy or use of data from a third party—those must be specified.

Reviewer #1: Yes

Reviewer #2: None

Reviewer #3: Yes

**********

PLOS authors have the option to publish the peer review history of their article (what does this mean?). If published, this will include your full peer review and any attached files.

If you choose “no”, your identity will remain anonymous but your review may still be made public.

**Do you want your identity to be public for this peer review?**  For information about this choice, including consent withdrawal, please see our Privacy Policy.

Reviewer #1: **Yes:**  Xavier Hernandez Alias

Reviewer #2: No

Reviewer #3: No

[NOTE: If reviewer comments were submitted as an attachment file, they will be attached to this email and accessible via the submission site. Please log into your account, locate the manuscript record, and check for the action link "View Attachments". If this link does not appear, there are no attachment files.]

**Figure resubmission:**

While revising your submission, please upload your figure files to the Preflight Analysis and Conversion Engine (PACE) digital diagnostic tool, https://pacev2.apexcovantage.com/. PACE helps ensure that figures meet PLOS requirements. To use PACE, you must first register as a user. Registration is free. Then, login and navigate to the UPLOAD tab, where you will find detailed instructions on how to use the tool. If you encounter any issues or have any questions when using PACE, please email PLOS at figures@plos.org. Please note that Supporting Information files do not need this step. If there are other versions of figure files still present in your submission file inventory at resubmission, please replace them with the PACE-processed versions.

**Reproducibility:**

To enhance the reproducibility of your results, we recommend that authors of applicable studies deposit laboratory protocols in protocols.io, where a protocol can be assigned its own identifier (DOI) such that it can be cited independently in the future. Additionally, PLOS ONE offers an option to publish peer-reviewed clinical study protocols. Read more information on sharing protocols at https://plos.org/protocols?utm_medium=editorial-email&utm_source=authorletters&utm_campaign=protocols

# Author comment: Author response to Decision Letter 2

**DOI:** 10.1371/journal.pcbi.1014501.r004

25 Aug 2025

Attachment Submitted filename: reviews.docx

# Peer review: Decision Letter 2

**DOI:** 10.1371/journal.pcbi.1014501.r005

**Authors:** Ilya Ioshikhes, Martin Schaefer

22 Oct 2025

PCOMPBIOL-D-23-01868R2

Position-dependent Codon Occurrence is Associated with Patterns of Translational Efficiency and Ribosomal Occupancy in the Human Transcriptome

PLOS Computational Biology

Dear Dr. Hendrix,

Thank you for submitting your manuscript to PLOS Computational Biology. After careful consideration, we feel that it has merit and largely meets PLOS Computational Biology’s publication criteria. Only minor revisions are needed to address the remaining point raised during the review process. Therefore, we invite you to submit a revised version of the manuscript that addresses these comments.

Please submit your revised manuscript within 30 days Dec 22 2025 11:59PM. If you will need more time than this to complete your revisions, please reply to this message or contact the journal office at ploscompbiol@plos.org. When you're ready to submit your revision, log on to https://www.editorialmanager.com/pcompbiol/ and select the 'Submissions Needing Revision' folder to locate your manuscript file.

Please include the following items when submitting your revised manuscript:

* A rebuttal letter that responds to each point raised by the editor and reviewer(s). You should upload this letter as a separate file labeled 'Response to Reviewers'. This file does not need to include responses to formatting updates and technical items listed in the 'Journal Requirements' section below.

* A marked-up copy of your manuscript that highlights changes made to the original version. You should upload this as a separate file labeled 'Revised Manuscript with Track Changes'.

* An unmarked version of your revised paper without tracked changes. You should upload this as a separate file labeled 'Manuscript'.

If you would like to make changes to your financial disclosure, competing interests statement, or data availability statement, please make these updates within the submission form at the time of resubmission. Guidelines for resubmitting your figure files are available below the reviewer comments at the end of this letter.

We look forward to receiving your revised manuscript.

Kind regards,

Martin H. Schaefer

Guest Editor

PLOS Computational Biology

Ilya Ioshikhes

Section Editor

PLOS Computational Biology

**Additional Editor Comments :**

The reviewers agree that the manuscript has improved substantially, and most concerns have been addressed. One reviewer, however, raises one remaining point concerning the inclusion of an analysis as a main figure. This analysis appears to be largely available in the supplementary material, and we believe that addressing this comment should be readily achievable by moving and, if necessary, extending or integrating those analyses into a main-text figure with concise description.

**Journal Requirements:**

1) Thank you for stating "Code is available at https://github.com/hendrixlab/POSCO." This link reaches a 404 error page. Please amend this to a new link or provide further details to locate the data.

Note: If the reviewer comments include a recommendation to cite specific previously published works, please review and evaluate these publications to determine whether they are relevant and should be cited. There is no requirement to cite these works unless the editor has indicated otherwise.

**Reviewers' comments:**

Reviewer's Responses to Questions

Reviewer #1: The authors have addressed all my comments and concerns regarding the manuscript.

Reviewer #2: The authors have comprehensively addressed my comments, I appreciate the detailed response.

Reviewer #3: The paper was improved: There were many comments, and the authors answered most of them. Currently, the most important point for publication is as follows:

Since there are many known patterns and signals at the beginning of the coding regions (mentioned in my previous comments and also cited in the paper), the authors should add *to the main text* (not the supplementary ) a clear analysis the evaluate the contribution of each of the features to their measure and to patterns they report that are correlated with their measure( with a relevant figure). This should be done via analysis of all the new and known features/measures/measurements *together*, for example using regression, partial correlation or any other conventional approach.

There are many relevant previous features that have been reported. They should at least include tAI, GC-content, local mRNA folding, and AA bias. The analysis should include clear quantitative contribution of each of the features to their measure and to patterns they report that are correlated with their measure (e.g. regression coefficient, SHAP, partial correlations).

**********

**Have the authors made all data and (if applicable) computational code underlying the findings in their manuscript fully available?**

The PLOS Data policy requires authors to make all data and code underlying the findings described in their manuscript fully available without restriction, with rare exception (please refer to the Data Availability Statement in the manuscript PDF file). The data and code should be provided as part of the manuscript or its supporting information, or deposited to a public repository. For example, in addition to summary statistics, the data points behind means, medians and variance measures should be available. If there are restrictions on publicly sharing data or code —e.g. participant privacy or use of data from a third party—those must be specified.

Reviewer #1: Yes

Reviewer #2: Yes

Reviewer #3: Yes

**********

PLOS authors have the option to publish the peer review history of their article (what does this mean?). If published, this will include your full peer review and any attached files.

If you choose “no”, your identity will remain anonymous but your review may still be made public.

**Do you want your identity to be public for this peer review?**  For information about this choice, including consent withdrawal, please see our Privacy Policy.

Reviewer #1: **Yes:**  Xavier Hernandez Alias

Reviewer #2: No

Reviewer #3: No

[NOTE: If reviewer comments were submitted as an attachment file, they will be attached to this email and accessible via the submission site. Please log into your account, locate the manuscript record, and check for the action link "View Attachments". If this link does not appear, there are no attachment files.]

**Figure resubmission:**

-->While revising your submission, we strongly recommend that you use PLOS’s NAAS tool (https://ngplosjournals.pagemajik.ai/artanalysis) to test your figure files. NAAS can convert your figure files to the TIFF file type and meet basic requirements (such as print size, resolution), or provide you with a report on issues that do not meet our requirements and that NAAS cannot fix.

After uploading your figures to PLOS’s NAAS tool - https://ngplosjournals.pagemajik.ai/artanalysis, NAAS will process the files provided and display the results in the "Uploaded Files" section of the page as the processing is complete. If the uploaded figures meet our requirements (or NAAS is able to fix the files to meet our requirements), the figure will be marked as "fixed" above. If NAAS is unable to fix the files, a red "failed" label will appear above. When NAAS has confirmed that the figure files meet our requirements, please download the file via the download option, and include these NAAS processed figure files when submitting your revised manuscript.

**Reproducibility:**

To enhance the reproducibility of your results, we recommend that authors of applicable studies deposit laboratory protocols in protocols.io, where a protocol can be assigned its own identifier (DOI) such that it can be cited independently in the future. Additionally, PLOS ONE offers an option to publish peer-reviewed clinical study protocols. Read more information on sharing protocols at https://plos.org/protocols?utm_medium=editorial-email&utm_source=authorletters&utm_campaign=protocols

# Author comment: Author response to Decision Letter 3

**DOI:** 10.1371/journal.pcbi.1014501.r006

23 Jun 2026

Attachment Submitted filename: Response_to_Reviewers.docx

# Editor report: Decision Letter 3

**DOI:** 10.1371/journal.pcbi.1014501.r007

**Authors:** Ilya Ioshikhes, Martin Schaefer

26 Jun 2026

Dear Dr. Hendrix,

We are pleased to inform you that your manuscript 'Position-dependent Codon Occurrence is Associated with Patterns of Translational Efficiency and Ribosomal Occupancy in the Human Transcriptome' has been provisionally accepted for publication in PLOS Computational Biology.

Before your manuscript can be formally accepted you will need to complete some formatting changes, which you will receive in a follow up email. A member of our team will be in touch with a set of requests.

Please note that your manuscript will not be scheduled for publication until you have made the required changes, so a swift response is appreciated.

IMPORTANT: The editorial review process is now complete. PLOS will only permit corrections to spelling, formatting or significant scientific errors from this point onwards. Requests for major changes, or any which affect the scientific understanding of your work, will cause delays to the publication date of your manuscript.

Should you, your institution's press office or the journal office choose to press release your paper, you will automatically be opted out of early publication. We ask that you notify us now if you or your institution is planning to press release the article. All press must be co-ordinated with PLOS.

Thank you again for supporting Open Access publishing; we are looking forward to publishing your work in PLOS Computational Biology.

Best regards,

Martin H. Schaefer

Guest Editor

PLOS Computational Biology

Ilya Ioshikhes

Section Editor

PLOS Computational Biology

***********************************************************

# Editor report: Acceptance letter

**DOI:** 10.1371/journal.pcbi.1014501.r008

**Authors:** Ilya Ioshikhes, Martin Schaefer

PCOMPBIOL-D-23-01868R3

Position-dependent Codon Occurrence is Associated with Patterns of Translational Efficiency and Ribosomal Occupancy in the Human Transcriptome

Dear Dr Hendrix,

I am pleased to inform you that your manuscript has been formally accepted for publication in PLOS Computational Biology. Your manuscript is now with our production department and you will be notified of the publication date in due course.

The corresponding author will soon be receiving a typeset proof for review, to ensure errors have not been introduced during production. Please review the PDF proof of your manuscript carefully, as this is the last chance to correct any errors. Please note that major changes, or those which affect the scientific understanding of the work, will likely cause delays to the publication date of your manuscript.

Soon after your final files are uploaded, unless you have opted out, the early version of your manuscript will be published online. The date of the early version will be your article's publication date. The final article will be published to the same URL, and all versions of the paper will be accessible to readers.

For Research, Software, and Methods articles, you will receive an invoice from PLOS for your publication fee after your manuscript has reached the completed accept phase. If you receive an email requesting payment before acceptance or for any other service, this may be a phishing scheme. Learn how to identify phishing emails and protect your accounts at https://explore.plos.org/phishing.

Thank you again for supporting PLOS Computational Biology and open-access publishing. We are looking forward to publishing your work!

With kind regards,

Kannan R K Kuppusamy, B.TECH BIOTECHNOLOGY

PLOS Computational Biology | Carlyle House, Carlyle Road, Cambridge CB4 3DN | United Kingdom ploscompbiol@plos.org | Phone +44 (0) 1223-442824 | ploscompbiol.org | @PLOSCompBiol
